# A Versatile Multiplexed Immunofluorescence Strategy for Efficient, Host‐Independent, and Scalable Spatial Protein Profiling

**DOI:** 10.1002/smtd.202600009

**Published:** 2026-06-17

**Authors:** Phuong Nguyen, Hongqiang Ma, Dimitrios S. Gotsis, Maomao Chen, Chaojie Zhang, Tushar Talukder Showrav, Marc Schwartz, Brenda Diergaarde, Robert E. Schoen, Rhonda M. Brand, Hua Zhang, Yang Liu

**Affiliations:** ^1^ Grainger College of Engineering Department of Bioengineering University of Illinois Urbana‐Champaign Urbana Illinois USA; ^2^ Grainger College of Engineering Department of Electrical and Computer Engineering University of Illinois Urbana‐Champaign Urbana Illinois USA; ^3^ Department of Medicine Division of Gastroenterology Hepatology and Nutrition University of Pittsburgh University of Pittsburgh Medical Center Pittsburgh Pennsylvania USA; ^4^ Department of Human Genetics University of Pittsburgh School of Public Health Pittsburgh Pennsylvania USA; ^5^ Hillman Cancer Center University of Pittsburgh Medical Center (UPMC) Pittsburgh Pennsylvania USA; ^6^ Department of Medicine Division of Hematology/Oncology University of Pittsburgh School of Medicine Pittsburgh Pennsylvania USA; ^7^ Cancer Center at Illinois Beckman Institute for Advanced Science and Technology University of Illinois Urbana‐Champaign Urbana Illinois USA

**Keywords:** cyclic immunofluorescence, host‐independent labeling, immunofluorescence staining, spatial proteomics

## Abstract

Immunofluorescence (IF) remains a central method for high‐content spatial biology, but current approaches face several technical barriers. Indirect two‐step IF is the most widely used but constrained by host‐species requirements and lengthy workflows. Direct IF avoids this constraint but requires chemical conjugation of each primary antibody to a fluorophore, resulting in weaker signals, fixed antibody–fluorophore pairings, and reduced flexibility. Antibody‐nanobody complexes provide a promising host‐independent solution but have been limited by inconsistent performance and reduced efficiency. Commercial spatial biology platforms achieve high‐plex imaging, but many rely on costly custom reagents or specialized microfluidics instrumentation, limiting broad accessibility. We introduce umIF, a strategy that leverages macromolecular crowding to enhance labeling efficiency across diverse IF workflows. It enables robust, host‐independent multiplexing with antibody‐nanobody complexes, while also improving performance across direct, one‐step, and two‐step IF workflows, including detection of weak targets. We demonstrate both single‐ and multicycle umIF in human tissue samples and mouse models, revealing epithelial, stromal, immune, and epigenetic organization in normal and disease contexts. umIF provides a versatile and accessible method for scalable spatial proteomics, lowering barriers to adoption in both research and clinical laboratories.

## Graphical abstract

Crowding‐enhanced multiplex immunofluorescence (umIF) provides a versatile strategy for scalable spatial profiling in FFPE tissues. By improving labeling efficiency across direct, one‐step, and two‐step IF formats, umIF enables host‐species‐independent use of primary antibodies and antibody–nanobody complexes, detection of weak targets, and multicycle spatial mapping of epithelial, stromal, immune, and epigenetic organization in human and mouse tissues.

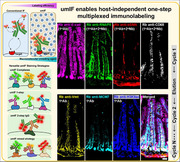

## 1 Introduction

Spatial biology has transformed our ability to study complex tissue microenvironment, enabling high‐dimensional mapping of protein expression and cell states within an intact histological context. Immunofluorescence (IF)‐based spatial proteomics on formalin‐fixed paraffin‐embedded (FFPE) tissue provides a powerful bridge between molecular biology and clinical pathology. Because FFPE is the standard format for patient specimens worldwide, highly multiplexed IF on FFPE sections offers a unique opportunity to link spatial phenotypes to clinical outcomes.

Commercial microfluidics‐based spatial biology platforms provide partial solutions, but they require specialized instrumentation and proprietary reagents, restricting access for most laboratories. Thus, there remains a critical need for methods that retain the accessibility of traditional antibody‐based IF while delivering the efficiency, robustness, and scalability required for high‐plex spatial proteomics.

Recent advances such as Cyclic Immunofluorescence (CyCIF) [1], tissue‐based CyCIF (t‐CyCIF [2]), iterative indirect immunofluorescence imaging (4i) [3], and IBEX [4] extend the use of standard antibodies to the multiplexed IF workflow. These methods have substantially improved the accessibility of high‐plex spatial proteomics, enabling broader adoption in research laboratories. However, these methods remain constrained by the fundamental trade‐offs between indirect and direct IF, including host‐species restrictions, limited fluorophore flexibility, and reduced labeling efficiency, which can compromise reproducibility and often prevent the use of the best‐performing primary antibodies.

Indirect two‐step labeling, where primary IgG antibodies are detected by fluorophore‐conjugated secondary IgG, yields strong signal amplification, broad antibody compatibility, and flexible fluorophore selection. However, it is limited by host‐species restrictions. Typically, only one or two primaries (1°Ab) from different species (commonly mouse and rabbit) can be used simultaneously without cross‐reactivity. This limitation often forces researchers to combine highly efficient antibodies (e.g., rabbit monoclonal) with weaker antibodies from other species (e.g., mouse) [5], which can reduce assay performance and prevent the use of the best‐validated primary antibodies across the full panel [6].

Direct IF, by contrast, removes host‐species constraints through dye‐conjugated primaries. However, this approach introduces a different set of trade‐offs. The fluorophore–antibody pairing is fixed, which reduces experimental flexibility and may affect antigen affinity, and signal intensity is further limited by the absence of secondary antibody amplification. In addition, commercially available dye‐conjugated primaries cover only a subset of antibodies, restricting the diversity of targets accessible to direct IF. Although custom conjugation can, in principle, expand fluorophore options, it is generally inefficient and costly, as it requires high antibody concentrations yet achieves modest labeling efficiencies (typically only 20%–35% even at milligram‐per‐milliliter levels), adding considerable cost and effort for large‐scale multiplexed applications.

Recombinant camelid single‐domain antibodies (nanobodies) (2°Nb) offer a streamlined “one‐step” alternative to conventional IF. In this approach, a fluorophore‐labeled nanobody is premixed with the primary antibody [7] (1°Ab), and the resulting nanobody complex (1°Ab+2°Nb) is applied in a single incubation. Unlike conventional secondary IgGs, nanobodies are about ten times smaller and lack an Fc domain, which improves tissue penetration and reduces Fc receptor–mediated background. The pre‐mixed antibody‐nanobody format overcomes host‐species constraints and enables flexible pairing of fluorophore with primary antibody simply by altering the secondary nanobody fluorophore, which is relatively inexpensive.

Despite these advantages, nanobody‐complex‐based one‐step IF has seen limited adoption for simultaneous detection of multiple targets in a single incubation, largely due to reduced labeling efficiency and inconsistent performance. Off‐target cross‐binding and reversible interactions can further compromise sensitivity and specificity [8]. Post‐fixation can stabilize nanobody complexes [7], but it interferes with efficient signal removal in multi‐cycle IF. In addition, signal instability and variability with polyclonal primaries can further limit reproducibility [9]. Moreover, reported applications of nanobody complexes have often relied on engineered secondary nanobodies conjugated to oligonucleotides [10, 11] or cleavable linkers [12]—approaches that demand advanced expertise in bioconjugation and protein purification, thereby limiting accessibility to most laboratories.

To address these challenges, we developed umIF, a host‐independent immunofluorescence strategy that enhances the efficiency, flexibility, and practicality of existing IF workflows. By introducing an optimized co‐incubation buffer, umIF enhances labeling efficiency and enables reliable detection of multiple antibody‐nanobody complexes in a single step. The buffer also minimizes background and allows shorter, more efficient elution steps in multi‐cycle high‐plex immunofluorescence workflows, maintaining high sample quality across repeated cycles under the tested conditions. Furthermore, umIF enables straightforward assembly of antibody panels using the most efficient, commercially available unconjugated primary antibodies (e.g., well‐validated rabbit monoclonal antibodies) paired with dye‐conjugated secondary nanobodies, thereby simplifying antibody panel design while ensuring high signal efficiency. Beyond one‐step antibody‐nanobody labeling, umIF also enhances conventional direct and two‐step IF workflows, further broadening its practical utility and improving the use of commercially available primary antibodies.

A key strength of umIF is its versatility. The method performs robustly with both monoclonal and polyclonal antibodies and integrates seamlessly with direct, one‐step pre‐mixing, or conventional two‐step IF workflows. Across these formats, umIF improves labeling efficiency and facilitates detection of weak targets while remaining compatible with both single‐cycle and multi‐cycle imaging workflows. Collectively, these features establish umIF as a practical and broadly applicable method to enhance the efficiency of high‐plex spatial proteomics. By improving the efficiency and flexibility of both single‐cycle IF and cyclic IF workflows, including iterative immunofluorescence approaches such as 4i, umIF lowers barriers to adoption across research and clinical laboratories, enabling scalable high‐content spatial protein profiling. We further demonstrate its broad utility in high‐plex spatial protein profiling of both human and mouse tissues in normal and various disease contexts.

## 2 Results

### 2.1 umIF Enables Host‐Independent One‐Step Multiplexed Immunofluorescence Staining

**FIGURE 1:**
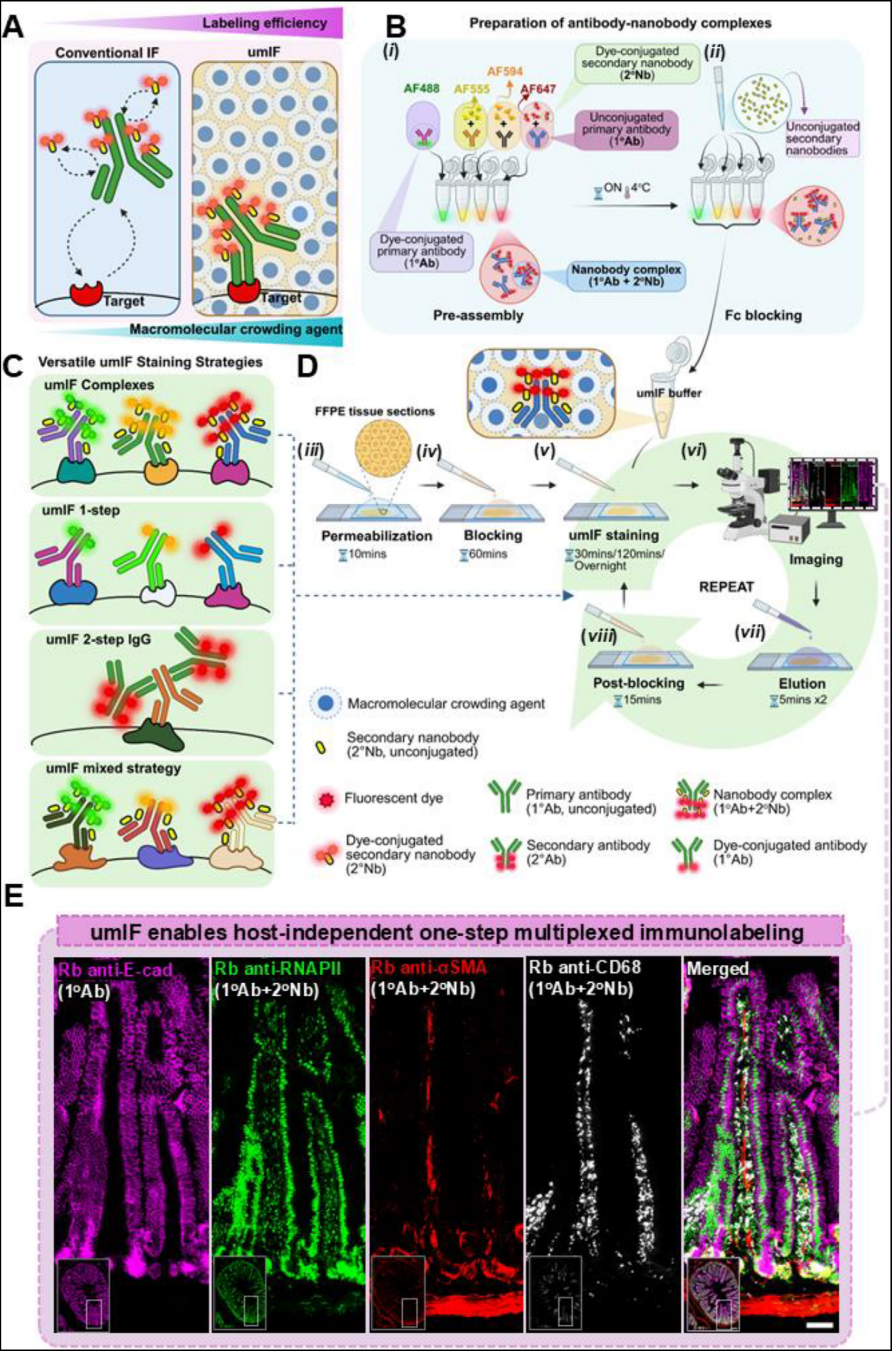
Overview of the umIF strategy and workflow. (A) Schematic illustration of the proposed effect of macromolecular crowding in umIF. Compared with conventional IF, the crowding‐enhanced buffer increases the effective local concentration of labeling reagents and promotes more efficient target labeling during staining. (B) Preparation of antibody‐nanobody complexes (1°Ab + 2°Nb) for umIF. (i) Dye‐conjugated secondary nanobodies (2°Nb) are premixed with unconjugated primary antibodies (1°Ab) or combined with dye‐conjugated primary antibodies. (ii) Excess unlabeled secondary nanobodies are then added to block residual Fc sites and minimize cross‐binding before multiplexed staining. (C) Versatile umIF staining strategies supported by the workflow, including umIF complexes (1°Ab + 2°Nb), direct one‐step staining with dye‐conjugated primary antibodies (umIF 1‐step), two‐step IgG staining (umIF 2‐step IgG), and mixed‐format staining within the same cycle. (D) Overall umIF workflow for FFPE tissue sections, including permeabilization, blocking, staining in crowding‐enhanced umIF buffer, imaging, elution, and post‐blocking for repeated multicycle imaging. (E) Representative single‐cycle umIF staining of mouse small intestine demonstrating host‐independent same‐species multiplexing. Four rabbit‐derived antibodies were used in the same cycle: anti‐E‐cadherin (magenta, direct 1°Ab), anti‐RNA polymerase II‐S2P (green, 1°Ab + 2°Nb), anti‐α‐smooth muscle actin (αSMA; red, 1°Ab + 2°Nb), and anti‐CD68 (gray, 1°Ab + 2°Nb). Scale bars, 50 µm (main panels) and 200 µm (insets).

The umIF enables one‐step simultaneous detection of multiple same‐species antibodies within a single staining cycle, as illustrated in Figure 1A, B. Unlike conventional IF methods constrained by host‐species requirements, umIF employs complexes formed between unconjugated primary IgG antibodies (1° Abs) and dye‐conjugated secondary nanobodies (2° Nbs) in an optimized co‐incubation buffer. This strategy allows flexible pairing of fluorophores with primary antibody targets by varying the fluorophore on the secondary nanobody, effectively merging the versatility of indirect IF with the host‐independence of direct IF. For example, in mouse small intestine (Figure 1C), three rabbit‐derived antibodies, including RNAPII‐S2P (nuclear), αSMA (cytoplasmic), and CD68 (surface), were simultaneously detected using antibody‐nanobody complexes (1°Ab + 2°Nb; hereafter referred to as *complexes*), producing distinct and specific staining patterns without host‐species limitations.

A key innovation of umIF (Figure 1A) is the incorporation of macromolecular crowding agents into the co‐incubation buffer for the multiplex immunofluorescence labeling. Macromolecular crowding has long been recognized to modulate molecular interactions through excluded‐volume effects, which increase local concentration and alter association equilibrium [13, 14, 15, 16, 17]. Extensive prior work has shown that inert polymers such as dextran or PEG can enhance receptor binding and enzymatic reactions directly on cell surfaces, sometimes by several hundred‐fold [18]. Building on this concept, we adapted crowding principles to multiplexed IF to improve labeling efficiency. In umIF, we optimized the concentration of crowding agents, including dextran sulfate and Denhardt's solution, to enhance labeling performance across surface and nuclear markers (Figure S1). The resulting crowding‐enhanced buffer improves labeling efficiency and increases fluorescence intensity within the same incubation time. As shown in Figure S2, umIF supports consistent staining performance across a broad range of incubation times, providing the basis for the time‐course evaluation described below.

Moreover, umIF is fully compatible with the conventional direct IF workflow using dye‐conjugated primary antibodies, allowing mixed use of *complexes* and the direct dye‐conjugated primaries (1°Ab) within the same cycle. This hybrid flexibility is demonstrated in mouse intestinal tissue (Figure 1C and Figure S3), where both direct and one‐step complexes labeling approaches produced robust staining across different targets. Together, these results establish umIF as a robust, host‐independent, and flexible platform for one‐step multiplexed IF labeling, forming the basis for subsequent evaluation of its labeling efficiency across staining formats and conditions.

### 2.2 The Crowding‐Enhanced Buffer Primarily Improves Signal Accumulation During Staining

To determine whether the dominant measurable effect of the crowding‐enhanced buffer occurs during antibody association or during post‐labeling retention, we performed time‐course experiments under conventional and crowding‐enhanced conditions (umIF) using both dye‐conjugated primary antibodies (1°Ab) and antibody‐nanobody complexes (1°Ab + 2°Nb).

**FIGURE 2:**
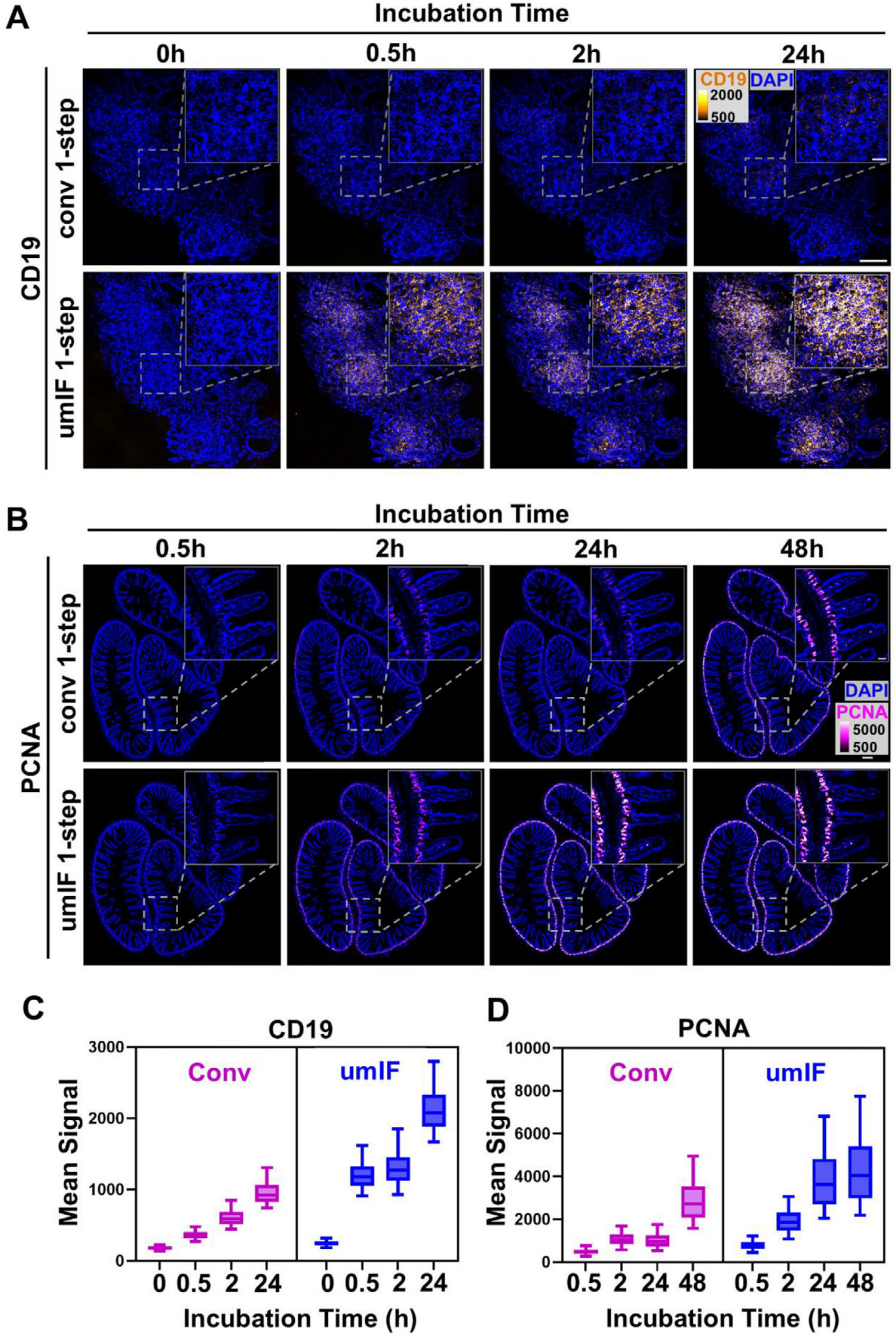
The crowding‐enhanced umIF buffer promotes faster signal accumulation during staining. (A, B) Representative immunofluorescence images of (A) CD19 in an inflamed region of human colon tissue with ulcerative colitis and (B) PCNA in mouse intestine tissue, stained with dye‐conjugated primary antibodies and overlaid with DAPI nuclear staining under conventional and umIF buffer conditions at different incubation times (CD19: 0, 0.5, 2, and 24 h; PCNA: 0.5, 2, 24, and 48 h). (C, D) Quantification of mean fluorescence intensity for CD19 and PCNA, presented as box‐and‐whisker plots (5th–95th percentile). For CD19, fluorescence intensities (mean ± SD, a.u.) were 182.4 ± 26.12 (n = 200 ROIs), 380.7 ± 167.6 (n = 200 ROIs), 618.8 ± 176.5 (n = 200 ROIs), and 965.0 ± 183.1 (n = 200 ROIs) under conventional conditions at 0, 0.5, 2, and 24 h, respectively, and 246.7 ± 40.16 (n = 1705 ROIs), 1212 ± 236.4 (n = 1705 ROIs), 1316 ± 297.9 (n = 1705 ROIs), and 2148 ± 422.2 (n = 1705 ROIs) under umIF conditions. The crowding‐enhanced umIF buffer increased signal intensity at earlier time points for both markers. Fluorescence images within each target‐specific dataset were acquired using identical imaging settings. Scale bars, 200 µm (main panels); 50 µm (insets). The corresponding fluorescence intensity from the labeled targets, together with the contrast‐to‐noise ratio (CNR), are provided in Tables S1 and S2.

Using dye‐conjugated primary antibodies (1°Ab) in human colon tissue, we examined CD19, PCNA and E‐cadherin under conventional and umIF buffer conditions (Figure 2 for CD19 and PCNA, and Figure S4 for E‐cadherin). Across all three targets, the crowding‐enhanced buffer increased signal accumulation during incubation, with improvement already evident at early time points. As shown in Figure 2A, this effect was especially pronounced for the weak target CD19, which remained relatively weak under conventional conditions even after 24 h of incubation, but became readily detectable under umIF conditions after substantially shorter incubation of just 30 min (Tables S1 and S2). For PCNA (Figure 2B) and E‐cadherin (Figure S4, Tables S3), umIF likewise increased signal during the accumulation phase, although the magnitude and temporal profile of the benefit varied across targets. In addition, the observed staining patterns were consistent with expected spatial distributions, with CD19 enriched in inflamed regions of human colon tissue and PCNA concentrated in proliferative crypt regions of mouse intestine. Together, these results indicate that the umIF buffer improves effective labeling efficiency during staining and can provide a practically meaningful advantage for weak‐labeling targets.

We next asked whether a similar signal enhancement was observed for antibody‐nanobody complexes (1°Ab + 2°Nb, hereafter referred to as *complexes*). In mouse small intestine, umIF increased signal accumulation for both PCNA and E‐cadherin complexes relative to conventional conditions (Figures S5 and S6, Tables S4 and S5). For antibody‐nanobody complexes, the enhancement under umIF appeared earlier and was more pronounced for E‐cadherin than for PCNA, particularly at short incubation times. Thus, although the magnitude of the enhancement depends on both target class and labeling format, the overall trend was consistent: the umIF buffer improved signal accumulation during incubation.

To further assess whether the crowding agent primarily affects post‐labeling retention, we monitored signal decay over time after 24 h of incubation (Figures S7–S9, Tables S6–S9). Although the signal decreased over time, the decay profiles were broadly similar in the presence and absence of crowding agents. These results do not support reduced post‐labeling dissociation as the primary contributor to the improved signal under the conditions tested here. Rather, the most consistent effect of umIF was faster signal accumulation and higher early signal and contrast during the accumulation phase, whereas benefits in retention were more variable and depended on the target and labeling format.

### 2.3 umIF Improves Labeling Efficiency of One‐Step Same‐Species Antibody‐Nanobody Complexes

**FIGURE 3:**
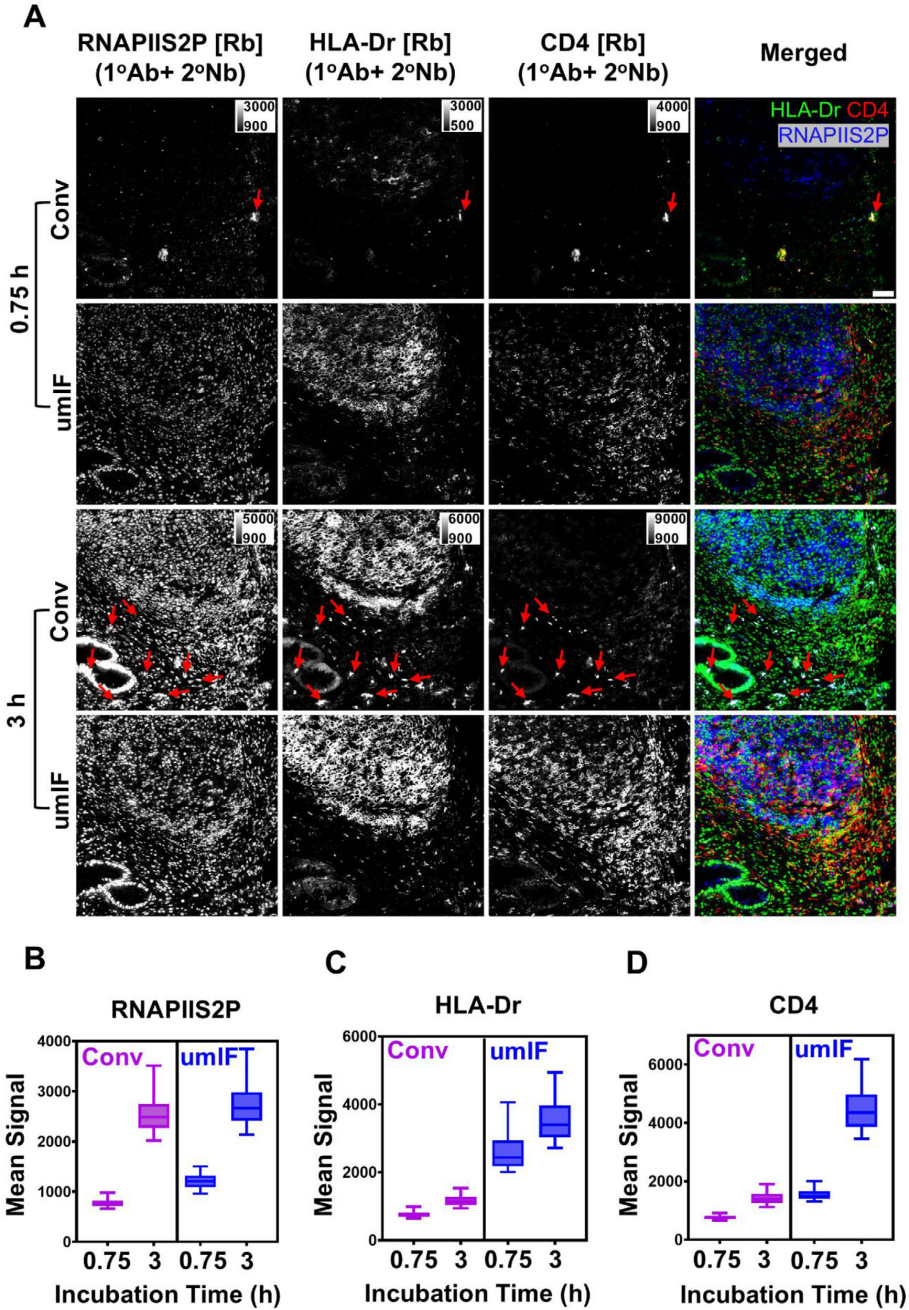
umIF improves labeling efficiency of antibody‐nanobody complexes (1°Ab + 2°Nb) compared with conventional immunofluorescence. (A) Representative images of human inflamed colon tissue co‐stained with the same‐host rabbit‐derived nanobody complexes targeting RNAPII‐S2P (blue), HLA‐DR (green), and CD4 (red) using conventional labeling (conv) or umIF labeling for 45 min or 2 h. Conventional labeling (conv) produced weaker and more aggregates (red arrows), whereas umIF yielded brighter and more homogeneous staining across all targets. The number of aggregates on the whole‐slide images (1.8 mm x 2.6 mm) is approximately 145 and 35 aggregated spots in conventional and umIF approaches, respectively. Merged panels illustrate simultaneous visualization of transcriptional, antigen‐presenting, and T‐cell compartments specifically using the same host nanobody complexes (1°Ab + 2°Nb). (B–D) Quantification of mean fluorescence intensity for RNAPII‐S2P (B), HLA‐DR (C), and CD4 (D) as shown in (A), presented as box‐and‐whisker plots (5th–95th percentile). For RNAPII‐S2P, fluorescence intensities (mean ± SD, a.u.) were 780.7 ± 108.9 (n = 5249 ROIs) and 2581.0 ± 505.9 (n = 3404 ROIs) under conventional conditions at 0.75 and 3 h, respectively, and 1212.0 ± 168.6 (n = 3984 ROIs) and 2762.0 ± 519.7 (n = 3385 ROIs) under umIF conditions. For HLA‐DR, fluorescence intensities (mean ± SD, a.u.) were 769.8 ± 134.0 (n = 1449 ROIs) and 2661.0 ± 695.6 (n = 1877 ROIs) under conventional conditions at 0.75 and 3 h, respectively, and 1177 ± 209.5 (n = 2640 ROIs) and 3578.0 ± 752.5 (n = 2137 ROIs) under umIF conditions. For CD4, fluorescence intensities (mean ± SD, a.u.) were 762.9 ± 77.9 (n = 2590 ROIs) and 1566 ± 271.7 (n = 2441 ROIs) under conventional conditions at 0.75 h and 3 h, respectively, and 1431.0 ± 249.7 (n = 2747 ROIs) and 4517.0 ± 870.9 (n = 2616 ROIs) under umIF conditions. umIF produced significantly higher intensities than conventional labeling at both 0.75 and 3 h incubation time points. Fluorescence images within each target‐specific dataset were acquired using identical imaging settings. Scale bars, 50 µm. Corresponding fluorescence intensity and contrast‐to‐noise ratio (CNR) values are provided in Table S10.

Because the time‐course studies above indicated that umIF buffer primarily improves signal accumulation during staining, we next examined how this effect translates to simultaneous one‐step co‐incubation of multiple same‐species antibody‐nanobody complexes. Although premixed strategies based on *complexes* formed between unconjugated 1°Ab and fluorophore‐labeled 2°Nb have been explored previously, their widespread adoption has been limited due to suboptimal and inconsistent performance [7, 19, 20]. To examine this limitation directly, we tested human colon tissue with active ulcerative colitis. As shown in Figure 3, conventional one‐step co‐incubation of three rabbit‐derived complexes including RNAPII‐S2P (nuclear), HLA‐DR (surface), and CD4 (surface), in which each unconjugated 1°Ab from the same host species was separately premixed with a spectrally distinct dye‐conjugated 2°Nb prior to co‐incubation with the sample, resulted in reduced labeling efficiency and visible aggregate‐like signals. These artifacts are visible as white dots in the merged images and are marked by red arrows in both grayscale and merged views. Under conventional conditions, the staining was weak after 30 min co‐incubation; extending to 2 h modestly improved RNAPII‐S2P and HLA‐DR signals but not CD4, while markedly increasing aggregate formation. Together, these results illustrate the reduced labeling efficiency and uneven target performance when simultaneous multiple same‐species antibody‐nanobody complexes are co‐incubated under conventional conditions.

In contrast, umIF employing same‐species complexes markedly improves labeling performance. As demonstrated in Figure 3, the one‐step co‐incubation of multiple same‐species complexes using the optimized umIF buffer produced stronger fluorescence signals across diverse targets after both 45 min and 3 h of incubation. Three rabbit‐derived complexes targeting RNAPII‐S2P, HLA‐DR, and CD4 were reliably detected in a single co‐incubation step using umIF complexes. Similar improvement was also observed across different incubation times and tissue types, including 3 h or 24 h in mouse intestine (Figure S10) and 30 min in mouse lung tissue (Figure S11). As shown in Figure 3B–D, Figures S10B, C and S11B, C, the umIF complexes consistently produced stronger signals across both nuclear and surface markers. Furthermore, the method is scalable, enabling simultaneous detection of five or more targets in a single cycle, limited only by the imaging system (Figure S12). In addition, within this range, mixed monoclonal and polyclonal primary antibodies (Tables S17–S20) were compatible. Extension to substantially larger simultaneously co‐incubated panels will require dedicated future investigation.

In previous implementations of labeling multiple targets with host‐independent complexes, a sequential incubation strategy has been used, in which each *complex* is applied one at a time to its target to minimize potential cross‐reactivity [7]. To directly compare the two approaches, we evaluated our umIF workflow, where three *complexes* were co‐incubated simultaneously in a single step, against the sequential incubation strategy of one complex at a time, requiring three incubation steps with washes in between. As shown in Figure S13, the one‐step co‐incubation of three *complexes* targeting cytokeratin, CD3 and RNAPII‐S2P produced stronger signals than sequential staining. The reduced signal observed with the sequential protocol may result from dissociation of secondary nanobodies from *complexes* during the extended staining procedure, where repeated wash steps without post‐fixation [7, 19] likely promoted complex instability. Together, these results demonstrate that simultaneous co‐incubation under umIF conditions improves the labeling efficiency and complex stability, while simplifying the workflow for multiplex immunofluorescence labeling.

To further assess staining fidelity, we compared labeling obtained with a dye‐conjugated primary antibody and an antibody‐nanobody complex directed against the same target as an internal positive control. The two channels showed colocalization under both conventional and umIF conditions (Figure S14), with Pearson correlation coefficients of 0.65 and 0.85, respectively, indicating that the complex format reproduced the expected spatial labeling pattern and that umIF improved the fidelity of this labeling under the tested conditions. In addition, negative‐control experiments performed without primary antibody showed minimal nonspecific labeling (Figure S15). Together, these controls support the pattern fidelity and low background of the umIF complex staining strategy under the tested conditions.

### 2.4 Comparison of Nanobody‐Based umIF Workflows with Conventional Two‐Step Indirect IF

Having established that umIF enables robust same‐species one‐step complex staining, we next compared nanobody‐based umIF workflows with conventional two‐step indirect IF, which remains the gold standard for signal amplification in multiplexed immunofluorescence. Specifically, we evaluated three labeling schemes under matched staining and imaging conditions, including conventional two‐step IgG staining (1°Ab → 2°Ab), two‐step nanobody staining (1°Ab → 2°Nb), and one‐step premixed antibody‐nanobody complexes (1°Ab + 2°Nb), each under conventional and umIF buffer conditions.

**FIGURE 4:**
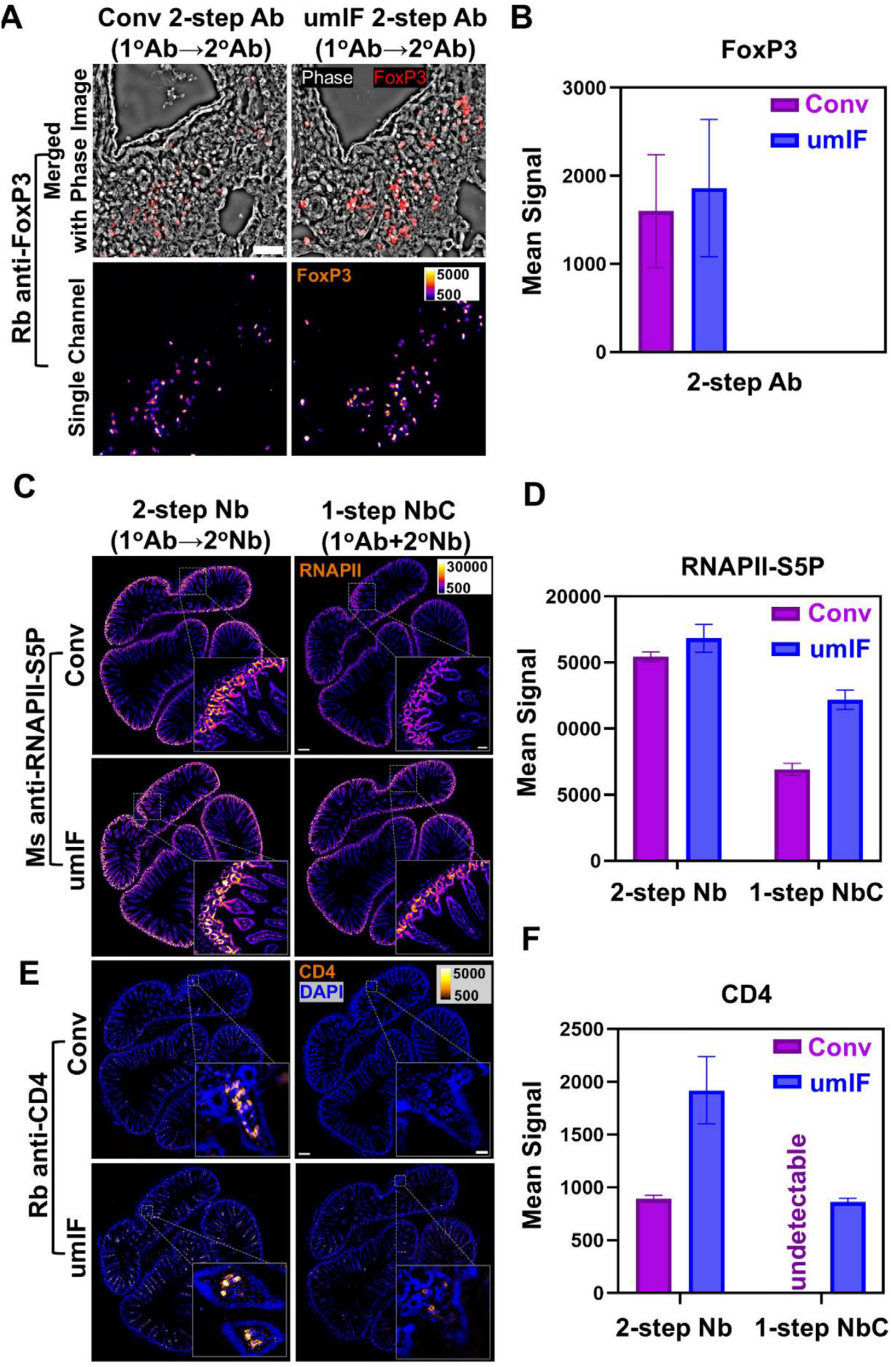
umIF enhances labeling performance across two‐step and one‐step immunofluorescence formats. (A) Representative images of mouse lung tumor tissue stained with rabbit anti‐FoxP3 using conventional or umIF two‐step IgG labeling (1°Ab → 2°Ab) with 2 h of incubation. Top: merged FoxP3 (red) with quantitative phase images show clearer nuclear localization with umIF. Bottom: single‐channel FoxP3, showing slightly enhanced labeling efficiency with umIF compared with the conventional method. Scale bars, 50 µm. (B) Quantification of FoxP3 fluorescence intensity corresponding to (A), presented as box‐and‐whisker plots (5th–95th percentile). Fluorescence intensities (mean ± SD, a.u.) were 1869 ± 845.1 (n = 776 ROIs) and 1996 ± 821.2 (n = 804 ROIs) under conventional and umIF two‐step labeling conditions, respectively. Comparable labeling performance was observed between conventional and umIF two‐step labeling conditions. (C, E) Representative images of mouse intestine tissue stained for RNAPII‐S5P and CD4 under matched staining conditions using conventional two‐step nanobody staining (1°Ab → 2°Nb), and one‐step premixed antibody‐nanobody complexes (1°Ab + 2°Nb), each under conventional or umIF buffer conditions. Insets show enlarged views of the boxed regions. (D, F) Quantification of fluorescence intensity for RNAPII‐S5P (D) and CD4 (F) corresponding to (C) and (E) between two‐step Nb and one‐step complexes, presented as box‐and‐whisker plots (5th–95th percentile) between conventional and umIF conditions. For RNAPII‐S5P, fluorescence intensities (mean ± SD, a.u.) were 15447.5 ± 366.6 and 16833.4 ± 1071.4 for conventional and umIF two‐step nanobody staining (1°Ab → 2°Nb), respectively, and 6935.9 ± 451.2 and 12176.7 ± 723.8 for conventional and umIF one‐step antibody‐nanobody complexes (1°Ab + 2°Nb), respectively. For CD4, fluorescence intensities (mean ± SD, a.u.) were 891.8 ± 34.5 and 2058.1 ± 193.5 for conventional and umIF two‐step nanobody staining (1°Ab → 2°Nb), respectively. CD4 signal was undetectable under conventional one‐step antibody‐nanobody complex staining (1°Ab + 2°Nb), whereas fluorescence intensity under umIF conditions was 864.1 ± 31.8 (mean ± SD, a.u.). n = 3 analyzed image regions per condition for RNAPII‐S5P analysis; n = 4, 3, and 3 analyzed image regions for conventional 2‐step Nb, umIF 2‐step Nb, and umIF 1‐step NbC conditions, respectively, for CD4 analysis. Fluorescence images within each target‐specific dataset were acquired using identical imaging settings. Corresponding fluorescence intensity and contrast‐to‐noise ratio (CNR) values are provided in Tables S11–S13.

As shown in Figure 4, the umIF buffer generally improved labeling efficiency across all tested formats. For the surface marker CD4, two‐step staining produced a stronger overall signal, while umIF enhanced labeling across all three formats and enabled robust detection in the one‐step complex format. For the nuclear marker RNAPII‐S5P, the two‐step formats remained stronger overall than the one‐step complex format, although umIF improved complex‐based staining for both RNAPII‐S5P and CD4, with the gain being especially important for enabling robust detection of CD4. These results indicate that one‐step umIF complexes do not generally exceed the signal obtained with two‐step indirect IF. Rather, the main advantage of the complex format is that it enables host‐independent co‐incubation of same‐species antibodies in a simplified one‐step workflow, which is particularly valuable for expanding multiplexing flexibility, whereas the two‐step formats remain preferable when maximal signal intensity is the primary consideration.

### 2.5 umIF Integrates with An Established Multi‐Cycle High‐Plex Imaging Workflow

Having established the performance of umIF in single‐cycle staining format, we next evaluated whether umIF is compatible with iterative hyper‐plex workflows that require repeated rounds of staining, imaging, and antibody removal. We implemented umIF within the well‐established iterative indirect immunofluorescence imaging (4i) framework [3, 21], using the corresponding cyclic imaging workflow and elution strategy as the underlying platform, which has previously enabled high‐dimensional protein measurements across many cycles.

**FIGURE 5:**
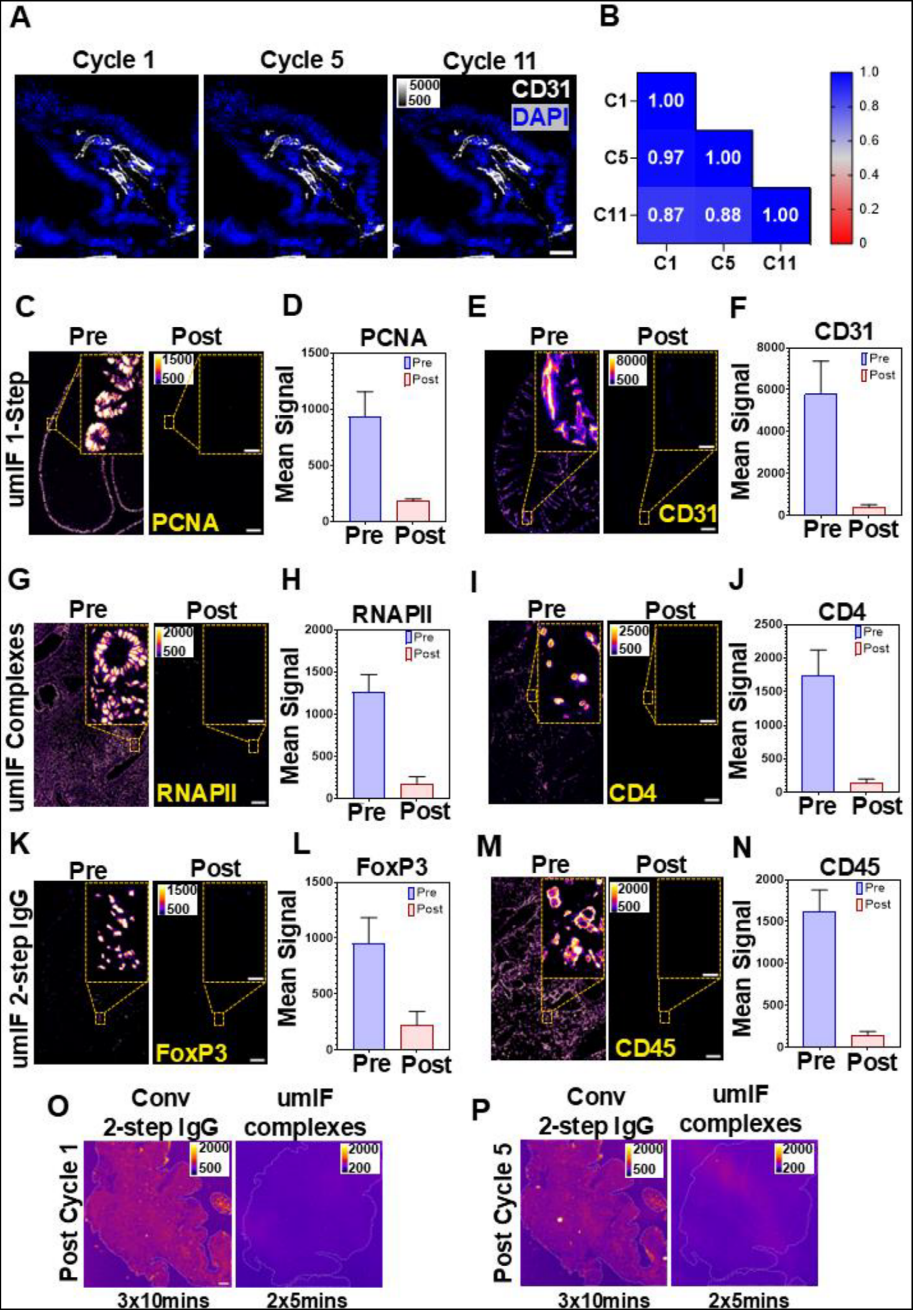
umIF preserves structural fidelity across cycles and supports efficient elution across staining formats in multicycle imaging. (A) Representative images of the same CD31‐stained structure overlaid with DAPI from mouse intestinal tissue acquired at Cycle 1, Cycle 5, and Cycle 11. (B) Pearson correlation matrix of the images across cycles. Pearson correlation coefficients were 0.9704 between Cycle 1 and Cycle 5, 0.8681 between Cycle 1 and Cycle 11, and 0.8809 between Cycle 5 and Cycle 11 (*p* < 0.0001 for all comparisons; n = 2010 paired intensity values per comparison). Mean fluorescence intensities (mean ± SD, a.u.) were 9.931 ± 1.072 for Cycle 1, 9.959 ± 1.055 for Cycle 5, and 10.090 ± 0.689 for Cycle 11. (C, E) Representative images of PCNA (C) and CD31 (E) obtained using dye‐conjugated antibodies (1‐step 1°Ab) before (pre‐elution) and after (post‐elution) signal removal. Insets indicate regions used for magnification. (D, F) Quantification of mean fluorescence intensity corresponding to (C) and (E). Fluorescence intensities (mean ± SD, a.u.) were 935.2 ± 220.8 and 181.6 ± 21.1 for PCNA before (pre‐elution) and after (post‐elution) signal removal, respectively (n = 540 ROIs per condition), and 5789 ± 1568 and 379.8 ± 149.8 for CD31 before and after signal removal, respectively (n = 2402 ROIs per condition). (G, I) Representative images of RNAPII (G) and CD4 (I) obtained using nanobody complexes (1°Ab + 2°Nb) before and after elution. Fluorescence intensities (mean ± SD, a.u.) were 1259 ± 209.0 and 175.8 ± 84.90 for RNAPII before and after signal removal, respectively (n = 8083 ROIs per condition), and 1747 ± 370.2 and 136.6 ± 63.51 for CD4 before and after signal removal, respectively (n = 819 ROIs per condition). (H, J) Quantification of mean fluorescence intensity corresponding to (G) and (I). (K, M) Representative images of FoxP3 (K) and CD45 (M) obtained using two‐step IgG (1°Ab → 2°Ab) staining before and after elution. Insets indicate regions used for magnification. (L, N) Quantification of mean fluorescence intensity corresponding to (K) and (M). Fluorescence intensities (mean ± SD, a.u.) were 955.2 ± 226.5 and 224.6 ± 122.6 for FoxP3 before and after signal removal, respectively (n = 263 ROIs per condition), and 1620.0 ± 258.1 and 151.7 ± 40.4 for CD45 before and after signal removal, respectively (n = 2080 ROIs per condition). (O, P) Representative images comparing conventional two‐step IgG staining and umIF nanobody‐complex staining after repeated cycles (Cycle 1 and Cycle 5) following format‐specific elution conditions. Conventional two‐step IgG staining was eluted for three 10‐min washes, whereas one‐step umIF nanobody‐complex staining was eluted for two 5‐min washes. For all panels, insets show magnified regions of interest. Fluorescence images within each target‐specific dataset were acquired using identical imaging settings. For pre‐ and post‐elution comparisons, fluorescence images were spatially registered and quantified using the same signal mask generated from the corresponding pre‐elution image. Scale bars: 200 µm for overview and 50 µm for insets.

To assess its performance across multiple staining‐elution cycles, we first applied umIF complexes across repeated rounds of multiplexed IF. To evaluate preservation of structural fidelity across repeated cycling, we compared staining of CD31 in Cycle 1, Cycle 5, and Cycle 11. As shown in Figure 5A, B, the spatial labeling pattern was well preserved across cycles, with a high degree of similarity in the corresponding Pearson correlation matrix (0.87 between Cycle 1 and Cycle 11). These results support that the established 4i‐based cyclic workflow remains compatible with umIF under the conditions tested, while preserving interpretable spatial labeling patterns through at least 11 cycles.

We next assessed elution efficiency across three labeling strategies within the same multicycle framework, including direct one‐step staining with dye‐conjugated primary antibodies (1°Ab), umIF complexes (1°Ab + 2°Nb), and umIF 2‐step IgG staining (1°Ab → 2°Ab). As shown in Figure 5C–N, representative pre‐ and post‐elution images and their corresponding quantification demonstrated effective reduction of fluorescence signal across all three formats, including PCNA and CD31 for direct 1‐step staining, RNAPII and CD4 for umIF complexes, and FoxP3 and CD45 for umIF 2‐step IgG staining. These results indicate efficient antibody removal between cycles across diverse labeling modes.

To further compare post‐elution background after repeated cycling, we examined conventional 2‐step IF [3, 6] and umIF complexes after multiple rounds of staining and elution. As shown in Figure 5O, P, umIF complexes exhibited lower residual background and cleaner signal removal under the tested conditions, even though their elution was performed with a shorter and less intensive protocol than conventional 2‐step IgG IF (two 5‐min washes vs. three 10‐min washes). Together, these results show that umIF can be integrated into an established 4i‐based cyclic IF workflow while preserving structural fidelity across repeated cycles and supporting efficient elution across multiple staining formats. These results indicate that umIF can be integrated into an established 4i‐based cyclic IF workflow while preserving structural fidelity across repeated cycles, supporting efficient elution across multiple staining formats and flexible use of both one‐step and two‐step labeling strategies for high‐plex spatial proteomics workflows.

### 2.6 umIF‐Based High‐Plex Spatial Profiling Of Human Pathological Tissues

**FIGURE 6:**
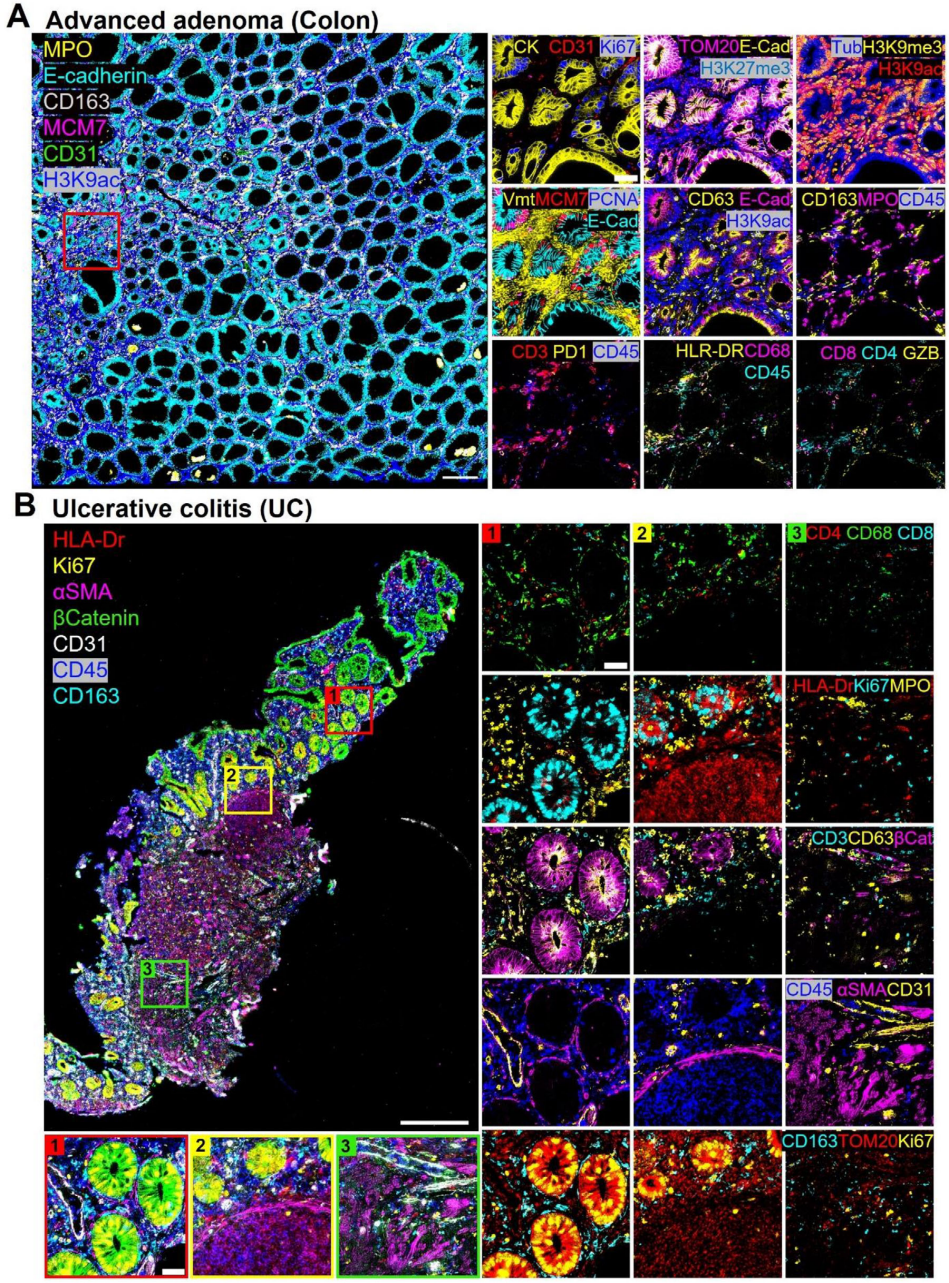
Multi‐cycle umIF profiling reveals spatial organization of epithelial, stromal, and immune cell populations in human colon tissues. (A) Representative umIF images of a colon polyp from a patient with advanced adenoma processed through 10 iterative umIF cycles (25 of 27 total targets shown) with a panel of epithelial, cytoplasmic, stromal, and immune markers, including TOM20, CD63, MPO, E‐cadherin, Cytokeratin, Vimentin (Vmt), CD31, αSMA, Ki67, MCM7, Fox‐P3, H3K27me3, H3K4me3, H3K9me3, H3K9ac, PCNA, HLA‐DR, CD45, CD3, CD4, CD8, CD68, CD163, PD1, and Granzyme B (GZB). Enlarged view (red box) shows epithelial proliferation, histone modification, and immune cell infiltration. Scale bars: 200 µm (overview), 50 µm (insets). (B) Representative umIF images of an inflamed colon tissue (ulcerative colitis (UC)) stained through 7 umIF cycles (13 of 15 total targets shown) with a multiplex panel including HLA‐DR, Ki67, β‐catenin, αSMA, CD31, CD3, CD4, CD8, CD45, CD68, CD163, MPO, and TOM20. Enlarged insets (regions 1–3) display spatially distinct epithelial, stromal, and immune cell populations in (1) normal‐appearing epithelial area, (2) border between normal epithelium and inflamed region and (3) inflamed region. Scale bars, 500 µm (overview) and 50 µm (insets).

To assess the translational utility of umIF, we next applied it to clinically relevant human FFPE specimens, including advanced adenomas of the colon and ulcerative colitis. In Figure 6A, we applied a 27‐marker panel including epithelial, stromal, vascular, mitochondrial, immune, and chromatin markers (Table S16), combining antibody‐nanobody complexes (1°Ab + 2°Nb) with dye‐conjugated primaries (1°Ab), to an advanced adenoma (sessile serrated adenoma) FFPE tissue section. Corresponding single‐channel images are shown in Figure S16. Epithelial markers (E‐cadherin, cytokeratin) revealed the serrated glandular architecture, while the surrounding stroma contained abundant CD45^+^ immune infiltrates dominated by CD68^+^CD163^+^ macrophages, accompanied by MPO^+^ neutrophils and moderate CD4^+^ T cells. A subset of CD4^+^ T cells expressed Granzyme B, whereas CD8^+^ and PD1^+^ cells were sparse, reflecting limited cytotoxic activity. Proliferation markers (Ki67, MCM7, and PCNA) exhibited heterogeneous expression across glands and mitochondrial TOM20 and chromatin (H3K9ac, H3K27me3) markers are consistent with metabolic and epigenetic variability between epithelial glands. The CD63^+^ extracellular vesicle‐associated cells appeared along the luminal borders of epithelial glands and dispersed throughout the stroma, suggesting possible epithelial‐stromal communication. These spatial features reveal a proliferative microenvironment enriched for immunosuppressive macrophages with limited cytotoxic activity, consistent with an immune‐tolerant environment in sessile serrated adenomas.

Figure 6B presents a 15‐marker umIF panel (Table S17) applied to colon tissue from a patient with ulcerative colitis, showing three distinct regions: (1) normal‐appearing mucosa, (2) a mixed normal‐inflamed interface, and (3) inflamed mucosa. Single‐channel images for each marker in these regions are shown in Figures S17–S19. In the normal region (region 1), epithelial architecture was preserved, with strong β‐catenin expression and limited immune infiltration. At the mixed interface (region 2), αSMA^+^ smooth muscle delineated the boundary between normal and inflamed tissue, where dense CD45^+^ infiltrates exhibited high HLA‐DR expression, indicative of active immune activation. The inflamed region (region 3) displayed stromal remodeling with αSMA^+^ fibroblasts, CD31^+^ vasculature, and diverse immune populations including CD68^+^ and CD163^+^ macrophages, CD4^+^ and CD8^+^ T cells, and MPO^+^ neutrophils. Increased Ki67^+^ epithelial proliferation and altered β‐catenin expression further reflect disrupted tissue architecture characteristic of inflammation.

Together, these results demonstrate that umIF can be applied to archived patient FFPE specimens, enabling high‐plex, spatially‐resolved protein profiling. By flexibly integrating antibody‐nanobody complexes (1°Ab + 2°Nb) and dye‐conjugated primaries according to antibody availability, the umIF workflow provides a practical and flexible approach for large‐scale tissue profiling in clinical and translational studies.

### 2.7 umIF‐Based High‐Plex Spatial Profiling of Mouse Tissues

**FIGURE 7:**
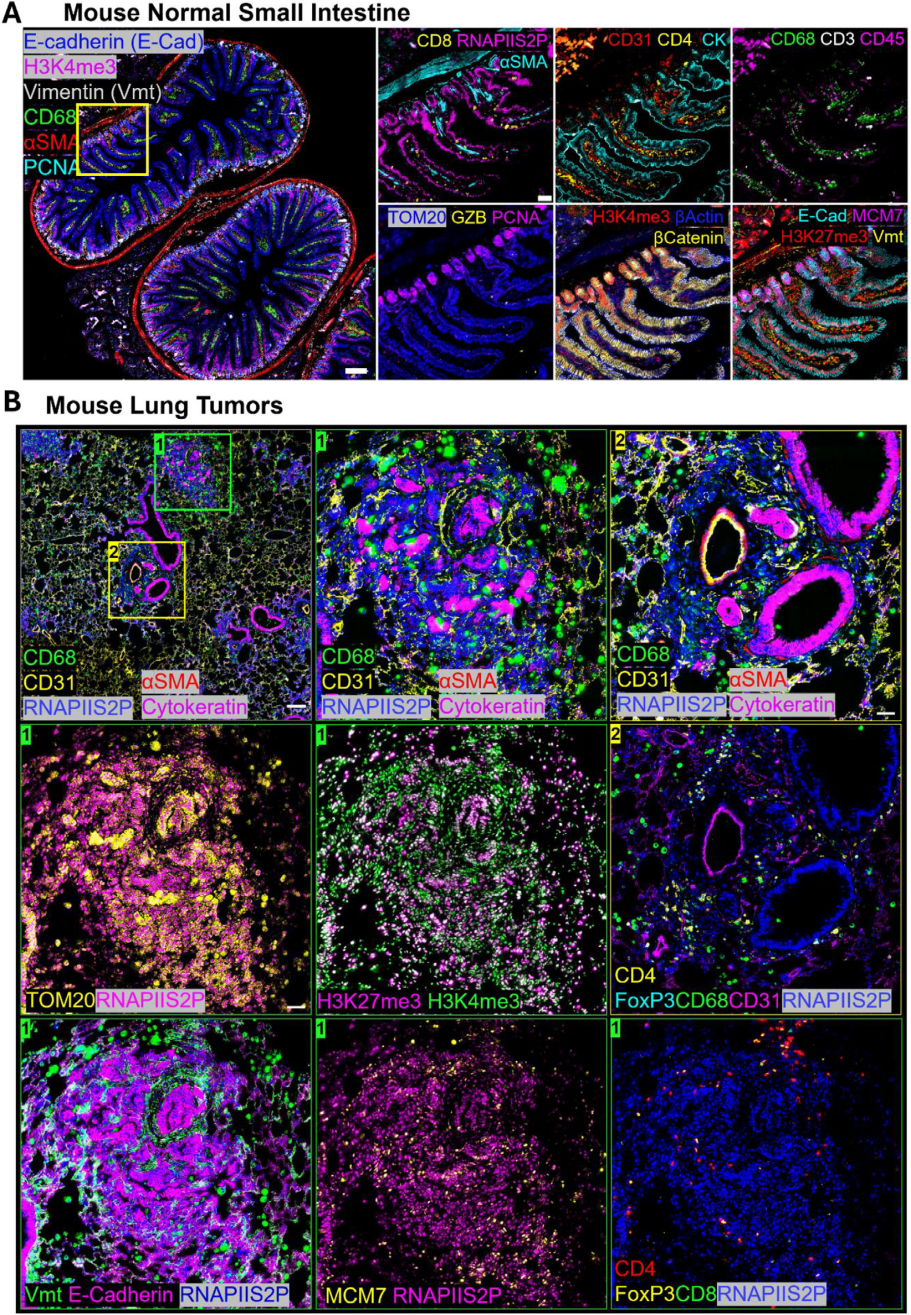
High‐plex umIF profiling of mouse models, mapping epithelial, stromal, and immune landscape in normal intestine and lung cancer. (A) Representative umIF images of mouse small intestine stained through 10 umIF cycles (19 of 26 total targets shown) with a panel of epithelial, stromal, nuclear/histone modifications, and immune markers, including E‐cadherin (E‐Cad), cytokeratin (CK), β‐catenin (βCat), PCNA, MCM7, vimentin (Vmt), αSMA, CD31, TOM20, RNAPII‐S2P, H3K27me3, H3K4me3, H3K9me3, CD3, CD4, CD45, CD68, PD1, and Granzyme B (GZB). The enlarged inset (yellow box) highlights spatial organization of epithelium and stroma with distinct nuclear, cytoplasmic, and membrane signals. (B) Representative umIF images of *Kras* ^G12D^ *Lkb1* ^−/−^ mouse model of non‐small cell lung cancer (NSCLC), stained through 10 iterative umIF cycles (16 of 28 total targets shown) with a panel of markers, including CK, E‐Cad, CD31, Vmt, FoxP3, MCM7, RNAPII‐S2P, H3K27me3, H3K4me3, TOM20, CD4, CD8, and CD68. Insets (regions 1–2) illustrate tumor, stroma, proliferation, mitochondrial markers, and immune infiltrates. Enlarged views emphasize spatial heterogeneity of the tumor microenvironment. Scale bars, 200 µm (overview) and 50 µm (insets).

Building on the demonstration of umIF in human FFPE tissues, we next applied the umIF platform to mouse models to evaluate cross‐species performance. A 26‐marker panel (Table S18) was applied to normal mouse small intestine (Figure 7A), and a 31‐marker panel (Table S19) was applied to spatial mapping of tumor microenvironment in a *Kras* ^G12D^ *Lkb1* ^−/−^ (KL) mouse model of non‐small cell lung cancer (NSCLC) [22] (Figure 7B).

In the normal intestine, umIF delineated epithelial structure, stroma, vasculature, proliferation activity, immune cell distribution, and chromatin states within the same tissue section. The corresponding single‐channel images for each marker are shown in Figure S20. At the bottom of the crypts (Figure 7A), the proliferative intestinal stem cells showed high levels of PCNA and MCM7, accompanied by enrichment of the active chromatin mark H3K4me3 and reduced repressive mark H3K27me3. Within the lamina propria, CD68^+^ macrophages and CD4^+^ T cells were prominently localized, including perivascular regions, reflecting their established roles in intestinal immune surveillance and tissue homeostasis.

In the KL model, umIF‐based spatial mapping revealed a heterogeneous microenvironment characteristic of *Kras* ^G12D^ *Lkb1* ^−/−^‐driven lung tumors (Figure 7B, with single‐channel images for each region shown in Figure S21). Region 1 corresponds to a tumor focus enriched in the active form of RNA polymerase II (RNAPII‐S2P), indicating high transcriptional activity. This region contains densely packed epithelial cell clusters with strong expression of E‐cadherin and pan‐cytokeratin, along with abundant mitochondrial marker TOM20 and elevated level of active histone mark H3K4me3, consistent with metabolically and transcriptionally active tumor epithelium. In addition, an extensive stromal (vimentin^+^) and microvasculature (CD31^+^) network is evident, interconnected with the epithelial tumor regions.

Region 2 highlights a perivascular region enriched in CD31^+^ endothelial cells and αSMA^+^ vascular smooth muscle cells, outlining well‐developed vasculature. This region contains scattered immune cells such as macrophages (CD68^+^), T helper cells (CD4^+^) and FoxP3^+^ regulatory T cells (CD4^+^/FoxP3^+^). Notably, the active form of RNA polymerase II (RNAPII‐S2P) is also enriched in this area, suggesting increased transcriptional activity within the perivascular compartment among stromal and immune populations. However, CD4^+^ and CD8^+^ T cells are overall sparse, consistent with the “immune‐cold” phenotype of KL tumors [23].

Together, these results further illustrate the capability of umIF for comprehensive spatial profiling of epithelial, stromal, vascular, immune, and chromatin features in both normal and diseased mouse tissues. This broad applicability across organ systems and pathologies underscores the potential of umIF to provide mechanistic insights in preclinical models.

### 2.8 Application of umIF to Profile Epigenetic States Across Diverse Immune Cell Populations in the KL Mouse Model

**FIGURE 8:**
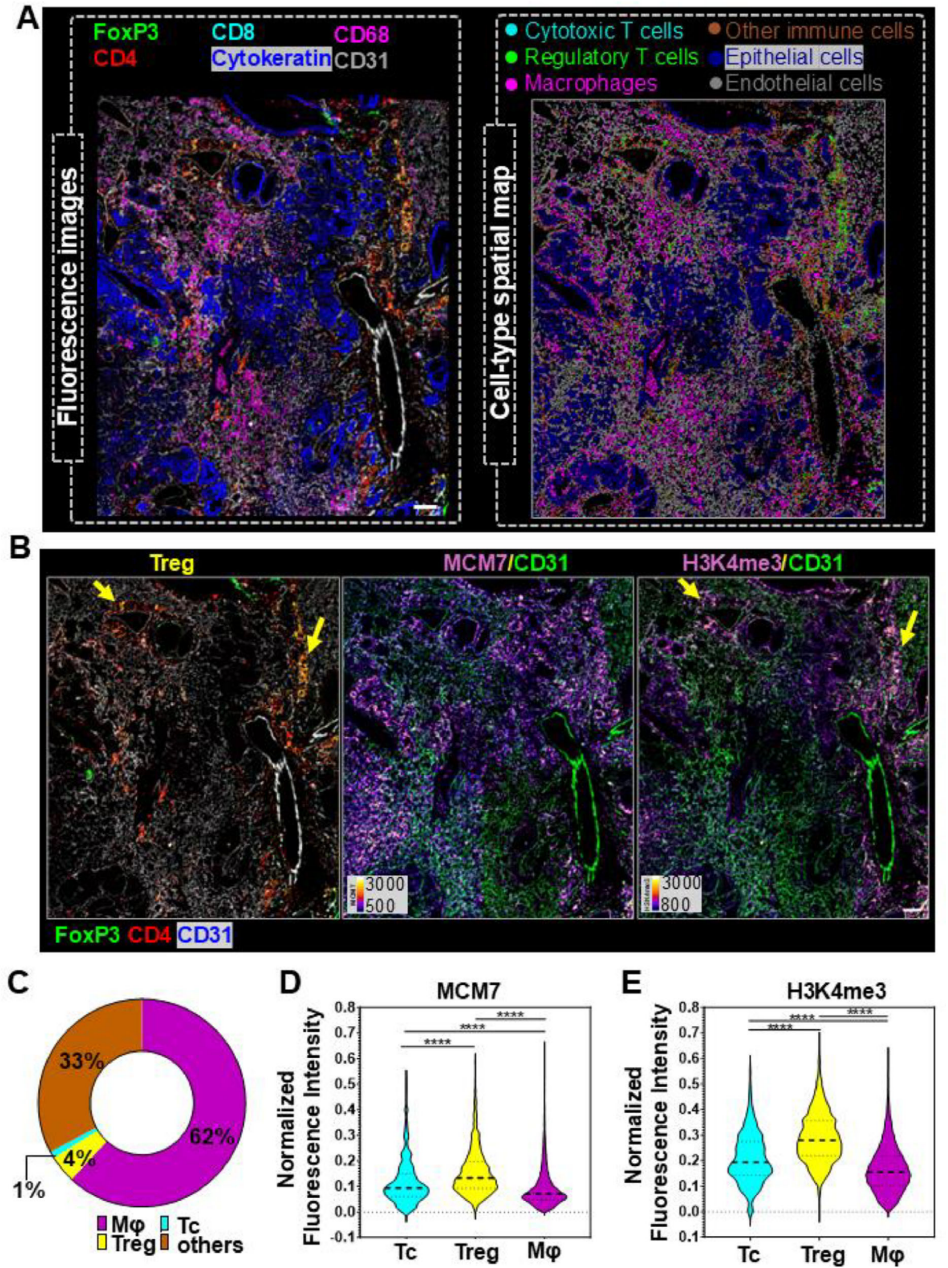
Spatial and functional characterization of immune and stromal populations in a tissue section from *Kras* ^G12D^ *Lkb1* ^−/−^ mouse model. (A) Representative multiplexed fluorescence image (left) and corresponding cell‐type spatial map (right) of *Kras* ^G12D^ *Lkb1* ^−/−^ mouse tissue stained for CD68 (macrophages), CD8 (cytotoxic T cells), CD4 and FoxP3 (regulatory T cells), CD31 (endothelial cells), and Cytokeratin (epithelial cells). Distinct immune and stromal populations were computationally segmented and classified into cytotoxic T cells (Tc), regulatory T cells (Treg), macrophages (Mφ), endothelial, and epithelial cells. (B) Representative fluorescence images showing the spatial distribution of CD4 (red) and FoxP3 (green) within tumor microenvironment (left), together with corresponding expression maps for proliferation (MCM7, middle) and active chromatin state (H3K4me3, right) markers. CD31 (gray/green) was included as a structural landmark for microvasculature. (C), Quantification of immune cell composition (n = 38 505 cells) showing macrophages (62%), cytotoxic T cells (1%), Tregs (4%), and other immune cells (33%). (D‐E), Violin plots showing normalized fluorescence intensity of MCM7 (D) and H3K4me3 (E) across Tc, Treg, and macrophages populations. For MCM7, normalized fluorescence intensities (mean ± SD) were 0.1163 ± 0.0857 for Tc (n = 387 cells), 0.1545 ± 0.0881 for Treg (n = 1660 cells), and 0.0923 ± 0.0736 for macrophages (n = 23827 cells). For H3K4me3, normalized fluorescence intensities (mean ± SD) were 0.2088 ± 0.0974 for Tc (n = 387 cells), 0.2900 ± 0.0989 for Treg (n = 1660 cells), and 0.1662 ± 0.0898 for macrophages (n = 23827 cells). Each point represents an individual cell measurement; dashed lines indicate median values. Statistical significance was determined using one‐way ANOVA followed by Tukey's multiple‐comparisons test. ^****^ *p* < 0.0001. Scale bars, 200 µm.

We applied umIF to map the spatial organization of diverse cell populations and their epigenetic states in the KL mouse model (Figure 8). Distinct cell populations including cytotoxic T cells (CD8^+^), regulatory T cells (CD4^+^FoxP3^+^), macrophages (CD68^+^), epithelial cells (CK^+^), and endothelial cells (CD31^+^), were segmented and mapped within the tumor microenvironment (Figure 8A and Figure S22). The spatial maps revealed a dense microvascular network (CD31^+^) accompanied by macrophage‐rich stromal regions, interspersed with scattered Tregs and few cytotoxic T cells [24, 25]. Quantitative cell profiling showed that macrophages dominated the immune compartment (62%), whereas Tregs and CD8^+^ T cells accounted for 4% and 1%, respectively (Figure 8C), indicating a macrophage‐rich tumor microenvironment. This finding again is consistent with an immune‐cold and macrophage‐dominant microenvironment, consistent with previous reports linking LKB1 loss to impaired T‐cell recruitment and myeloid‐driven immunosuppression [23].

We observed that the accumulation of CD4^+^ cells occurred in the stromal and perivascular regions. This spatial organization aligns with prior reports of immune exclusion and Treg enrichment in KL‐driven tumorigenesis [23, 26]. Both qualitative visualization and quantitative analysis showed significant heterogeneity in transcriptional activity and chromatin states among φ) (Figure 8B,C). Notably, Tregs exhibited higher levels of H3K4me3 than other major immune cell subtypes, as shown in the regions indicated by the yellow arrows in Figure 8B, reflecting their active chromatin states. The proliferative marker MCM7 is also enriched in Tregs, as well as the tumor regions, suggesting that Tregs possess proliferative potential. Together, these findings provide a proof‐of‐concept demonstration that umIF can resolve spatially associated differences in immune‐cell chromatin state and proliferation markers within the KL immune microenvironment. In this model, the imaging‐based observations are consistent with an immune‐suppressive microenvironment in which regulatory T cells exhibit a comparatively active and proliferative chromatin‐associated profile [27].

**FIGURE 9:**
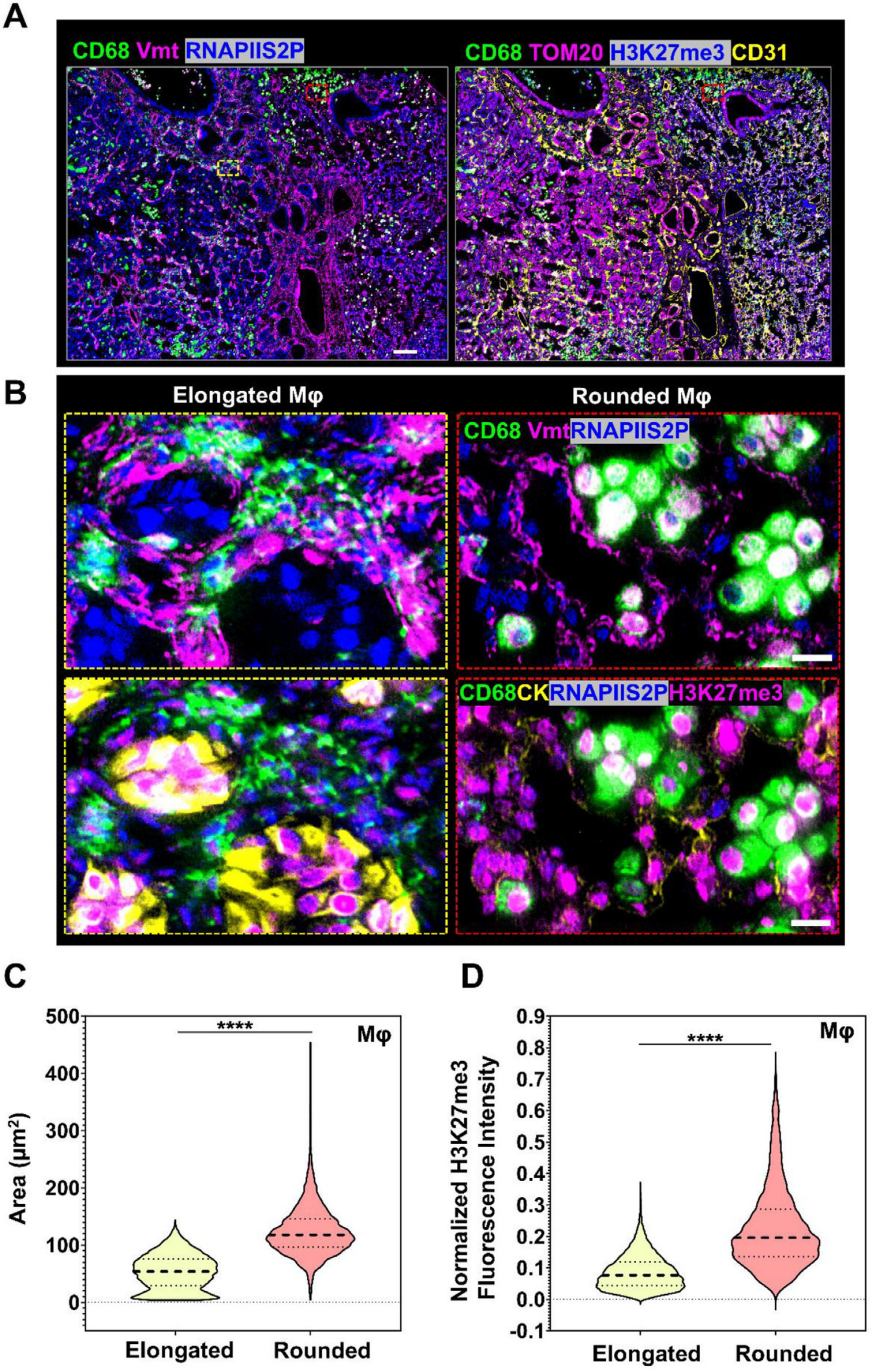
Morphological and functional heterogeneity of macrophages in lung tissue from *Kras* ^G12D^ *Lkb1* ^−/−^ mouse model. (A), Representative multiplexed fluorescence images of mouse lung tissue showing CD68^+^ macrophages (green) co‐stained with vimentin (Vmt, magenta) and RNAPII‐S2P (blue) (left), or with TOM20 (magenta), H3K27me3 (blue), and CD31 (yellow) (right). Scale bars, 200 µm. (B), Higher‐magnification views illustrating two distinct macrophage morphologies: elongated macrophages (left) and rounded macrophages (right). Insets show representative staining for CD68 (green), RNAPII‐S2P (blue), and Vmt (magenta), or for Cytokeratin (CK, yellow) and H3K27me3 (magenta). Scale bars, 20 µm. (C, D), Quantitative comparison of nuclear area (C) and normalized H3K27me3 fluorescence intensity (D) between elongated (n = 15 702 cells) and rounded (n = 8125 cells) macrophages (Mφ). For nuclear area, values (mean ± SD) were 53.78 ± 29.88 µm^2^ for elongated macrophages (n = 15 702 cells) and 124.3 ± 42.5 µm^2^ for rounded macrophages (n = 8125 cells). For normalized H3K27me3 fluorescence intensity, values (mean ± SD) were 0.0865 ± 0.0539 for elongated macrophages (n = 15 702 cells) and 0.2263 ± 0.1289 for rounded macrophages (n = 8125 cells). Data represent individual cell measurements; dashed lines indicate median values. Statistical significance was determined using an unpaired two‐tailed *t*‐test. ^****^ *p* < 0.0001.

Further spatial profiling revealed distinct morphological and epigenetic heterogeneity among macrophages, one of the central regulators of the tumor microenvironment in LKB1‐deficient lung tumorigenesis. Two predominant macrophage morphologies were observed: elongated and rounded (Figure 9). As shown in Figure 9A, B, elongated macrophages were frequently found within vimentin‐rich stromal regions and often positioned adjacent to cytokeratin^+^/TOM20^+^ tumor areas, suggesting a role in supporting tumor growth and stromal remodeling. In contrast, large, rounded macrophages were abundant in regions outside the epithelial tumor foci. Quantitative analysis of chromatin marks revealed that rounded macrophages, which exhibited a larger nuclear area, had significantly higher H3K27me3 intensity compared to their elongated counterparts (Figure 9B–D), indicating a more repressive chromatin state. These differences in macrophage chromatin states illustrate that umIF can detect spatially resolved morphological and chromatin‐state heterogeneity within macrophage populations, while suggesting potentially distinct functional states in the LKB1‐deficient lung tumor microenvironment [23, 28].

## 3 Discussion

This study presents umIF as a practical host‐independent immunofluorescence workflow that improves the flexibility and labeling efficiency of multiplexed IF in FFPE tissue. By coupling unconjugated primary antibodies with fluorophore‐conjugated secondary nanobodies in an optimized macromolecular crowding‐enhanced buffer, umIF enables simultaneous same‐species multiplexing within a single incubation step, while remaining compatible with multiple IF staining formats and iterative highly multiplexed imaging workflows. This design merges the flexibility of indirect IF with the host‐independence of direct IF and provides a practical route to same‐species multiplexing in settings where host‐species constraints and labeling efficiency limit conventional workflows.

Although antibody‐nanobody complexes have been previously proposed to bypass restriction in common IF, their performance has been compromised when multiple complexes are co‐incubated simultaneously. Consistent with prior studies from others [7, 9, 19], this limitation becomes particularly apparent in simultaneous one‐step co‐incubation, where reduced labeling efficiency and less consistent performance can limit practical adoption. In this context, our results suggest that the crowding‐enhanced umIF buffer improves the utility of the complex format primarily by improving effective labeling during staining.

In umIF, the key modification is the introduction of macromolecular crowding agents, which are known to modulate the local physical environment and enhance molecular interaction [13, 15, 16, 17, 29]. Our study provides imaging‐based evidence for improved staining performance in the presence of crowding agents such as dextran sulfate and Denhardt's solution without any chemical modification of antibodies or reagents. Our time‐course analyses indicate that, under the conditions tested here, the most consistent measurable effect of the crowding‐enhanced buffer is improved signal accumulation during the staining/incubation phase, especially for weak or difficult targets [9, 19].

The broader implication of this framework is that macromolecular crowding can serve as a broadly useful enhancer of immunolabeling efficiency, not just antibody‐nanobody‐based detection. We observed consistent signal improvement in both direct IF (dye‐conjugated primaries) and indirect IF (two‐step IgG or Nb‐based) in our extensive comparative studies, underscoring the benefits of crowding as a general enhancer of IF efficiency. At the same time, the benefit of umIF is context dependent. The effect was generally greatest when labeling efficiency was limited, whereas more strongly amplifying two‐step indirect formats could still provide higher signal for some targets. umIF is therefore best viewed as a flexible workflow that expands experimental options for efficient host‐independent detection of multiple targets. Recombinant sequence‐defined secondary nanobodies with site‐specific fluorophore conjugation may also provide additional advantages in stoichiometric control and reagent reproducibility and represent an important direction for future refinement of this workflow.

A major advantage of umIF lies in its flexibility for large, high‐plex antibody panel design. Because the workflow improves the labeling efficiency across different IF methods and supports hybrid use of same‐species antibody‐nanobody complexes, direct dye‐conjugated primaries, and two‐step indirect IgG‐based IF, umIF enables more flexible use of high‐affinity reagents without host‐species constraints. This capability simplifies workflow, reduces experimental time, and broadens the practical options for panel design in multiplexed IF. These practical considerations make umIF well‐suited for high‐plex protein profiling in both discovery‐based research and standardized clinical workflows.

The flexibility of umIF is further supported by its compatibility with an established 4i‐based cyclic multiplexed imaging workflow [3]. Although 4i readily integrates with conventional IgG‐based secondary antibody staining, multiplexing within each cycle is generally constrained by host‐species compatibility, often limiting co‐incubation to two primary antibody species, and sometimes to only one when suitable antibodies from an additional host are unavailable. In this setting, umIF expands practical antibody‐panel design by enabling host‐independent one‐step complexes while remaining compatible with direct primary‐antibody staining and two‐step IF in the same multicycle imaging strategy. The improved labeling efficiency observed at short incubation times may also help shorten staining steps within iterative workflows, thereby improving the practical throughput of cyclic multiplexed imaging. In addition, the multicycle analysis showed preserved structural labeling patterns through Cycle 11 together with efficient elution across direct 1‐step, umIF‐complex, and umIF 2‐step staining formats. The lower residual post‐elution background observed for umIF complexes relative to conventional 2‐step IF further supports the practical utility of umIF in iterative high‐plex imaging.

Beyond spatial mapping of epithelial, stromal, and immune microenvironment in advanced adenomas, ulcerative colitis and the mouse model of *Kras* ^G12D^ *Lkb1* ^−/−^‐driven lung tumors, these application studies show that umIF can enable biologically meaningful discovery by resolving spatially organized differences in chromatin‐associated states across immune cell populations. In KL tumors, regulatory T cells exhibit elevated H3K4me3 expression, suggesting an activated Treg population with a comparatively active chromatin‐associated profile. Moreover, we identified two distinct morphologies in macrophages linked to differences in chromatin‐associated markers. Elongated macrophages, often intermixed within vimentin‐rich stromal regions surrounding the tumor, exhibit more active chromatin states whereas larger rounded macrophages show increased H3K27me3, reflecting a more repressive epigenetic state. These spatial patterns are consistent with prior literature linking macrophage morphology and state‐associated phenotypes [30], and they further illustrate the ability of umIF to associate cell identity, spatial context, morphology, and chromatin‐related markers within intact tissue architecture. More broadly, these application studies show that umIF can support spatially resolved profiling of epithelial, stromal, vascular, immune, and chromatin‐associated features in complex tissues. Orthogonal assays such as CUT&RUN [31] and scATAC‐seq [32] provide complementary measurements of chromatin state at population or single‐cell resolution, whereas umIF places chromatin‐associated markers in the spatial context of marker‐defined cell populations in situ.

## 4 Conclusion

In summary, umIF provides a practical and flexible framework for high‐plex spatial protein profiling on FFPE tissue. By leveraging macromolecular crowding to enhance antibody labeling efficiency, umIF achieves improved labeling performance across nuclear, cytoplasmic, and membrane targets while remaining compatible with standard immunofluorescence workflows. A key practical advantage of umIF is its ability to improve the detection of weak targets. At the same time, umIF supports host‐independent one‐step nanobody‐complex staining while also enhancing direct and two‐step immunofluorescence workflows, thereby expanding antibody‐panel design and facilitating scalable multiplexing across large panels.

Conceptually, umIF extends the principle of macromolecular crowding to tissue‐scale spatial proteomics. By improving labeling efficiency across multiple staining formats and remaining compatible with standard, off‐the‐shelf antibodies, umIF helps lower the technical barriers to high‐plex spatial biology and makes advanced tissue profiling more accessible for a broader range of laboratories. Its compatibility with direct, two‐step, host‐independent one‐step, and iterative multicycle workflows further enhances its practical utility for multiplexed tissue imaging. Applied to both clinical and preclinical tissues, umIF supports spatial profiling of epithelial, stromal, immune, and chromatin‐associated features within intact tissue architecture. By enabling integrated analysis of cell identity, morphology, spatial context, and chromatin‐associated states, umIF provides a practical platform for biologically meaningful discovery in studies of tissue organization, tumor‐immune interactions, therapeutic response and disease progression.

## 5 Methods

### 5.1 Human Tissue Sample Collection

Human samples were collected with approval from the Institutional Review Board (IRB) at the University of Pittsburgh (IRB #: STUDY23050085). Biopsies from patients with ulcerative colitis or advanced adenomas undergoing routine colonoscopy at the University of Pittsburgh Medical Center were collected under Institutional Review Board approval. Informed, written consent was obtained from all participants before sample collection. Samples were fixed in 10% neutral‐buffered formalin and processed by paraffin embedding using standard histology protocols. The FFPE tissue blocks were sectioned and processed for umIF staining and imaging using our custom‐built microscopy system [33].

### 5.2 Animal Tissue Sample Collection

Animal samples including mouse small intestine from wild‐type mice (C57BL/6J, The Jackson Laboratory, Stock No 000664) were collected as part of previously published projects [34, 35]. All animal procedures used to generate these tissues were reviewed and approved by the relevant Institutional Animal Care and Use Committee (IACUC) at the University of Pittsburgh under protocol number 26058353. The lung tumors harvested from four *Kras* ^G12D^ *Lkb1* ^−/−^ (KL) mice from one to three months. Their FFPE tissue blocks were a kind gift from Kwok‐Kin Wong. These FFPE tissue blocks were used for tissue sectioning and for umIF staining and imaging using our custom‐built microscopy system [33].

### 5.3 Tissue Sectioning and Processing

Formalin‐fixed paraffin‐embedded (FFPE) tissue blocks were sectioned at 3–5 µm thickness and mounted onto #1.5 gelatin‐coated coverslips. Sections were baked at 60°C for 1 h to ensure adhesion, followed by deparaffinization in xylene and rehydration through a graded ethanol series (100%, 95%, 70%, and 50%). Heat‐induced antigen retrieval was performed in citrate buffer (pH 6.0) to unmask cross‐linked epitopes (Figure S23A, steps (1)–(3)). To reduce tissue autofluorescence, sections were submerged in 1× PBS containing 4.5% H_2_O_2_ and 24 mm NaOH and exposed to LED light for 30 min. This process was repeated 2–3 times until background signals were minimized (Figure S23A, step (4)). Quenched samples were rinsed in 1× PBS and permeabilized with 0.2% Triton X‐100 for 10 min at room temperature (RT) (Figure S23A, step (5)). Sections were then blocked for 1 h at RT in blocking buffer containing 3% normal alpaca serum, 1% bovine serum albumin (BSA), 100 mm NH_4_Cl, and 150 mm maleimide in 1× PBS (Figure S23A, step (6)) to prevent permanent disulfide bonds forming between antibodies and the samples by reacting with free sulfhydryl groups, minimize nonspecific antibody binding, quench residual aldehydes, and prevent cross‐reactivity of Fc receptors. After these steps, sections were processed for umIF staining and imaging.

### 5.4 Preparation of Antibody‐Nanobody Complexes

A day prior to umIF staining, each unconjugated primary antibody (1°Ab) (Tables S14–S20) was pre‐incubated with the corresponding dye‐conjugated secondary nanobody (2°Nb) labeled with Alexa Fluor 555 (AF555), Alexa Fluor 568 (AF568), or Alexa Fluor 647 (AF647) at 4°C overnight in sterile tubes. The 1°Ab:2°Nb mixing ratio was optimized by comparing ratios from 1:1 to 1:10, and a volume ratio of 1:4 was selected because it provided the best overall staining performance under the tested conditions (Figure S24). This ratio corresponds to an ∼40‐fold molar excess of nanobody over primary antibody (Figure S23B, steps (7)–(8)). In practical applications, this ratio may require further optimization depending on factors such as antibody affinity, reagent characteristics, fluorophore conjugation density, and tissue context.

Additionally, Alexa Fluor 405 (AF405) and Alexa Fluor 488 (AF488)–conjugated nanobodies can be incorporated to further expand multiplexing capacity. On the following day, excess unlabeled secondary nanobodies were added to saturate residual Fc sites on the primary antibodies, thereby minimizing cross‐binding during umIF staining (Figure S23B, step (9)). In parallel, dye‐conjugated primary antibodies underwent the same Fc‐blocking procedure (Figure S23B, steps (10)–(11); Figure S25). Finally, Fc‐blocked nanobody complexes and dye‐conjugated primaries were pooled together in umIF buffer for multiplexed immunolabeling.

### 5.5 Optimization of umIF Buffer

The umIF buffer was prepared by supplementing 1x PBS containing 3% normal alpaca serum, 1% bovine serum albumin (BSA), 100 mm NH_4_Cl, and 0.1% Tween‐20 with dextran sulfate (DS; 0%, 0.1%, 1.0%, 2.5%, 5.0%, or 10% w/v), either with or without Denhardt's solution (DH; 1 ×; 1% Ficoll type 400, 1% polyvinylpyrrolidone, and 1% bovine serum albumin). DS stock (20% w/v) was prepared fresh and diluted immediately before use. Prepared buffers were stored at 4°C and used within one week or at −20°C for long‐term storage.

Antibody‐nanobody complexes were prepared by pre‐incubating unconjugated primary antibodies (1°Ab) with dye‐conjugated secondary nanobodies (2°Nb) at a volume ratio of 1:4 overnight at 4°C, followed by Fc blocking with excess unlabeled nanobodies. For optimization, tissue section was blocked (3% normal alpaca serum, 1% bovine serum albumin, 100 mm NH_4_Cl, 150 mm maleimide in PBS) and co‐stained for 2 h at room temperature with same‐host antibody‐nanobody complexes targeting E‐cadherin and PCNA. *Complexes* were assembled with spectrally distinct fluorescent nanobodies (AF594 and AF647, respectively). Parallel sections were processed under identical conditions with or without Denhardt's solution (DH) to assess the effect of macromolecular crowding.

### 5.6 One‐Step umIF Staining

For umIF labeling, Fc‐blocked *complexes* (1°Ab + 2°Nb) and dye‐conjugated primary antibodies (1°Ab) were pooled in umIF buffer and applied directly to permeabilized and serum‐blocked FFPE tissue sections (Figure S23C, step 12–13). Incubations were performed as short as 30 min or extended period at RT, depending on the marker abundance and desired signal strength. Following incubation, samples were washed three times with 1× PBS to remove unbound complexes. After each staining cycle, samples were imaged immediately (Figure S23C, step 14). To enable iterative multiplexing, fluorophores and bound complexes were eluted using freshly prepared elution buffer (0.5 m glycine, 3 m urea, 3 m guanidinium chloride, and 70mm TCEP, pH 2.5) for 5 min, repeated twice (Figure S23C, step 15). Samples were subsequently rinsed in 1× PBS, post‐blocked for 15 min in the same blocking buffer used during initial preparation (Figure S23C, step 16) and subjected to additional staining cycles as needed. This iterative workflow (staining → imaging → elution → post‐blocking) was repeated up to 10–15 cycles without detectable loss of tissue integrity or antigenicity, allowing high‐dimensional profiling of epithelial, stromal, and immune markers in the same tissue section.

### 5.7 Comparison of umIF and Conventional Immunofluorescence (conv) for Co‐Staining of *complexes* and 1°Abs

FFPE sections were processed as described above. For umIF, Fc‐blocked *complexes* (1°Ab + 2°Nb) and Fc‐blocked dye‐conjugated primary antibodies (1°Ab) were pooled in the optimized umIF buffer containing macromolecular crowding agents and incubated for 2 h at room temperature. For conventional staining (conv), the same *complexes* and 1°Abs were prepared in conventional blocking buffer (1× PBS supplemented with 3% normal alpaca serum, 1% bovine serum albumin (BSA), 100 mm NH_4_Cl, and 0.1% Tween‐20) and incubated under identical conditions. After staining, all sections were washed in 1× PBS and mounted in imaging buffer. Images were acquired using identical microscope settings to allow direct comparison of labeling efficiency and signal uniformity.

### 5.8 Comparison of *umIF 2‐Step* and Conventional IF Staining

FFPE tissues were used for the evaluation of two‐step immunofluorescence staining. For *umIF 2‐step*, sections were incubated with unconjugated primary antibodies followed by dye‐conjugated secondary IgG (H+L) antibodies or secondary nanobodies (VHH Fragment Alpaca IgG (H+L) (2 h each at room temperature) in the optimized umIF buffer, with PBS washes between steps. For conventional two‐step immunofluorescence staining, the same antibody combinations were applied in conventional blocking buffer under identical incubation conditions. Parallel sections were processed in the same manner, and images were acquired using identical microscope settings to directly compare labeling specificity, intensity, and subcellular localization.

### 5.9 Conjugation of Secondary IgG (H+L) Antibodies

Secondary IgG (H+L) antibodies (Table S14) were conjugated to fluorophores using a standard NHS‐ester labeling protocol (Thermo Fisher Scientific). Briefly, antibodies in 1× PBS were adjusted to 0.1 m sodium bicarbonate (NaHCO_3_, pH 8.3) by dilution from a 1 m stock and mixed with fluorophore NHS‐esters dissolved in DMSO at a dye‐to‐protein molar ratio of ∼5:1. The reaction was incubated for 30 min at room temperature in the dark. Excess unreacted dye was removed by size‐exclusion chromatography, and fluorophore‐conjugated antibodies were eluted into PBS containing 0.05% sodium azide and 1 mg/ml BSA for short‐term storage at 4°C. For long‐term storage, conjugated antibodies were supplemented with 50% glycerol, aliquoted, and stored at −20°C. The degree of labeling (DOL) was determined spectrophotometrically using NanoDrop 2000 microspectrophotometer (Thermo Fisher Scientific, NanoDrop 2000) according to the manufacturer's instructions.

### 5.10 Data Acquisition and Image Processing

Multiplexed fluorescence and bright‐field images were acquired using a custom‐built wide‐field multimodal microscope (Figure S23D, step 17–18), and data were processed following the pipeline as previously described [33, 36]. Briefly, optimal focal planes were identified using normalized variance analysis of bright‐field images, and z‐stack acquisition was used to correct field curvature across the extended field of view. Flat‐field correction, stitching, registration, and segmentation were performed as previously reported [33, 36]. For each given target, all comparative images within the same experiment including comparisons across time points, staining conditions, and antibody‐labeling formats were acquired using identical imaging parameters, including exposure time, gain, laser power, and related acquisition settings, to enable direct comparison of signal intensity. Because different targets and different fluorophores varied in signal level and optimal imaging range, acquisition settings were adjusted between different targets as needed.

### 5.11 Quantitative Image Analysis

For quantitative analysis, fluorescence images from each comparison category were spatially registered using the corresponding phase images as described previously [36]. For image sets that could be aligned with pixel‐level accuracy, a reference fluorescence image with the strongest signal was used to define the signal region by Otsu thresholding. This binary mask served as the master mask and was applied to all registered images in the corresponding series. The mean signal was calculated as the average fluorescence intensity of pixels within the mask, whereas the mean background was calculated from pixels outside the mask in the same image. The contrast‐to‐noise ratio (CNR) was computed as (µ_*s*_ − µ_*b*_)/σ_*b*_, where µ_*s*_ and µ_*b*_ represent the mean signal and background intensities, respectively, and σ_*b*_ represents the standard deviation of the background. For image sets acquired from serial tissue sections, where precise pixel‐level registration was not feasible, Otsu thresholding was performed independently on each image to generate an individual signal mask, and the same signal, background and CNR were then calculated for each image using its corresponding mask.

### 5.12 Statistical Analysis

All statistical analyses were performed using GraphPad Prism (version 9.0). Comparisons between two groups were assessed using unpaired two‐tailed Student's *t*‐tests. Comparisons among multiple groups were performed using one‐way ANOVA followed by Tukey's multiple‐comparisons test. A *p*‐value < 0.05 was considered statistically significant.

### 5.13 Cell Segmentation and Classification in the Murine Model of Lung Carcinogenesis

For each specimen, single‐cell segmentation was performed using Cellpose v3 [37] with additional manual corrections to ensure accurate boundary delineation. Binary marker masks were generated for each marker of interest, including Cytokeratin, CD31, CD4, CD8, CD68, and CD45, using Otsu or manual thresholding. For the functional markers H3K27me3 and RNAPII‐S2P, intensity values within segmented cells were normalized to the [0,1] range per specimen. The lower bound was defined at the 1st percentile of pixel intensities, and the upper bound was defined at the 99th percentile. Each cell was classified according to the dominant marker signal within its segmented area. Cells positive for CD4, CD8, or CD68 were additionally annotated as CD45^+^. Cells that only expressed CD45 were exclusively classified as “Other CD45^+^”. Cell classification and spatial coordinates were retained to allow direct correspondence with segmentation masks. Average normalized values for H3K27me3 and RNAPII‐S2P were computed for each segmented cell. Classified segmentations were visualized and color‐coded by cell type (Figure 8A, B).

### 5.14 Quantitative Analysis of Epigenetic and Transcriptional Markers

For epigenetic analysis (Figure 8C), normalized intensity distributions for H3K4me3, MCM7 and RNAPII‐S2P were computed for cytotoxic T cells (CD8^+^), regulatory T cells (Tregs; CD4^+^FoxP3^+^), macrophages (CD68^+^). Violin plots were constructed using GraphPad Prism (version 9.0) for visualization.

### 5.15 Macrophage Clustering and Quantitative Analysis

Cells annotated as macrophages were extracted from segmented single‐cell datasets. Two quantitative parameters including cell area (µm^2^) and H3K27me3 fluorescence intensity were used for unsupervised clustering with the K‐means algorithm (K = 2) implemented in scikit‐learn (v1.5.0) using Python (v3.11). The two resulting clusters corresponded to distinct macrophage morphologies and were designated as elongated (Cluster 0) and rounded (Cluster 1). Cluster assignments were appended to each macrophage record and exported for downstream analysis.

For visualization, violin plots were generated in GraphPad Prism (version 9.0) from the clustered dataset to illustrate the distribution of area and H3K27me3 intensity across the two macrophage morphotypes. Each violin plot depicts the full data distribution with the median and interquartile range indicated.

## Conflicts of Interest

The authors declare no competing interests.

## Supporting information




**Supporting File 1**: smtd70783‐sup‐0001‐SuppMat.pdf.

**Supporting File 2**: smtd70783‐sup‐0002‐TableS1‐S21.pdf.

## Data Availability

All data supporting the findings of this study are available within the article and its supplementary information files. Due to the large size of the imaging datasets, additional data are available from the corresponding author upon reasonable request.
