# Supplementary material for: A Versatile Multiplexed Immunofluorescence Strategy for Efficient, Host‐Independent, and Scalable Spatial Protein Profiling
Source: Small Methods. 2026 Jun 17;10(14):e00009. doi: 10.1002/smtd.202600009 (PMC13375603; doi:10.1002/smtd.202600009)
Supplement: Supplementary file 1 — Supporting File 1: smtd70783‐sup‐0001‐SuppMat.pdf. [file SMTD-10-e00009-s001.pdf]

A versatile multiplexed immunofluorescence strategy for efficient, host-independent, and scalable spatial protein profiling

Phuong Nguyen[1], Hongqiang Ma[1], Dimitrios Gotsis[2], Maomao Chen[3], Chaojie Zhang[1], Tushar Talukder Showrav[1], Marc Schwartz[3], Brenda Diergaarde[4], Robert E. Schoen[3], Rhonda M. Brand[3], Hua Zhang[5], Yang Liu[*1, 2, 6]

1. Grainger College of Engineering, Department of Bioengineering, University of Illinois Urbana-Champaign, Urbana, IL, USA
2. Grainger College of Engineering, Department of Electrical and Computer Engineering, University of Illinois Urbana-Champaign, Urbana, IL, USA
3. Department of Medicine, Division of Gastroenterology, Hepatology and Nutrition, University of Pittsburgh, University of Pittsburgh Medical Center, Pittsburgh, PA, USA
4. Department of Human Genetics, University of Pittsburgh School of Public Health, Pittsburgh, PA, USA.
5. Department of Medicine, Division of Hematology/Oncology, University of Pittsburgh School of Medicine, Pittsburgh, Pennsylvania, USA.
6. Cancer Center at Illinois, Beckman Institute for Advanced Science and Technology, University of Illinois Urbana-Champaign, Urbana, IL, USA

*Correspondence: Yang Liu (liuy46@illinois.edu)

Supplementary Figures

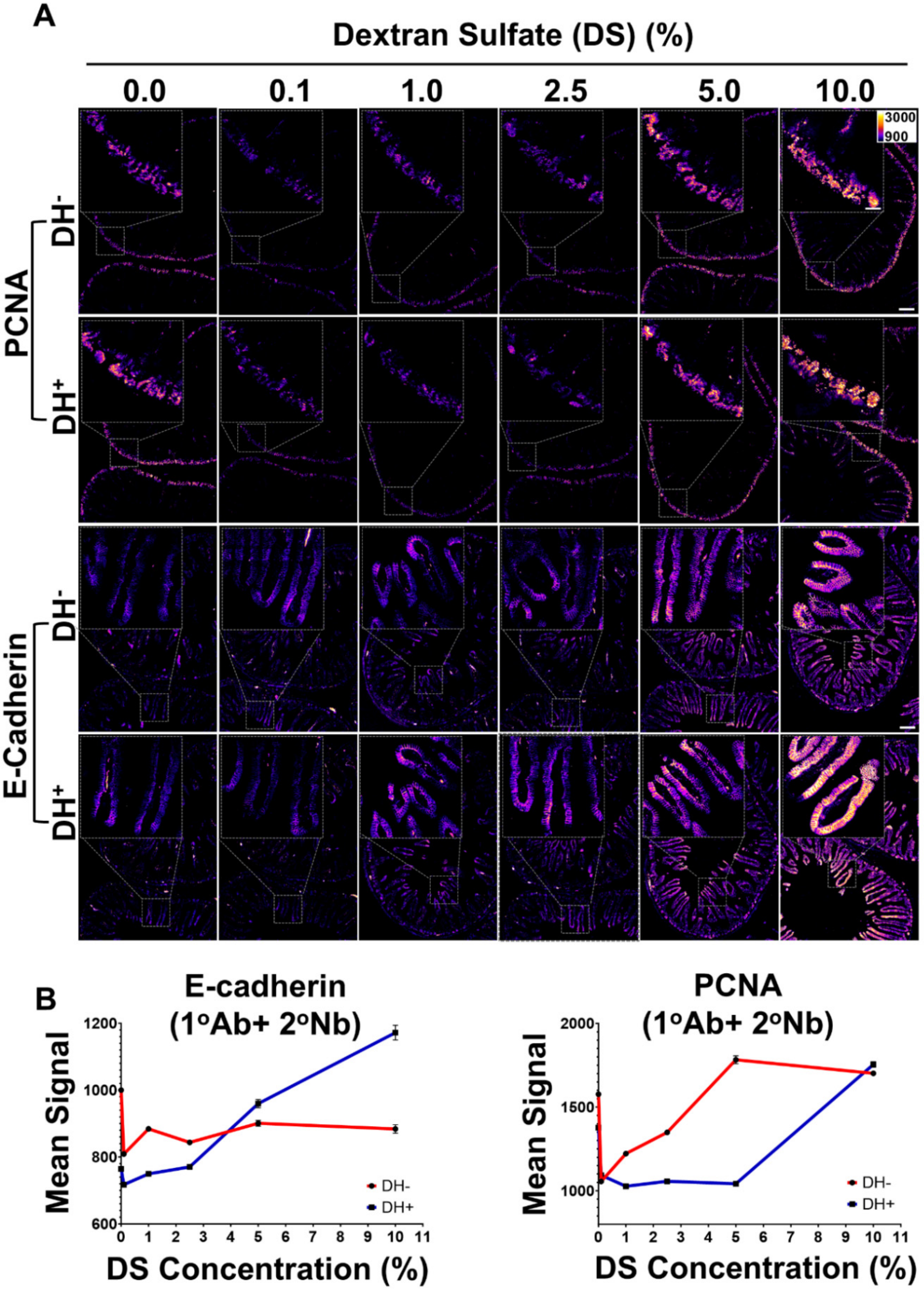

Supplementary Figure S1: Buffer optimization for *umIF complexes* using one-step incubation of same host antibody-nanobody complexes (1°Ab + 2°Nb).

(**A**) Representative umIF images of mouse small intestine co-stained with same-host antibody-nanobody complexes targeting the nuclear proliferation marker PCNA (rabbit anti-PCNA, top) and the epithelial

membrane adhesion marker E-cadherin (rabbit anti–E-cadherin, bottom) across increasing concentrations of the macromolecular crowding agent dextran sulfate (DS; 0–10%), with (DH^+) or without (DH^-) Denhardt's solution containing 1% Ficoll (type 400), 1% polyvinylpyrrolidone, and 1% bovine serum albumin (BSA). Insets show enlarged views of the boxed regions. (**B**) Quantification of average fluorescence intensity for E-cadherin (left) and PCNA (right) across different concentrations of dextran sulfate (DS) under DH^+ and $DH^−$ conditions as shown in (A). Data are presented as mean ± standard error of the mean (SEM) using XY line plots with connecting colored lines (red, $DH^−$; blue, DH^+). Individual values are shown as black circles ($DH^−$) or black squares (DH^+). For E-cadherin under $DH^−$ conditions, fluorescence intensities (mean ± SD, a.u.) were 1000 ± 3.738 (n = 1034 ROIs), 809.1 ± 3.150 (n = 933 ROIs), 884.2 ± 4.123 (n = 424 ROIs), 843.8 ± 6.159 (n = 309 ROIs), 901.1 ± 8.741 (n = 194 ROIs), and 884.0 ± 12.56 (n = 140 ROIs) at 0, 0.1, 1.0, 2.5, 5.0, and 10.0% DS, respectively. Under DH^+ conditions, fluorescence intensities were 764.6 ± 4.360 (n = 238 ROIs), 717.5 ± 6.188 (n = 196 ROIs), 749.8 ± 5.707 (n = 173 ROIs), 770.5 ± 6.587 (n = 176 ROIs), 959.8 ± 12.28 (n = 166 ROIs), and 1172 ± 21.79 (n = 158 ROIs) at 0, 0.1, 1.0, 2.5, 5.0, and 10.0% DS, respectively. For PCNA under $DH^−$ conditions, fluorescence intensities (mean ± SEM, a.u.) were 1577 ± 13.37 (n = 194 ROIs), 1056 ± 5.139 (n = 151 ROIs), 1222 ± 8.806 (n = 112 ROIs), 1349 ± 9.543 (n = 98 ROIs), 1783 ± 23.81 (n = 147 ROIs), and 1702 ± 13.61 (n = 165 ROIs) at 0, 0.1, 1.0, 2.5, 5.0, and 10.0% DS, respectively. Under DH^+ conditions, fluorescence intensities were 1377 ± 15.04 (n = 123 ROIs), 1093 ± 7.730 (n = 145 ROIs), 1027 ± 7.706 (n = 126 ROIs), 1057 ± 7.490 (n = 180 ROIs), 1042 ± 11.58 (n = 157 ROIs), and 1755 ± 16.92 (n = 176 ROIs) at 0, 0.1, 1.0, 2.5, 5.0, and 10.0% DS, respectively. Individual values are shown as black circles ($DH^−$) or black squares (DH^+). Both markers exhibited dose-dependent modulation in the presence of Denhardt (DH^+) at 10% dextran sulfate (DS), yielding consistently higher labeling efficiency compared with the controls with DH. Scale bars, 200 µm (overview) and 50 µm (insets).

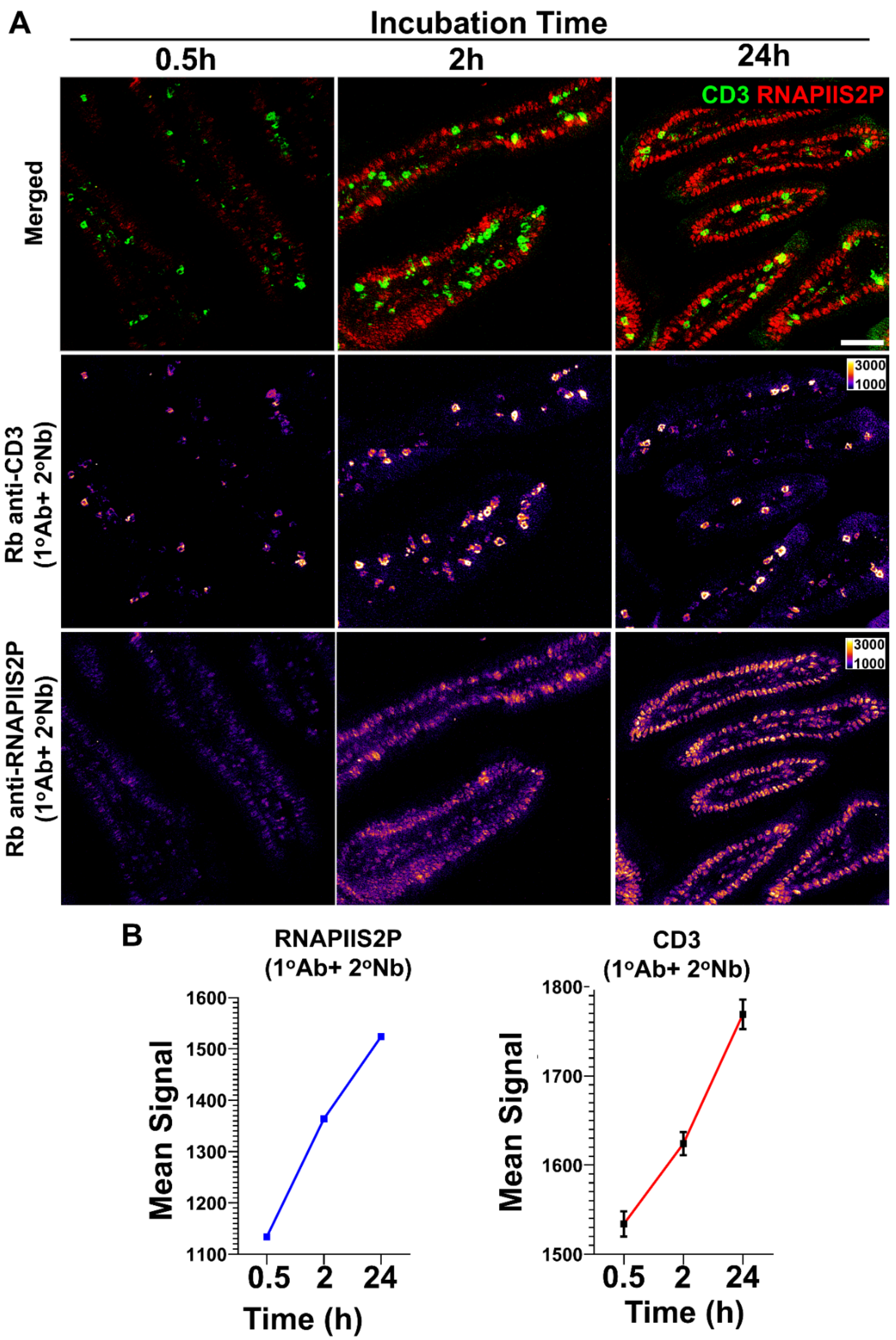

Supplementary Figure S2. Effect of incubation time on *umIF complexes* for one-step labeling efficiency using same-host antibody-nanobody complexes (1°Ab + 2°Nb).

(**A**) Representative fluorescence images of mouse small intestine co-stained with same-host antibody-nanobody complexes targeting CD3 (rabbit anti-CD3, green) and RNAPII S2P (rabbit anti-RNAPII S2P, red) after incubation for 30 minutes (30m), 2 hours (2h), or 24 hours. Merged panels show a progressive increase in labeling signals with longer incubation times. (**B**) Quantification of mean fluorescence

intensity for RNAPII-S2P (left) and CD3 (right) across incubation times as shown in (A). Data are presented as mean ± SEM using XY line plots with connecting colored lines (blue, RNAPII S2P; red, CD3) and individual values shown as black squares (RNAPII S2P) or black circles (CD3). For RNAPII-S2P, fluorescence intensities, reported as mean ± SD in arbitrary units (a.u.), were 1134.0 ± 1.3, 1364.0 ± 2.1, and 1524.0 ± 2.0 after 30 min, 2 h, and 24 h incubation, respectively. For CD3, fluorescence intensities were 1534.0 ± 14.1, 1624.0 ± 13.0, and 1769.0 ± 16.6 after 30 min, 2 h, and 24 h incubation, respectively. The number of regions with positive signals is ~400-9000. Data are presented as XY line plots with connecting colored lines (blue, RNAPII-S2P; red, CD3), with individual values shown as black squares (RNAPII-S2P) or black circles (CD3). Both markers exhibited progressively increased labeling intensity with longer incubation times, with 24 h incubation showing the strongest signals without evidence of antibody aggregation. Scale bars: 200 µm (overview) and 50 µm (insets).

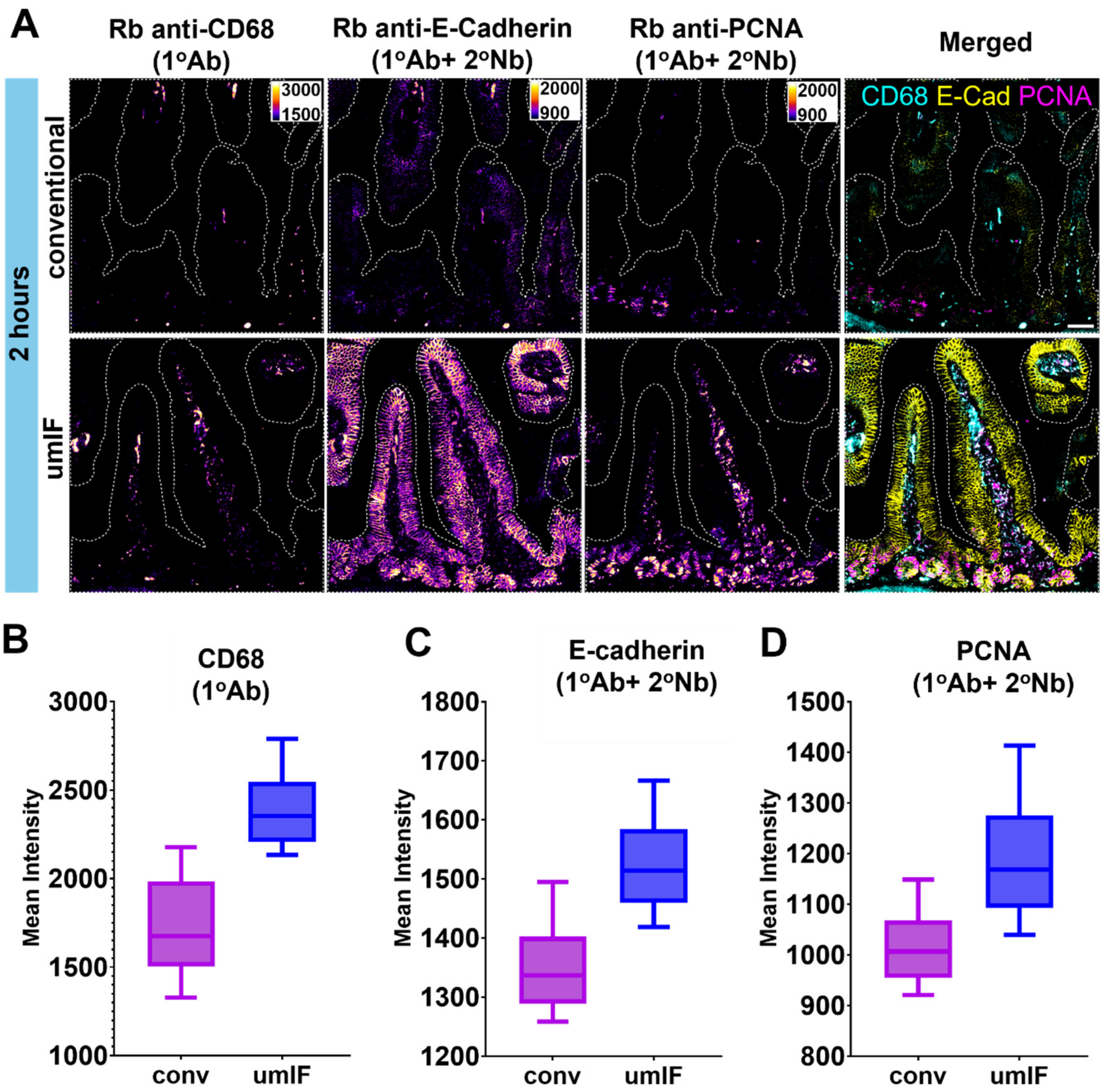

Supplementary Figure S3: umIF enhances one-step labeling efficiency of *complexes* and 1°Abs compared with conventional immunofluorescence (conv).

(**A**) Representative fluorescence images of mouse small intestine co-stained with rabbit-derived dye-conjugated primary antibodies (1°Ab) against CD68 (cyan) and same-host antibody-nanobody complexes (1°Ab + 2°Nb) targeting E-cadherin (yellow) and PCNA (magenta) using conventional immunofluorescence (conv, top) or umIF (bottom) with a 2-hour (2 hr) incubation. Conventional co-incubation of dye-conjugated primaries and antibody-nanobody complexes for the three targets produced weaker and less uniform labeling, whereas umIF generated stronger and more uniform staining. (**B–D**) Quantification of mean fluorescence intensity for CD68 (B), E-cadherin (C), and PCNA (D) as shown in (A). Data are presented as box-and-whisker plots (5th–95th percentile). For CD68, fluorescence intensities, reported as mean ± SD in arbitrary units (a.u.), were 1744.0 ± 309.7 for conventional immunofluorescence and 2429.0 ± 405.6 for umIF. For E-cadherin, fluorescence intensities were 1371.0 ± 171.3 for conventional immunofluorescence and 1537.0 ± 141.4 for umIF. For PCNA, fluorescence intensities were 1026.0 ± 141.3 for conventional immunofluorescence and 1203.0 ± 169.4 for umIF. The number of regions with positive signals is ~50-8000. umIF consistently produced stronger labeling signals compared with conventional immunofluorescence. Scale bars, 50 μm.

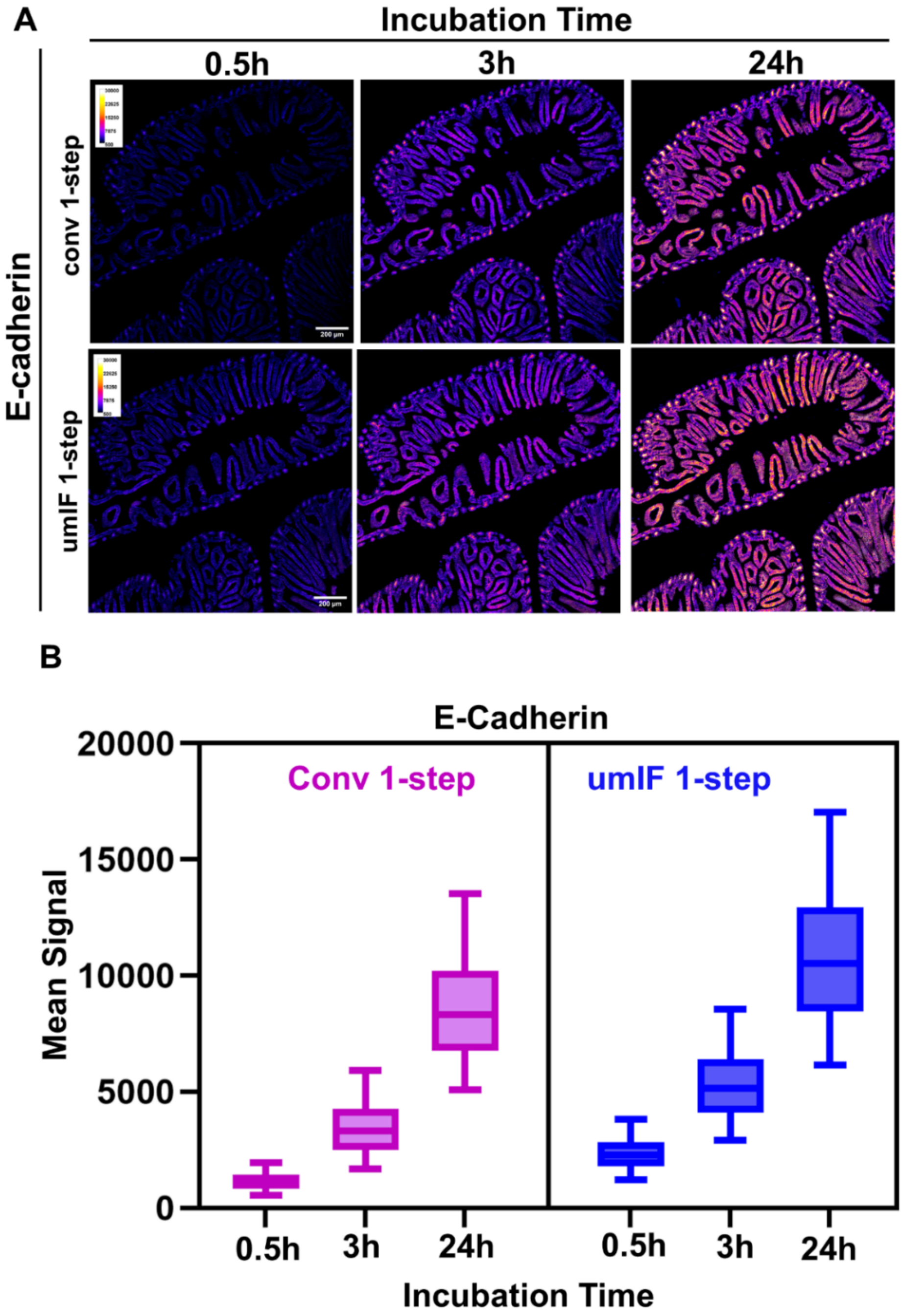

Supplementary Figure S4: The crowding-enhanced umIF buffer increases signal accumulation of dye-conjugated anti-E-cadherin primary antibody during staining.

(**A**) Representative immunofluorescence images of mouse intestine tissue stained with dye-conjugated anti-E-cadherin primary antibody under conventional and umIF buffer conditions at increasing incubation times (30 min, 3 hours, and 24 hours). The crowding-enhanced umIF buffer increased signal intensity across the entire incubation time course compared with conventional conditions. (**B**) Quantification of mean fluorescence intensity is presented as box-and-whisker plots (5th–95th percentile). For conventional staining, fluorescence intensities (mean ± SD, a.u.) were 1155.0 ± 443.3, 3501.0 ± 1330.0, and 8689.0 ± 2641.0 after 0.5 h, 3 h, and 24 h incubation, respectively. For umIF staining, fluorescence intensities were 2362.0 ± 803.5, 5372.0 ± 1774.0, and 10940.0 ± 3418.0 after 0.5 h, 3 h, and 24 h incubation, respectively. The number of regions with positive signals is ~5000-6000. umIF consistently enhanced signal accumulation compared with conventional staining across all incubation times. Scale bars, 200 µm. The corresponding fluorescence intensity from the labeled target, together with the CNR, are provided in Supplementary Table S3.

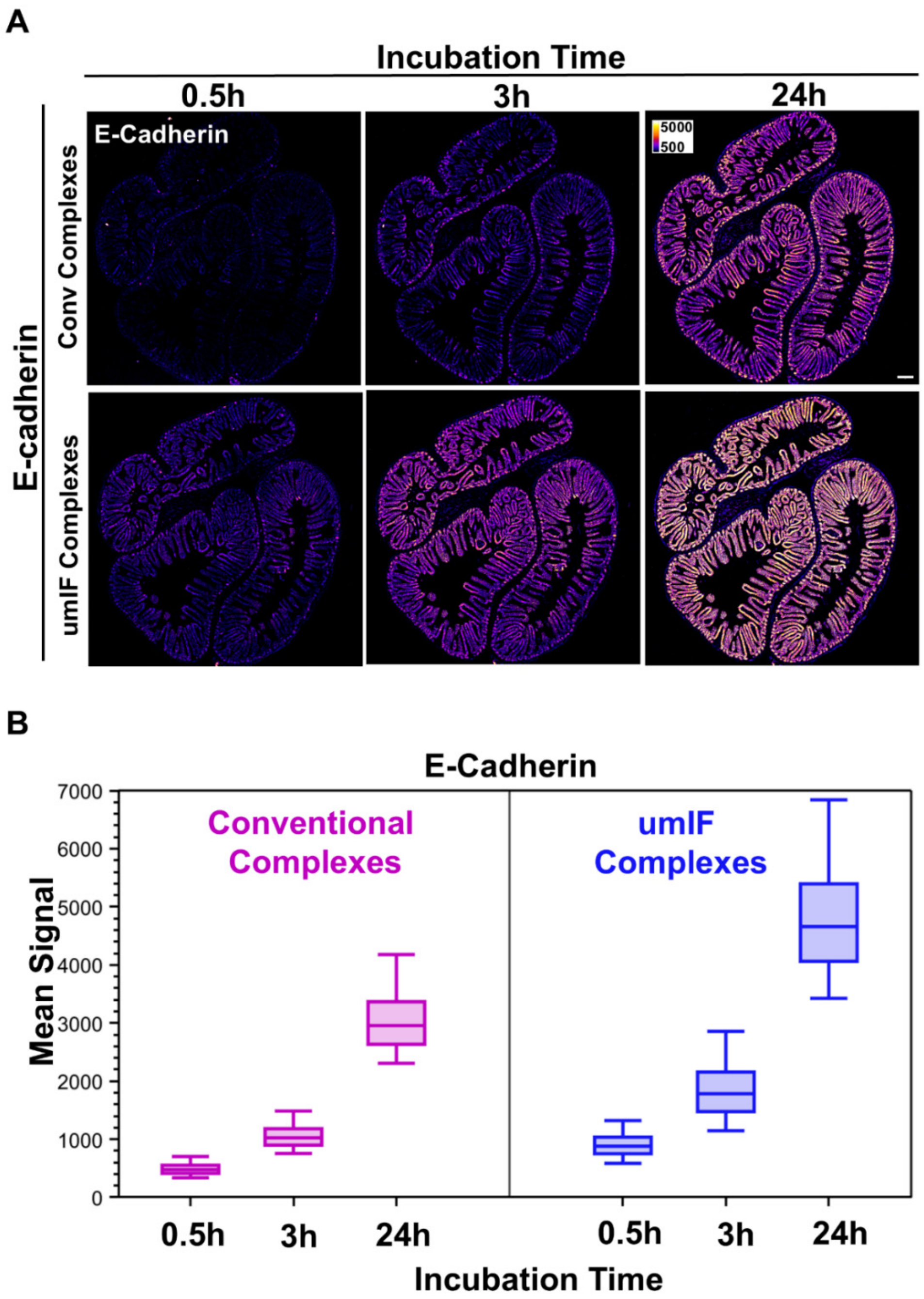

Supplementary Figure S5: The crowding-enhanced umIF buffer promotes signal accumulation of anti-E-cadherin antibody–nanobody complexes during staining.

(**A**) Representative immunofluorescence images of mouse intestine tissue stained with anti-E-cadherin antibody-nanobody complexes (1°Ab + 2°Nb) under conventional and umIF buffer conditions at increasing incubation times (0.5 h, 3 hours, and 24 hours). The crowding-enhanced umIF buffer increased signal intensity across the incubation time course. (**B**) Quantification of mean fluorescence intensity for E-cadherin staining with antibody-nanobody complexes, presented as box-and-whisker plots (5th–95th percentile), showing greater signal accumulation over time under umIF conditions than under conventional conditions. Scale bars, 200 µm. The corresponding mean ± SD values of fluorescence intensity from the labeled target, together with the CNR, are provided in Supplementary Table S4.

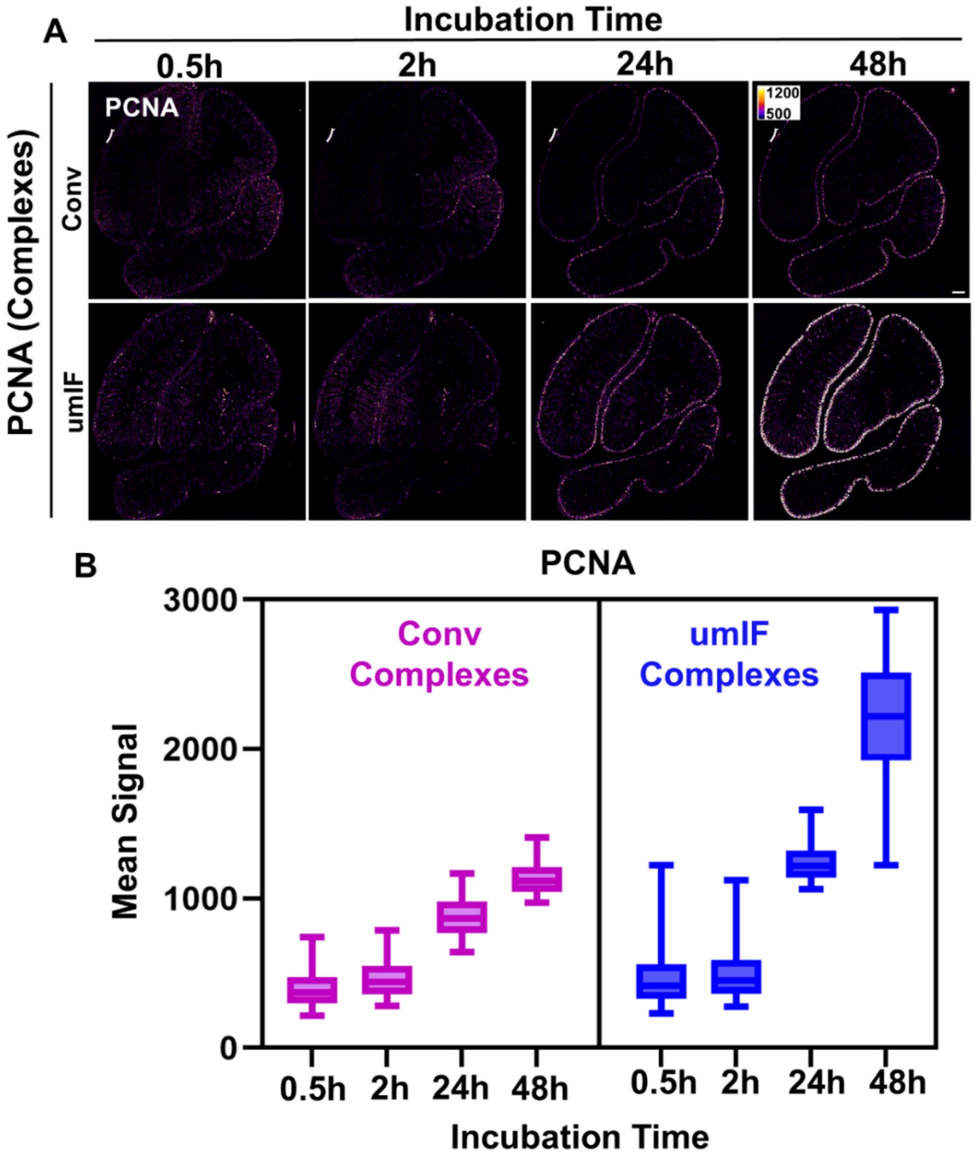

Supplementary Figure S6: The crowding-enhanced umIF buffer promotes signal accumulation of anti-PCNA antibody–nanobody complexes during staining.

(**A**) Representative immunofluorescence images of mouse intestine tissue stained with anti-PCNA antibody-nanobody complexes (1°Ab + 2°Nb) under conventional and umIF buffer conditions at increasing incubation times (0.5 h, 3 hours, and 24 hours, 48 hours). The crowding-enhanced umIF buffer increased signal intensity across the incubation time course. (**B**) Quantification of mean fluorescence intensity for PCNA staining with antibody-nanobody complexes, presented as box-and-whisker plots (5th–95th percentile). For conventional complexes, fluorescence intensities were quantified from registered image sets using the same segmented regions identified in the 48 h incubation images. The same segmented regions were then applied to the corresponding registered images at earlier time points. Fluorescence intensities, reported as mean ± SD in arbitrary units (a.u.), were 410.7 ± 196.6, 479.1 ± 212.9, 883.0 ± 191.5, and 1152.0 ± 201.8 after 0.5 h, 2 h, 24 h, and 48 h incubation, respectively, based on the same set of 1281 segmented regions. For umIF complexes, the same registration and ROI-transfer strategy was used, with fluorescence intensities of 508.9 ± 304.9, 542.0 ± 351.4, 1288.0 ± 412.3, and 2190.0 ± 555.8 after 0.5 h, 2 h, 24 h, and 48 h incubation, respectively, based on the same set of 1192 segmented regions. umIF consistently enhanced signal accumulation compared with conventional staining across all incubation times. Scale bars, 200 µm. The corresponding contrast-to-noise ratio (CNR) values are provided in Supplementary Table S5.

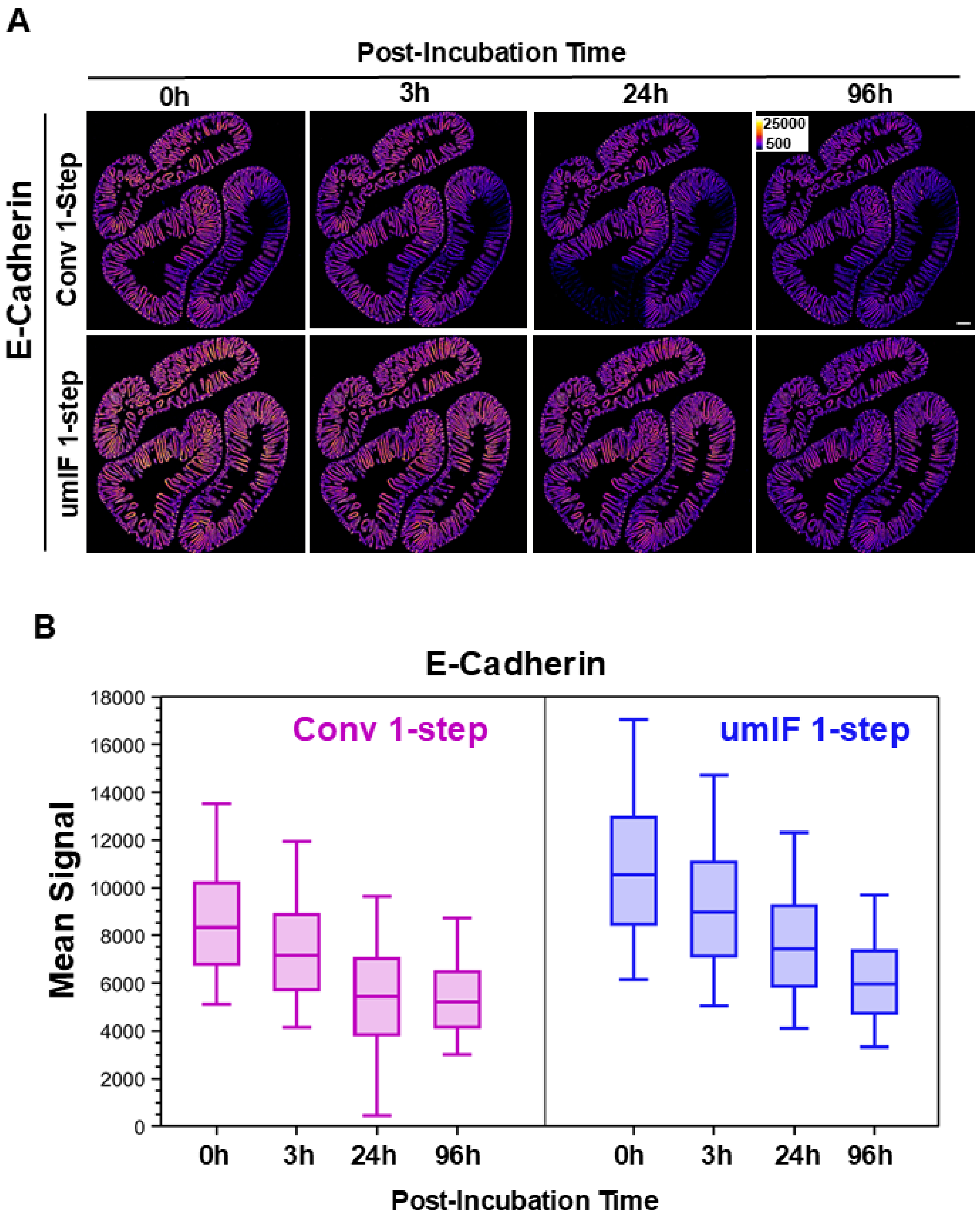

Supplementary Figure S7: Signal retention of dye-conjugated anti-E-cadherin primary antibody after incubation under conventional and umIF buffer conditions.

(**A**) Representative immunofluorescence images of mouse intestine tissue stained with dye-conjugated anti-E-cadherin primary antibody for 24 hours under conventional and umIF buffer conditions, followed by an immediate measurement (0 h), and post-incubation holding for 3 hours, 24 hours, and 96 hours. Both conditions exhibited gradual signal decrease over time, while the overall decay profiles were similar. (**B**) Quantification of mean fluorescence intensity for E-cadherin, presented as box-and-whisker plots (5th-95th percentile), showing comparable signal retention trends under conventional and umIF conditions across the post-incubation time course. Scale bars, 200 µm. The corresponding mean ± SD values of fluorescence intensity from the labeled target, together with the CNR, are provided in Supplementary Table S6.

A

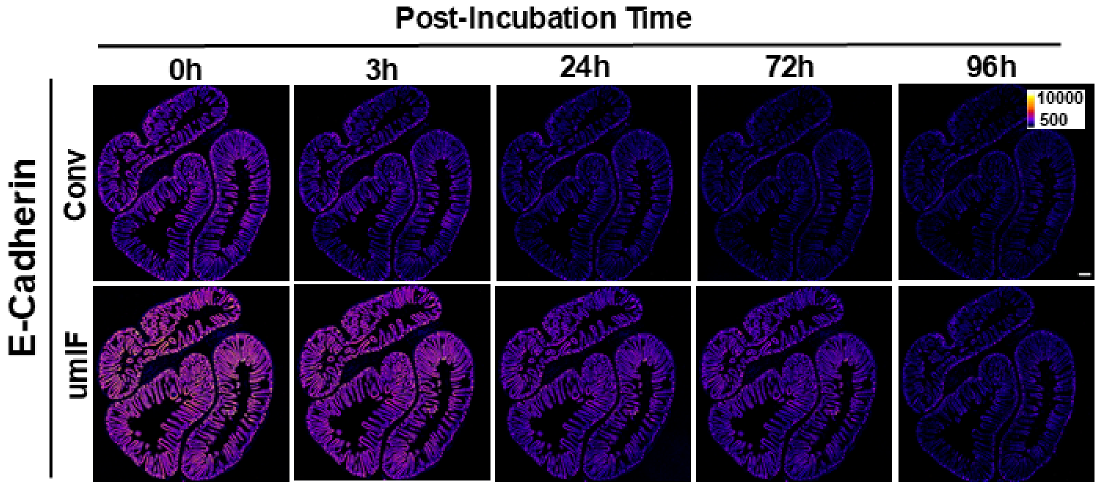

B

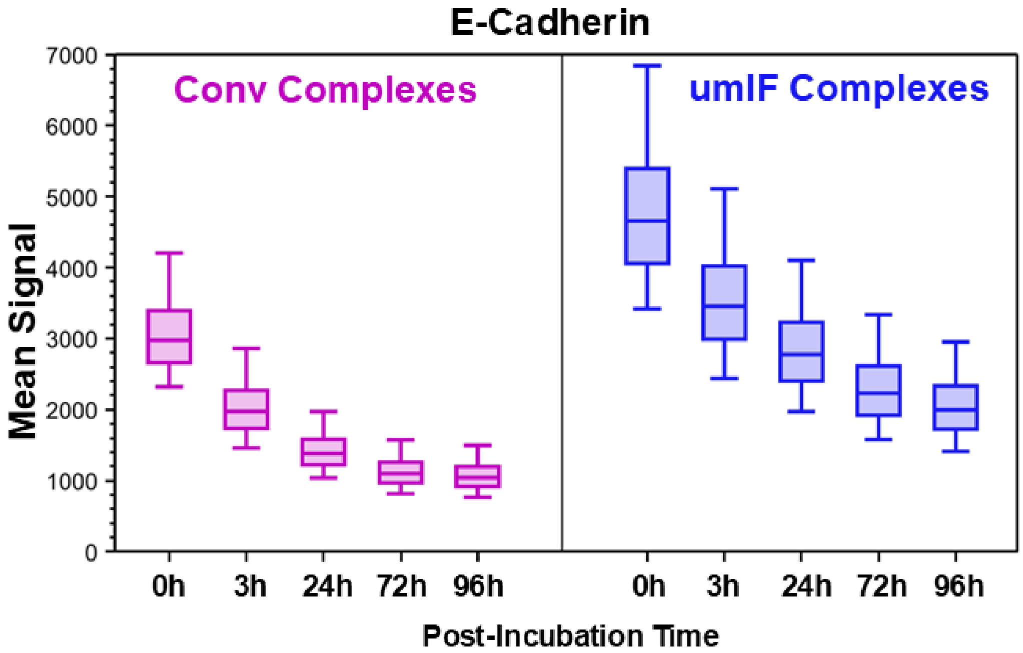

Supplementary Figure S8: Signal retention of anti-E-cadherin antibody-nanobody complexes after incubation under conventional and umIF buffer conditions.

(**A**) Representative immunofluorescence images of mouse intestine tissue stained with anti-E-cadherin antibody-nanobody complexes for 24 hours under conventional and umIF buffer conditions, followed by immediate imaging (0 h) or post-incubation holding for 3 hours, 24 hours, and 96 hours. Both conditions exhibited gradual signal decrease over time, while the overall decay profiles were similar. (**B**) Quantification of mean fluorescence intensity for E-cadherin, presented as box-and-whisker plots (5th–95th percentile), showing comparable signal retention trends under conventional and umIF conditions across the post-incubation time course. Scale bars, 200 µm. The corresponding mean ± SD values of fluorescence intensity from the labeled target, together with the CNR, are provided in Supplementary Table S7.

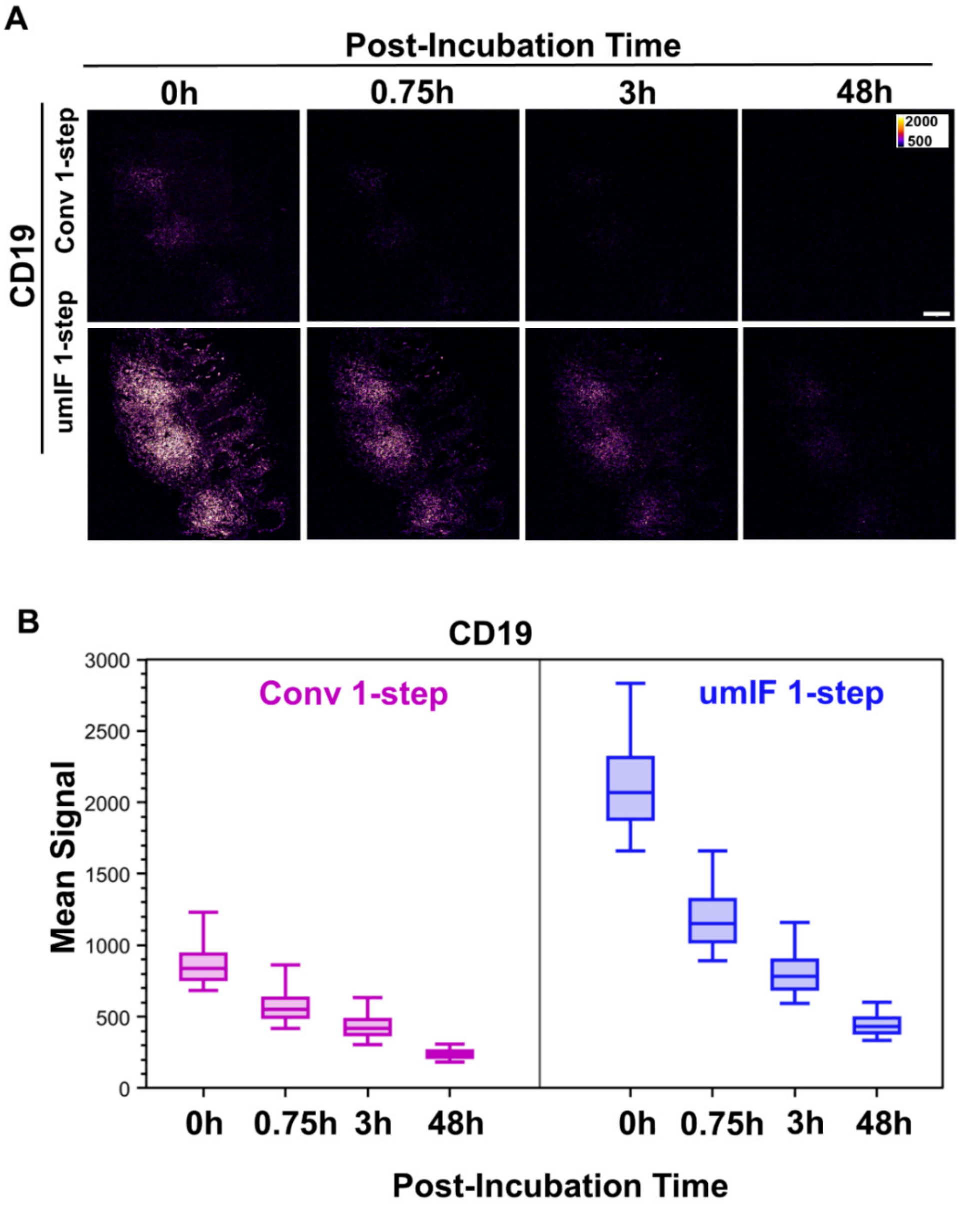

Supplementary Figure S9: Signal retention of dye-conjugated anti-CD19 primary antibody after incubation under conventional and umIF buffer conditions.

(**A**) Representative immunofluorescence images of human colon tissue with active ulcerative colitis stained with dye-conjugated anti-CD19 primary antibody for 24 hours under conventional and umIF buffer conditions, followed by immediate imaging (0 h) or post-incubation holding for 0.75 h, 3 h, and 48 h. In both conditions, signal decreased over time, with broadly similar overall decay profiles.
(**B**) Quantification of mean fluorescence intensity for CD19, presented as box-and-whisker plots (5th-95th percentile), showing progressive signal decrease across the post-incubation time course under both conventional and umIF conditions. For conventional staining, fluorescence intensities were quantified from registered image sets using the same segmented regions identified in the 48 h post-incubation images. The same segmented regions with positive signals were then applied to the corresponding registered images at earlier time points. Fluorescence intensities, reported as mean ± SD in arbitrary units (a.u.), were 879.2 ± 176.6, 582.2 ± 148.3, 444.0 ± 141.1, and 253.4 ± 134.9 at 0 h, 0.75 h, 3 h, and 48 h post-incubation, respectively, based on the same set of 313 regions. For umIF staining, the same registration and region-transfer strategy was used, with fluorescence intensities of 2138.0 ± 425.6, 1201.0 ± 274.0, 816.8 ± 201.1, and 447.7 ± 102.9 at 0 h, 0.75 h, 3 h, and 48 h post-incubation, respectively, based on the same set of 1813 regions. Scale bars, 200 μm. The corresponding contrast-to-noise ratio (CNR) values are provided in Supplementary Table S8.

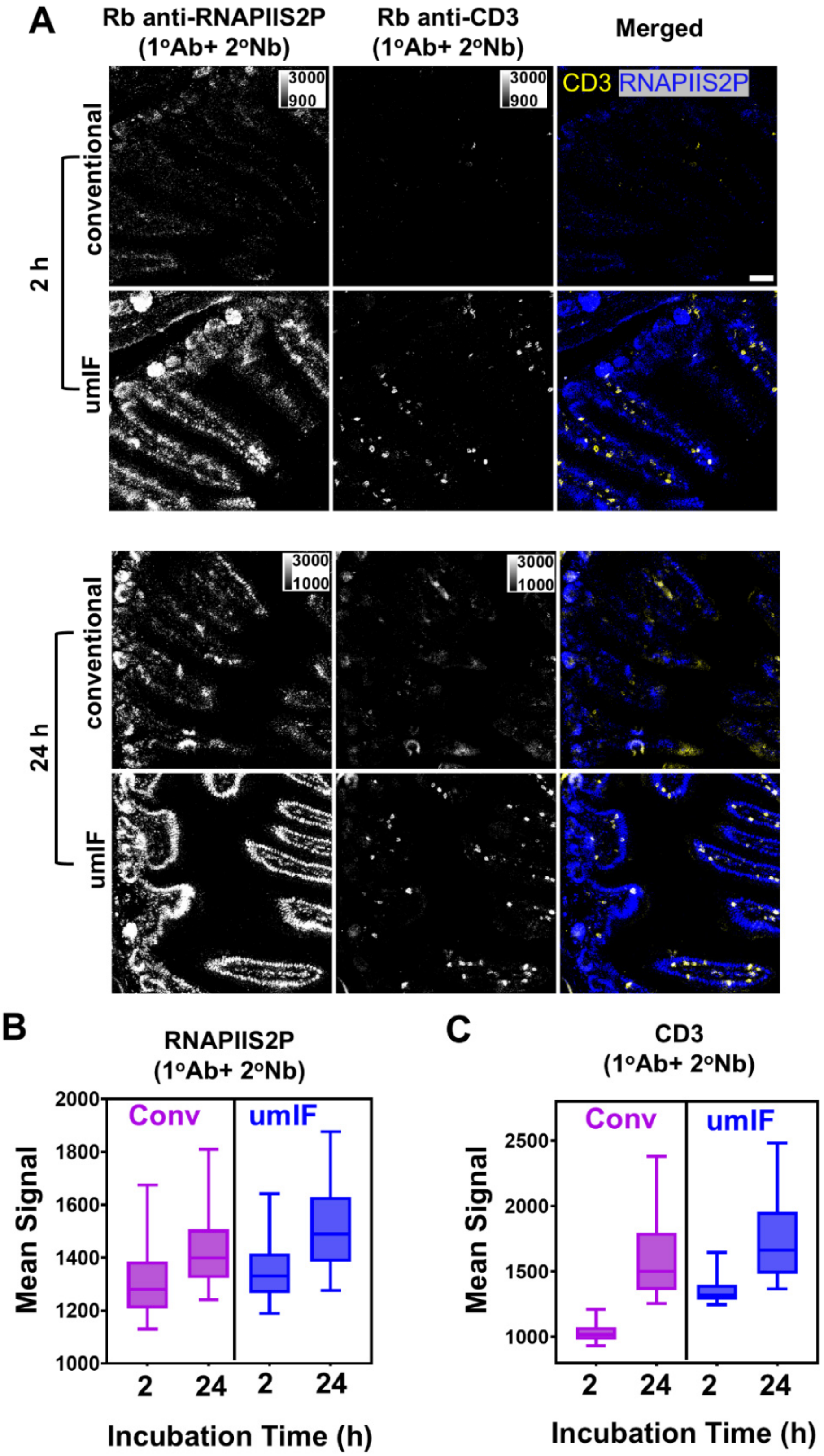

Supplementary Figure S10: ***umIF complex*** **improves labeling efficiency of same-host** ***complexes*** **(1°Ab + 2°Nb) compared with conventional immunofluorescence (conv) during extended incubation (3 hours and 24 hours).** (**A**) Representative images of mouse small intestine co-stained with same-host antibody-nanobody complexes targeting RNAPII S2P (blue) and CD3 (yellow) using conventional IF (conv, top) or umIF (bottom) after 3 hours (3h) or 24 h incubation. Conventional IF staining produced weaker and less uniform signals, whereas umIF yielded brighter and more homogeneous labeling across

both incubation conditions. (**B–C**) Quantification of mean fluorescence intensity for RNAPII S2P (B) and CD3 (C) at 3 h and 24 h incubation as shown in (A). Data are presented as box-and-whisker plots (5th–95th percentile). For RNAPII S2P, fluorescence intensities (mean ± SD, a.u.) were 1329.0 ± 5.0 and 1364.0 ± 2.1 after 2 h incubation, and 1448.0 ± 5.0 and 1524.0 ± 2.0 after 24 h incubation for conventional and umIF staining, respectively. For CD3, fluorescence intensities were 1045.0 ± 5.8 and 1624.0 ± 13.0 after 2 h incubation, and 1386.0 ± 13.6 and 1769.0 ± 16.6 after 24 h incubation for conventional and umIF staining, respectively. The number of segmented signals is ~500 – 6000. umIF consistently enhanced labeling intensity compared with conventional immunofluorescence, with a more pronounced improvement observed for the surface marker CD3. Scale bars, 50 μm.

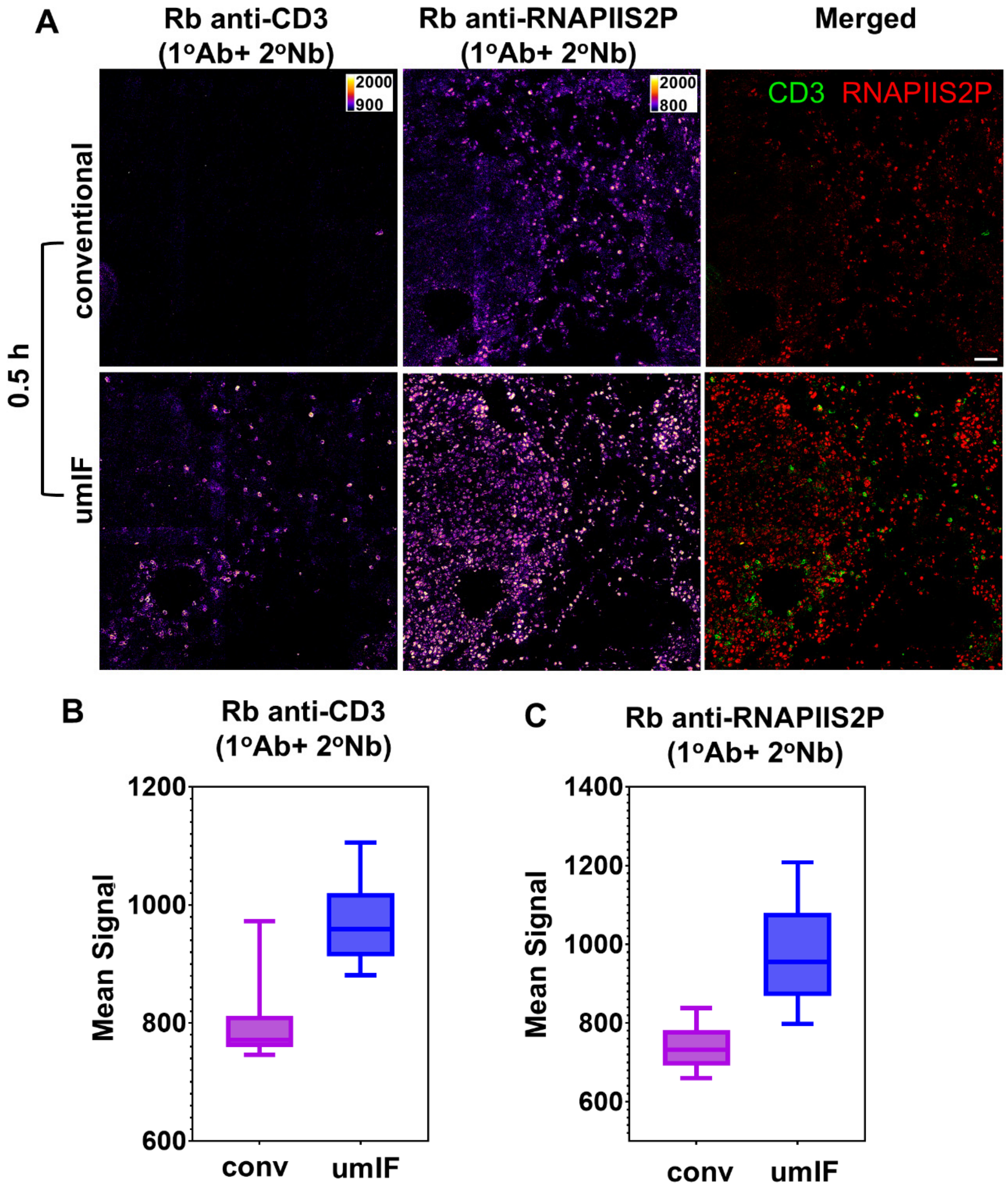

Supplementary Figure S11: umIF enables effective labeling of same-host *complexes* (1°Ab + 2°Nb) under short incubation condition (30 minutes).

(**A**) Representative images of lung tissue in *Kras*^{G12D}*Lkb1*$^{-/-}$ mouse model of non-small cell lung cancer (NSCLC), stained with rabbit-derived antibody-nanobody complexes targeting CD3 (green) and RNAPII S2P (red) using conventional IF (top) or umIF (bottom) after 30 minutes of incubation. Conventional staining produced rather weak and uneven labeling, whereas umIF yielded stronger signals for both markers. (**B–C**) Quantification of mean fluorescence intensity for CD3 (B) and RNAPII S2P (C) as shown in (A). Data are presented as box-and-whisker plots (5th-95th percentile). For CD3, fluorescence intensities (mean ± SD, a.u.) were 797.9 ± 22.4 and 982.0 ± 6.1 for conventional and umIF staining, respectively. For RNAPII S2P, fluorescence intensities were 742.8 ± 1.2 and 985.5 ± 3.4 for conventional and umIF staining, respectively. The number of segmented regions with positive signals is ~100 – 4000. Scale bars, 50 µm.

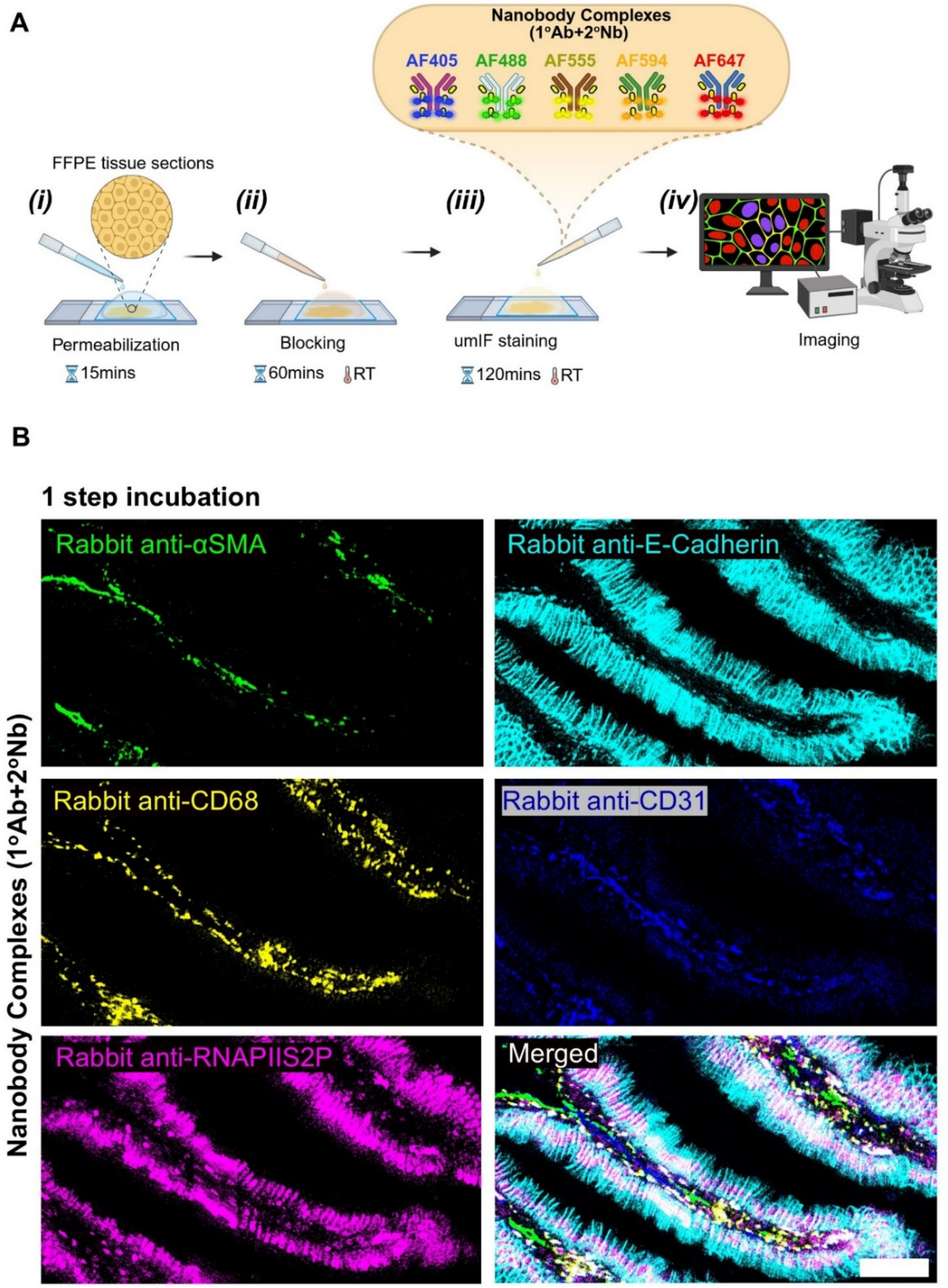

Supplementary Figure S12: Workflow and representative example of *umIF complexes* for one-step staining using five same-host antibody-nanobody complexes (1°Ab + 2°Nb).

(**A**) Schematic illustration of the umIF workflow. Formalin-fixed paraffin-embedded (FFPE) tissue sections were permeabilized *(i)*, serum-blocked *(ii)*, and incubated with five same-host antibody-nanobody complexes (1°Ab + 2°Nb), in which each primary antibody (1°Ab) was pre-mixed with a nanobody conjugated to spectrally distinct fluorophores (AF405, AF488, AF555, AF594, AF647), enabling multiplexed one-step staining *(iii)*, followed by imaging *(iv)*. (**B**) Representative umIF images of mouse small intestine after one-step incubation with rabbit-derived antibody-nanobody complexes against αSMA (green), E-cadherin (cyan), CD68 (yellow), CD31 (blue), and RNAPII S2P (magenta). Merged panels demonstrate simultaneous multiplexed detection of epithelial, stromal, vascular, immune, and nuclear compartments in a single staining step. Scale bars, 100 µm.

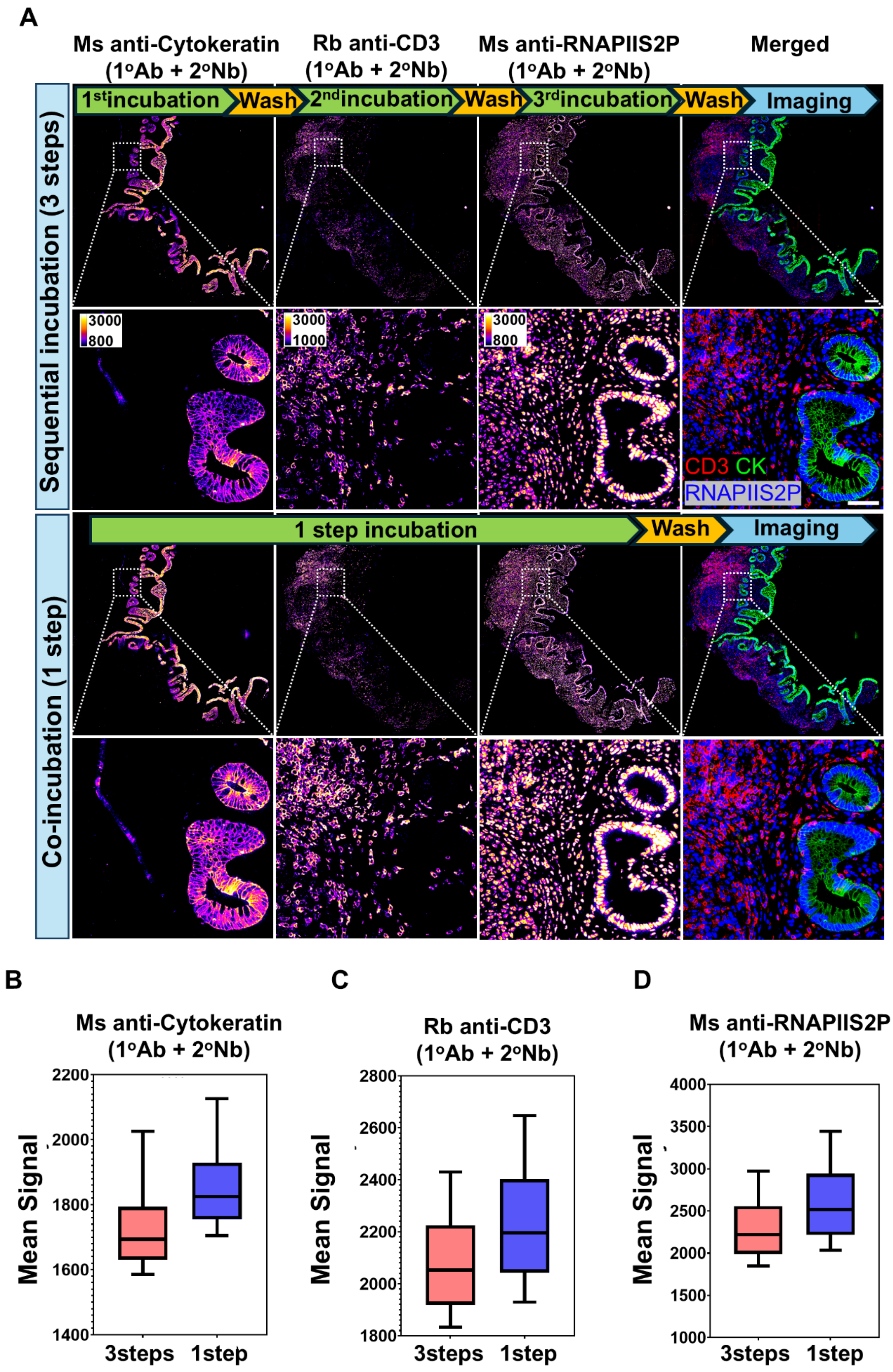

Supplementary Figure S13: Comparison of sequential versus co-incubation strategies for *umIF complexes* labeling.

(**A**) Representative images of an inflamed colon tissue (ulcerative colitis (UC)) stained with antibody-nanobody complexes targeting cytokeratin (CK, green), CD3 (red), and RNAPIIS2P (blue). The top panel

shows sequential incubation, in which each target was labeled individually, with washing after each incubation. The bottom panel shows co-incubation, where all three antibody-nanobody complexes were applied simultaneously in a single step. Sequential incubation required multiple rounds of incubation and washing, whereas co-incubation enabled efficient labeling in a single step. Merged panels demonstrate equivalent multiplexed detection of CK, CD3, and RNAPII-S2P with one-step umIF co-incubation. (**B–D**) Quantification of mean fluorescence intensity for cytokeratin (B), CD3 (C), and RNAPIIS2P (D) as shown in (A). Data are presented as box-and-whisker plots (5th–95th percentile). Data are presented as box-and-whisker plots (5th–95th percentile). For cytokeratin, fluorescence intensities (mean ± SD, a.u.) were 1801.0 ± 476.7 and 1910.0 ± 405.6 for sequential and one-step staining, respectively. For CD3, fluorescence intensity was 2106.0 ± 282.8 and 2265.0 ± 338.2, respectively. For RNAPII S2P, fluorescence intensity was 2327.0 ± 455.7 and 2646.0 ± 577.3, respectively. The number of segmented regions with positive signals is ~1000 – 10000. One-step co-incubation produced comparable or higher labeling intensity relative to sequential staining while substantially simplifying the multiplexed staining workflow. Scale bars, 200 µm.

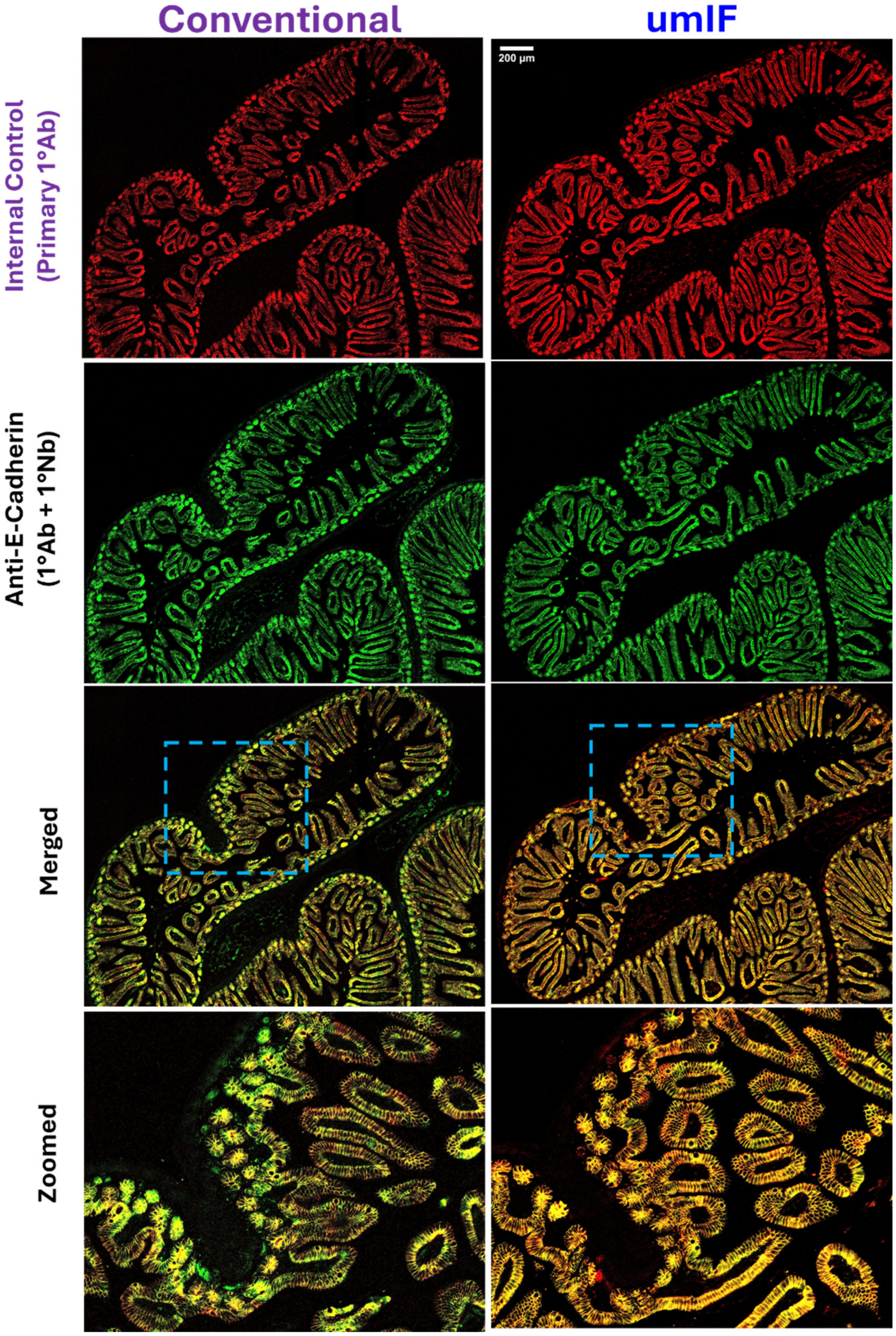

Supplementary Figure S14: Comparison of conventional staining and umIF for anti-E-cadherin labeling with antibody–nanobody complexes in tissue sections, using a direct-label internal control. Left, conventional staining; right, umIF. The top row shows the internal control signal from direct dye-conjugated anti-E-cadherin primary antibodies (red; primary 1° Ab), the second row shows the immunofluorescence signal from antibody–nanobody complexes (green; 1° Ab + 2° Nb), the third row shows the merged images, and the bottom row shows magnified views of the regions outlined by the blue boxes. Scale bar, 200 µm. Compared with conventional staining, umIF shows stronger spatial concordance between the internal control and anti-E-cadherin signals, as reflected by the higher Pearson correlation coefficient in the umIF condition (0.85 versus 0.65).

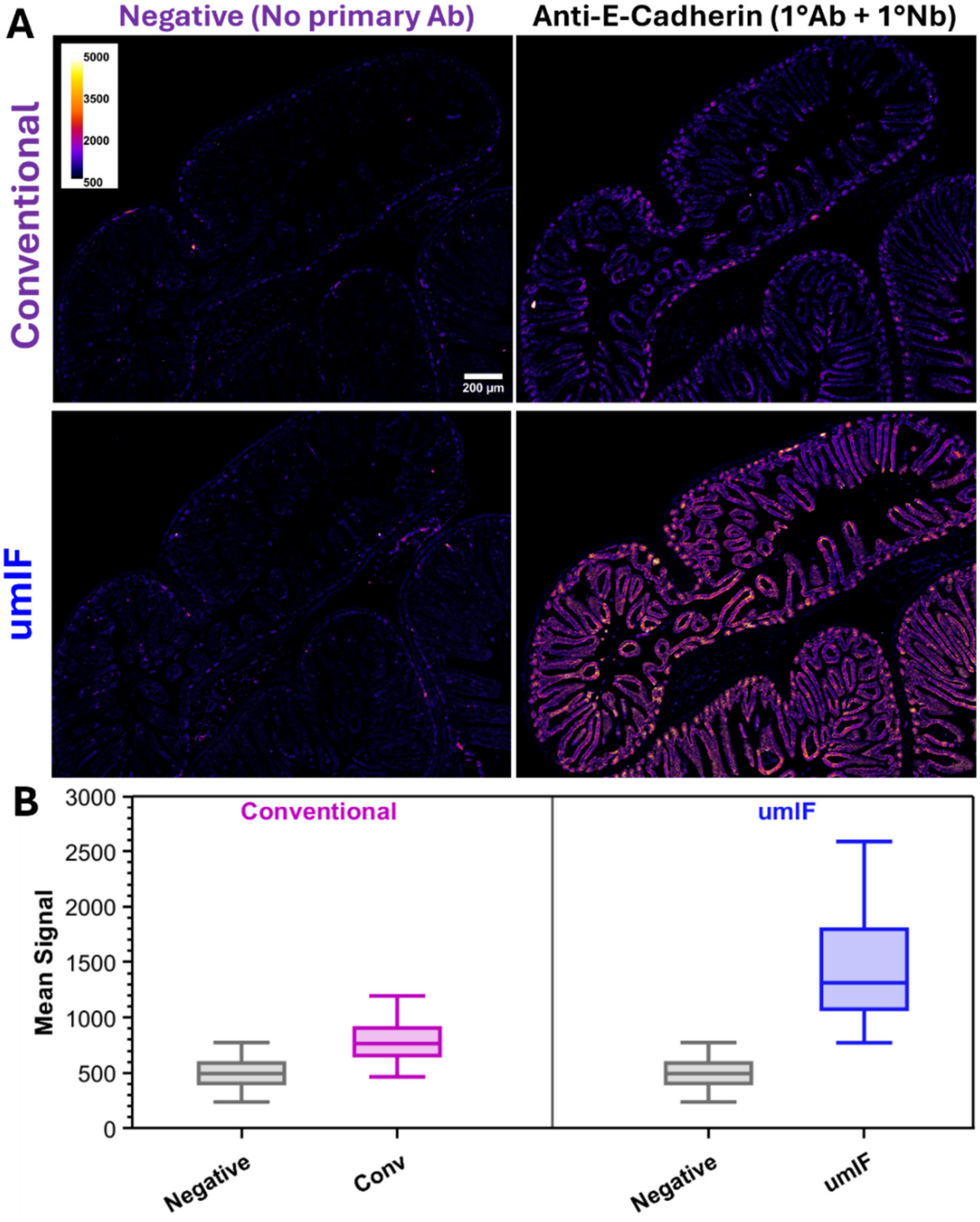

Supplementary Figure S15: Negative control analysis for anti-E-cadherin labeling using antibody-nanobody complexes in tissue sections under conventional and umIF conditions.
(**A**) Representative images of tissue sections acquired under conventional staining (top row) and umIF (bottom row). In each row, the left panel shows the negative control without primary antibody (secondary nanobody present), and the right panel shows the corresponding immunofluorescence image obtained with antibody-nanobody complexes. The negative controls show low residual background in both conditions, whereas the stained samples exhibit clear epithelial labeling, with higher signal intensity in the umIF condition. Scale bar, 200 µm. (**B**) Quantification of mean signal intensity, presented as box-and-whisker plots (5th–95th percentile), for the negative controls and corresponding stained samples under conventional and umIF conditions. In both cases, the stained samples exhibited substantially higher signal than the no-primary-antibody controls, confirming staining specificity. Compared with conventional staining, umIF produced higher signal intensity while maintaining low background in the negative controls). Data are presented as box-and-whisker plots showing the 5th–95th percentile. Fluorescence intensities, reported as mean ± SD in arbitrary units (a.u.), were 502.3 ± 200.5 for the negative control, 796.6 ± 352.4 for conventional IF, and 1484.0 ± 852.6 for umIF. The number of segmented regions with positive signals is ~200 - 2200.

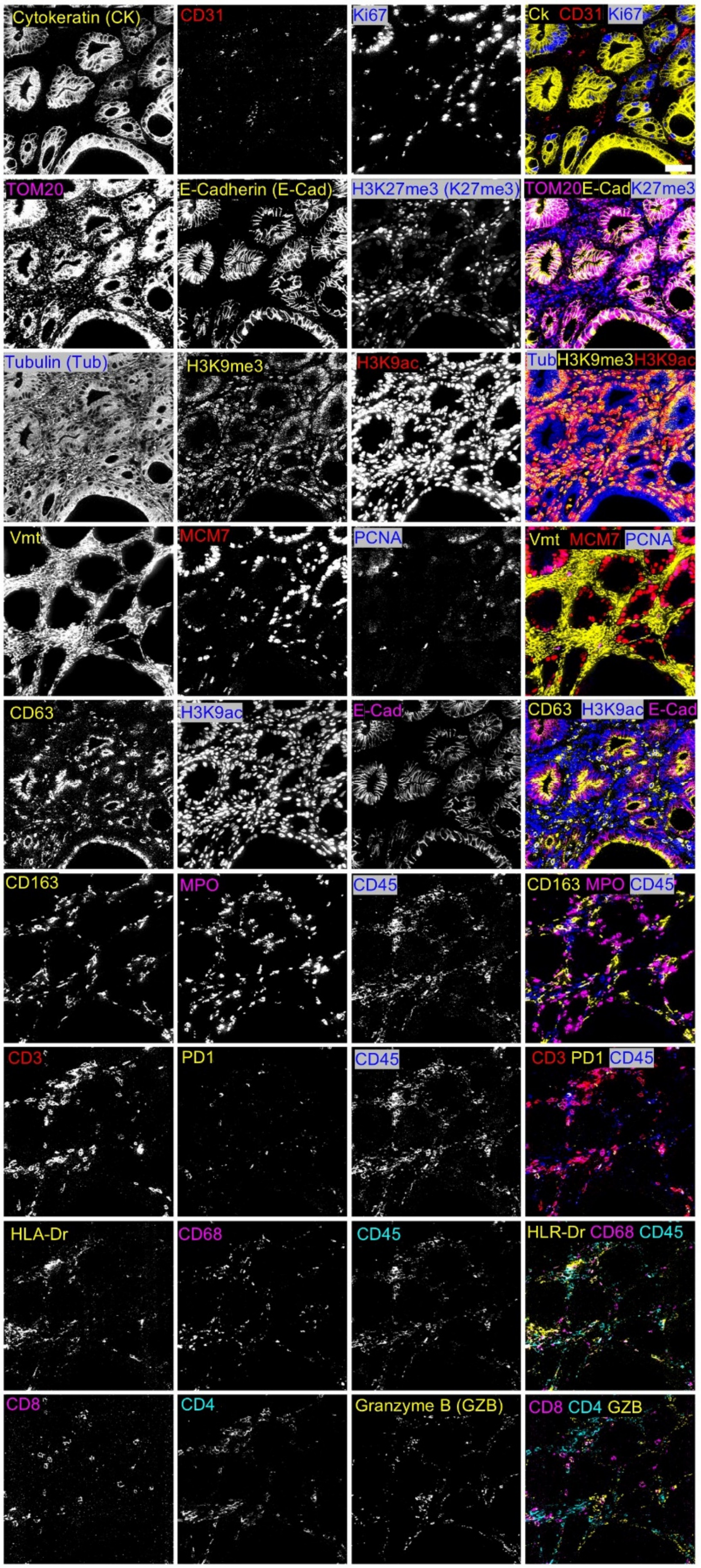

Supplementary Figure S16: Representative single-channel and merged umIF images demonstrating multiplex staining in a colon tumor from a patient with advanced adenoma processed through 10 iterative umIF cycles.

Each panel shows individual single-channel image (grayscale) and corresponding merged views for epithelial, cytoplasmic, stromal, and immune markers, including TOM20, CD63, MPO, E-cadherin, Cytokeratin, Vimentin (Vmt), CD31, αSMA, Ki67, MCM7, Fox-P3, H3K27me3, H3K4me3, H3K9me3, H3K9ac, PCNA, HLA-DR, CD45, CD3, CD4, CD8, CD68, CD163, PD1, and Granzyme B (GZB). Scale bars, 50 μm.

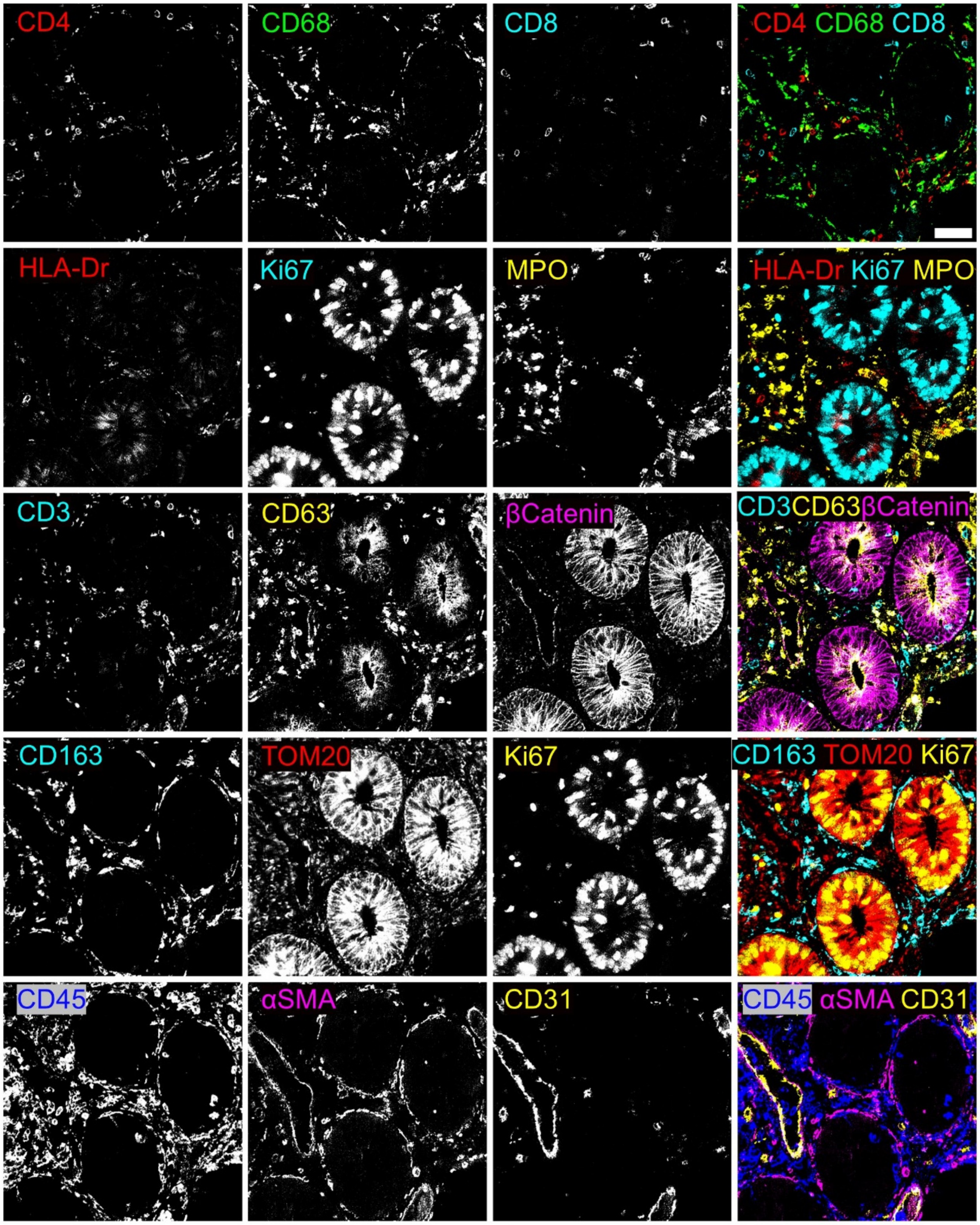

Supplementary Figure S17: Representative single-channel and merged umIF images of a human ulcerative colitis (UC) tissue.

Single-channel (grayscale) and merged images demonstrate spatial distribution of different cell populations including immune, stromal, epithelial, and proliferative markers in the Region 1 of Fig. 6B in the main text. The multiplex antibody panel included HLA-DR, Ki67, β-catenin, alpha-smooth muscle actin (αSMA), CD31, CD3, CD4, CD8, CD45, CD68, CD163, MPO, and TOM20. Scale bar, 50 µm.

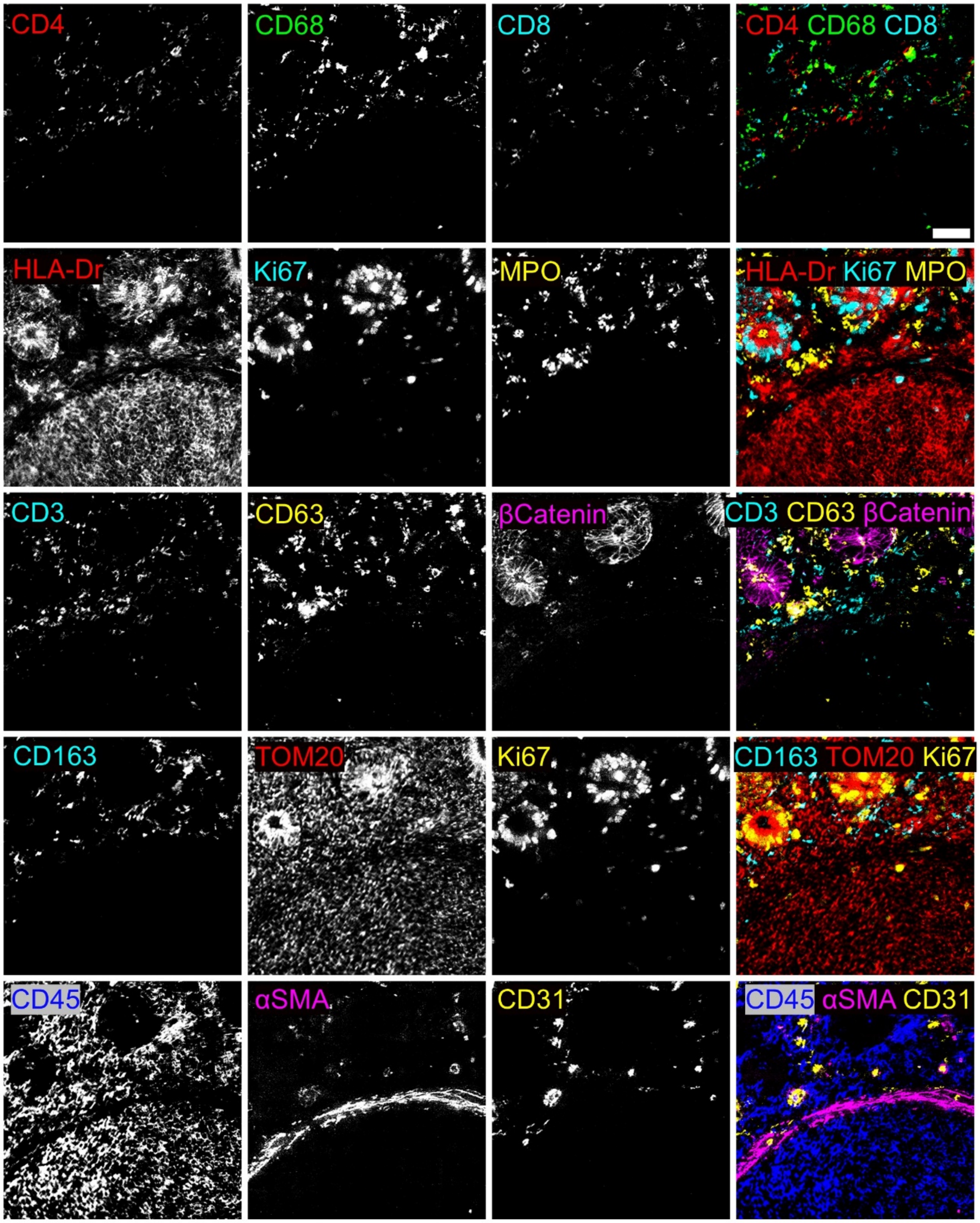

Supplementary Figure S18: Representative single-channel and merged umIF images of human ulcerative colitis (UC) tissue.

Single-channel (grayscale) and merged images show spatial distribution of different cell populations, including immune, stromal, epithelial, and proliferative markers in the Region 2 of Fig. 6B in the main text. Scale bar, 50 µm.

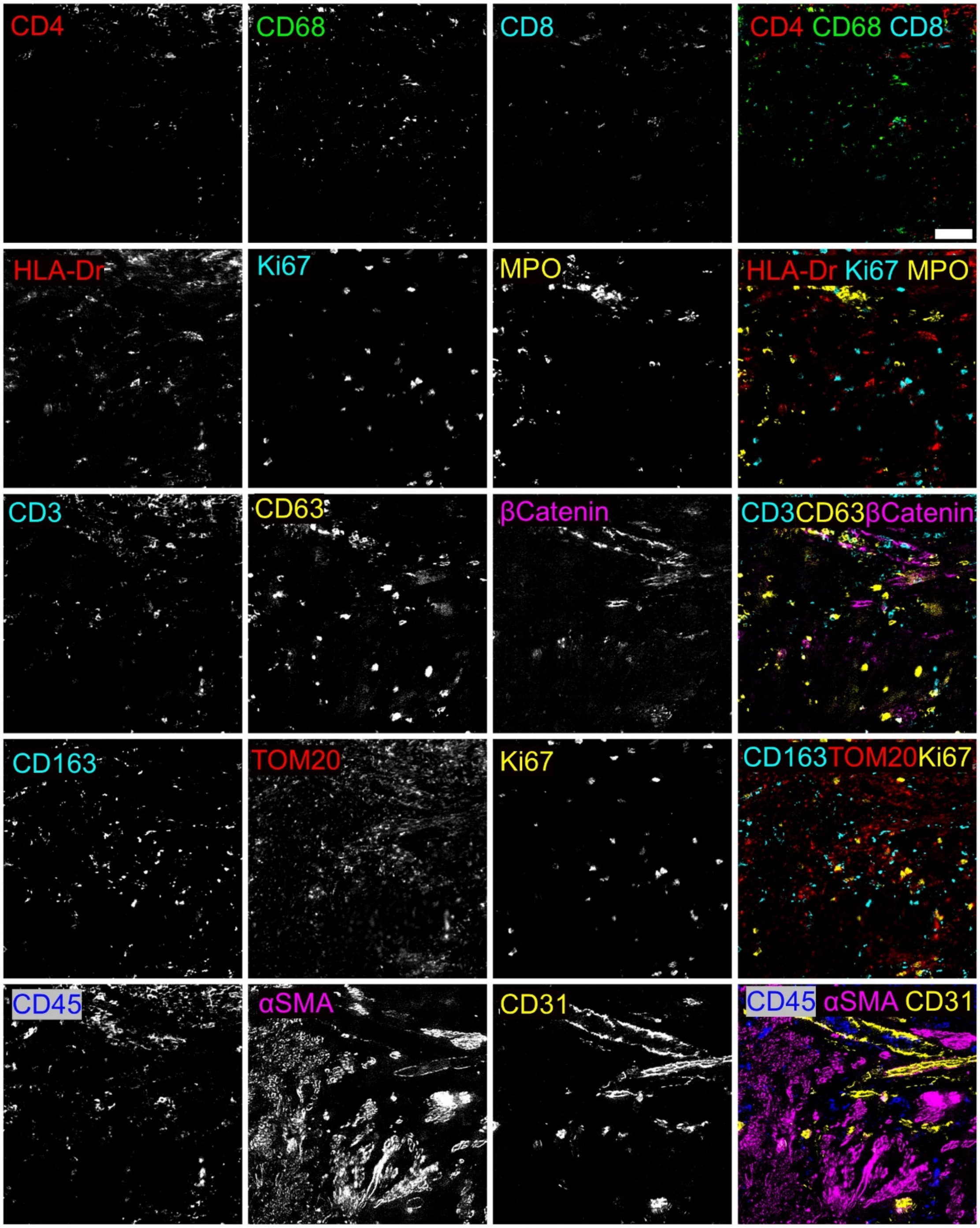

Supplementary Figure S19: Representative single-channel and merged umIF images of human ulcerative colitis (UC) tissue.

Single-channel (grayscale) and merged images show the spatial distribution of different cell populations and tissue compartments, showing immune, stromal, epithelial, and proliferative markers in the Region 3 of Fig. 6B in the main text. Scale bar, 50 µm.

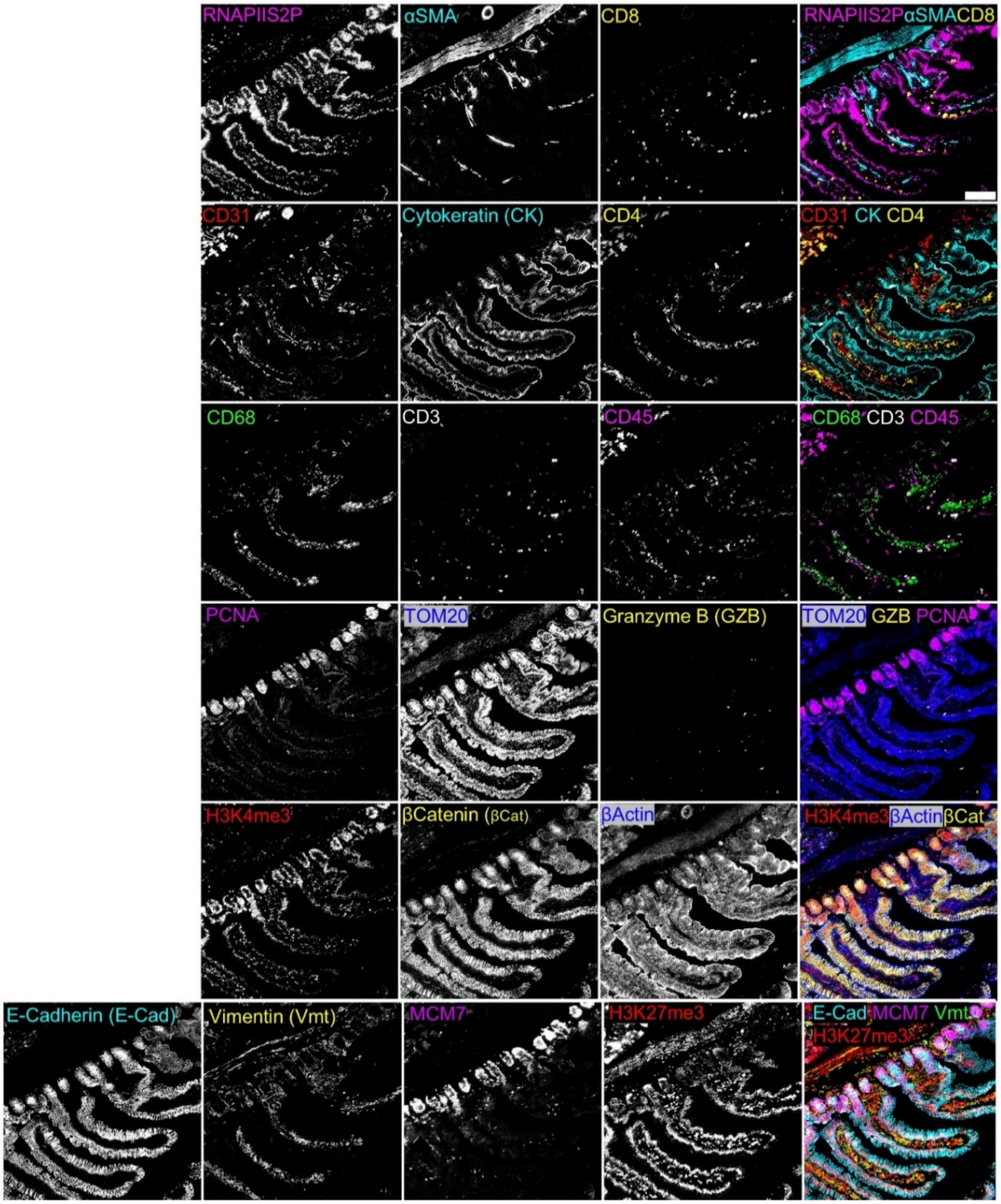

Supplementary Figure S20: Representative single-channel and merged umIF images of mouse small intestine across 10 cycles.

Single-channel (grayscale) and merged images show spatial distribution of epithelial, stromal, immune cells and nuclear markers. The multiplex antibody panel included E-cadherin, cytokeratin (CK), β-catenin, PCNA, MCM7, vimentin (Vmt), αSMA, CD31, TOM20, RNAPII S2P, H3K27me3, H3K4me3, H3K9me3, CD3, CD4, CD8, CD45, CD68, PD1, and Granzyme B (GZB). Scale bar, 50 μm.

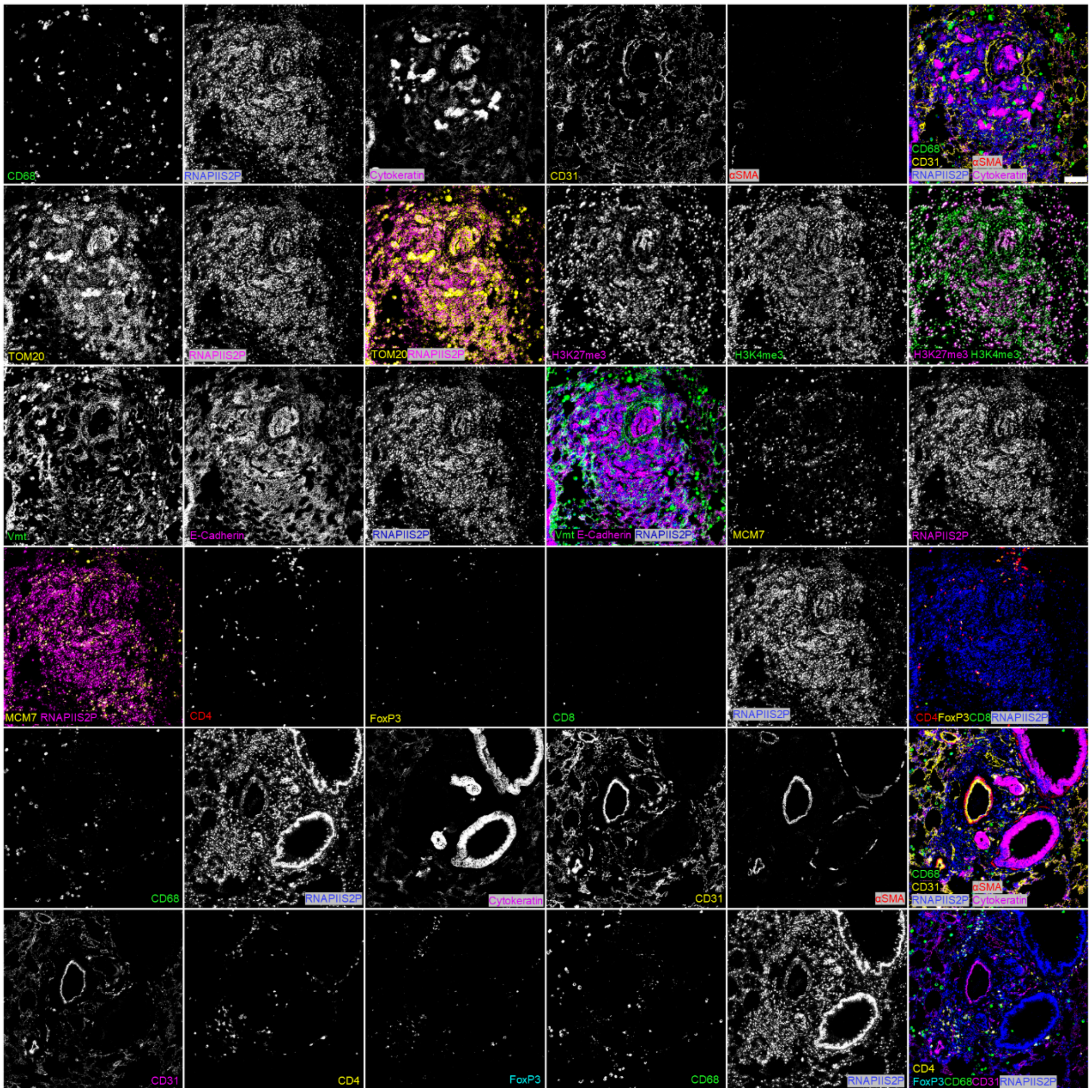

Supplementary Figure S21: Representative single-channel and merged umIF images of lung tissue from *Kras*^{G12D}*Lkb1*$^{-/-}$ mouse model across multiplexed cycles.

Single-channel (grayscale) and merged images highlight the spatial organization of epithelial, stromal, immune, proliferative, and transcriptional compartments within the NSCLC tumor microenvironment. The multiplex antibody panel included E-cadherin, cytokeratin, MCM7, vimentin (Vmt), αSMA, CD31, TOM20, H3K27me3 and H3K4me3, CD8, CD68, CD4, and FoxP3. Scale bar, 100 µm.

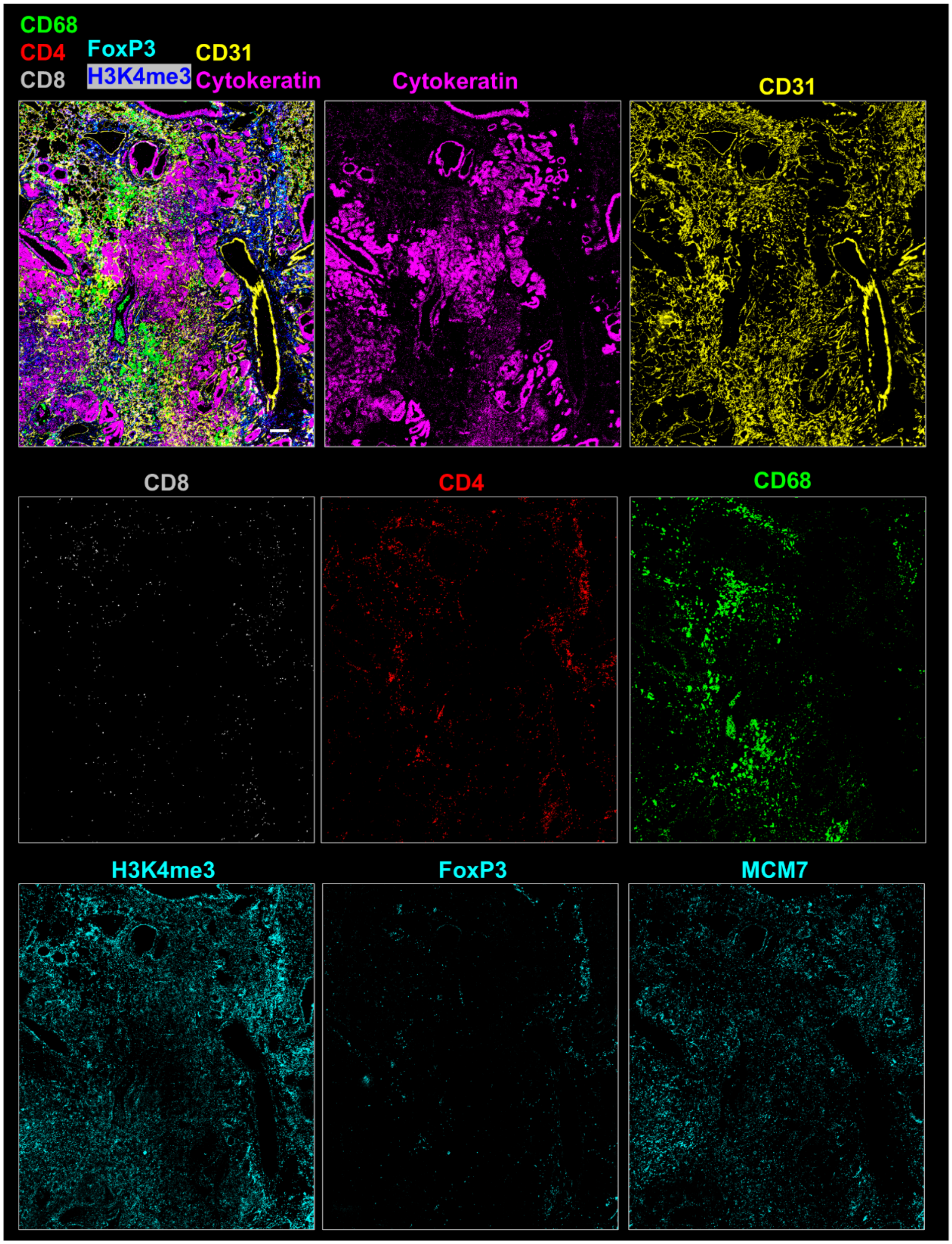

Supplementary Figure S22: Representative single-channel and merged umIF images for populations in *Kras*^{G12D}*Lkb1*$^{-/-}$ mouse model.

Single-channel and merged images highlight the spatial organization of epithelial, stromal, immune, proliferative, and histone markers within *Kras*^{G12D}*Lkb1*$^{-/-}$ tumor microenvironment. The multiplex antibody panel included cytokeratin, CD31, MCM7, H3K4me3, CD8, CD68, CD4, and FoxP3. Scale bar, 200 μm.

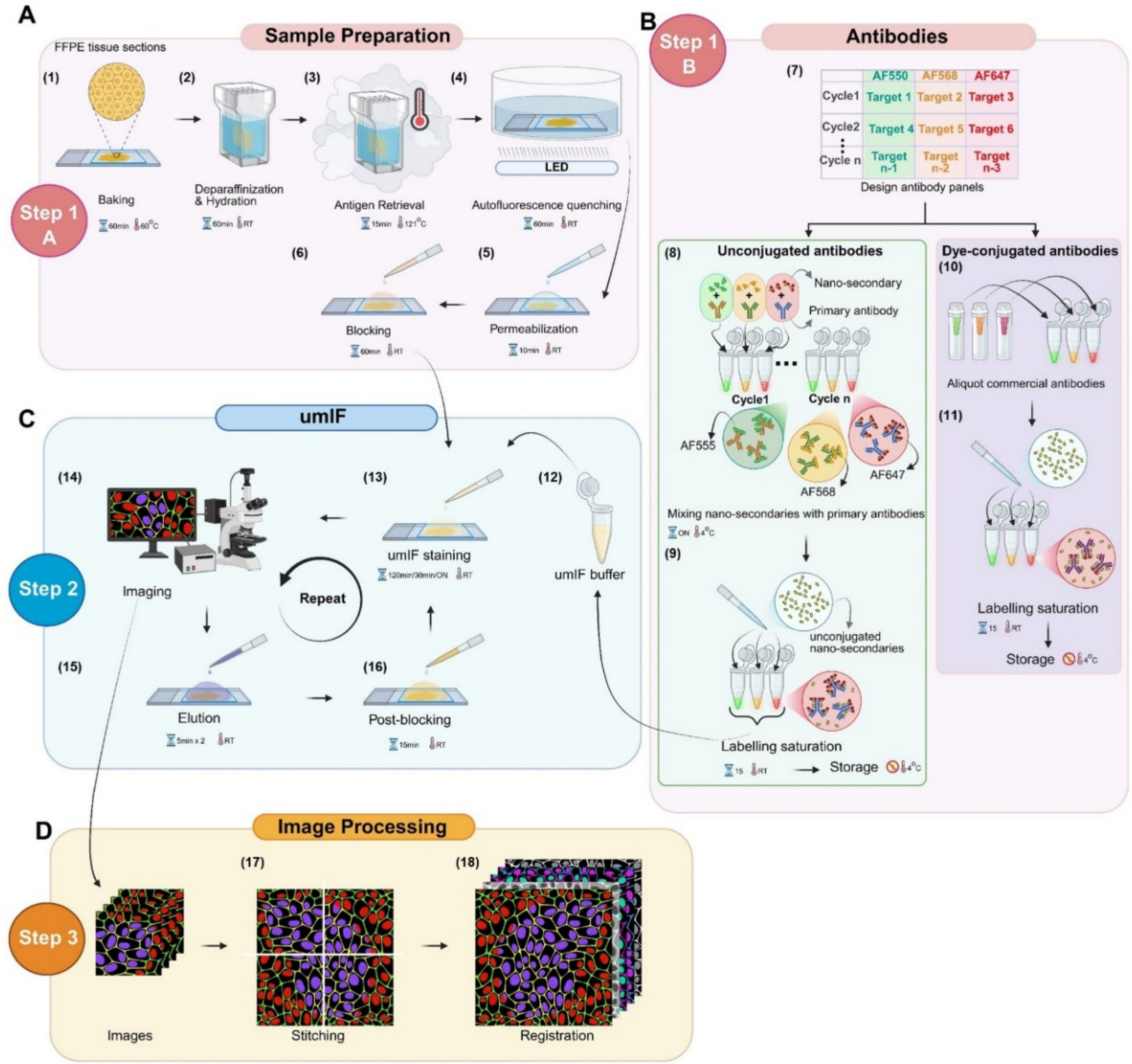

Supplementary Figure S23: General workflow for universal multiplexed immunofluorescence (umIF) on FFPE tissue sections.

(**A**) **Sample preparation:** FFPE tissue sections undergo (1) baking, (2) deparaffinization and hydration, (3) antigen retrieval, followed by (4) autofluorescence quenching and (5) permeabilization, followed by (6) serum blocking.
(**B**) **Antibody preparation:** (7) design antibody panels, (8) unconjugated primary antibodies are pre-mixed with dye-conjugated secondary nanobodies (1°Ab + 2°Nb) to form antibody-nanobody complexes, (9) with excess unlabeled nanobodies used to saturate Fc sites. (10) Dye-conjugated primary antibodies undergo (11) the same Fc-blocking process before (12) being pooled with antibody-nanobody complexes in umIF buffer.
(**C**) **umIF workflow:** (13) multiplex immunofluorescence staining (as short as 30 min or extended time), (14) imaging, (15) rapid elution, and (16) post-blocking steps can be repeated iteratively across multiple cycles for expanded target coverage.
(**D**) **Image processing:** acquired images undergo (17) stitching, (18) registration and segmentation to enable downstream quantitative analyses.

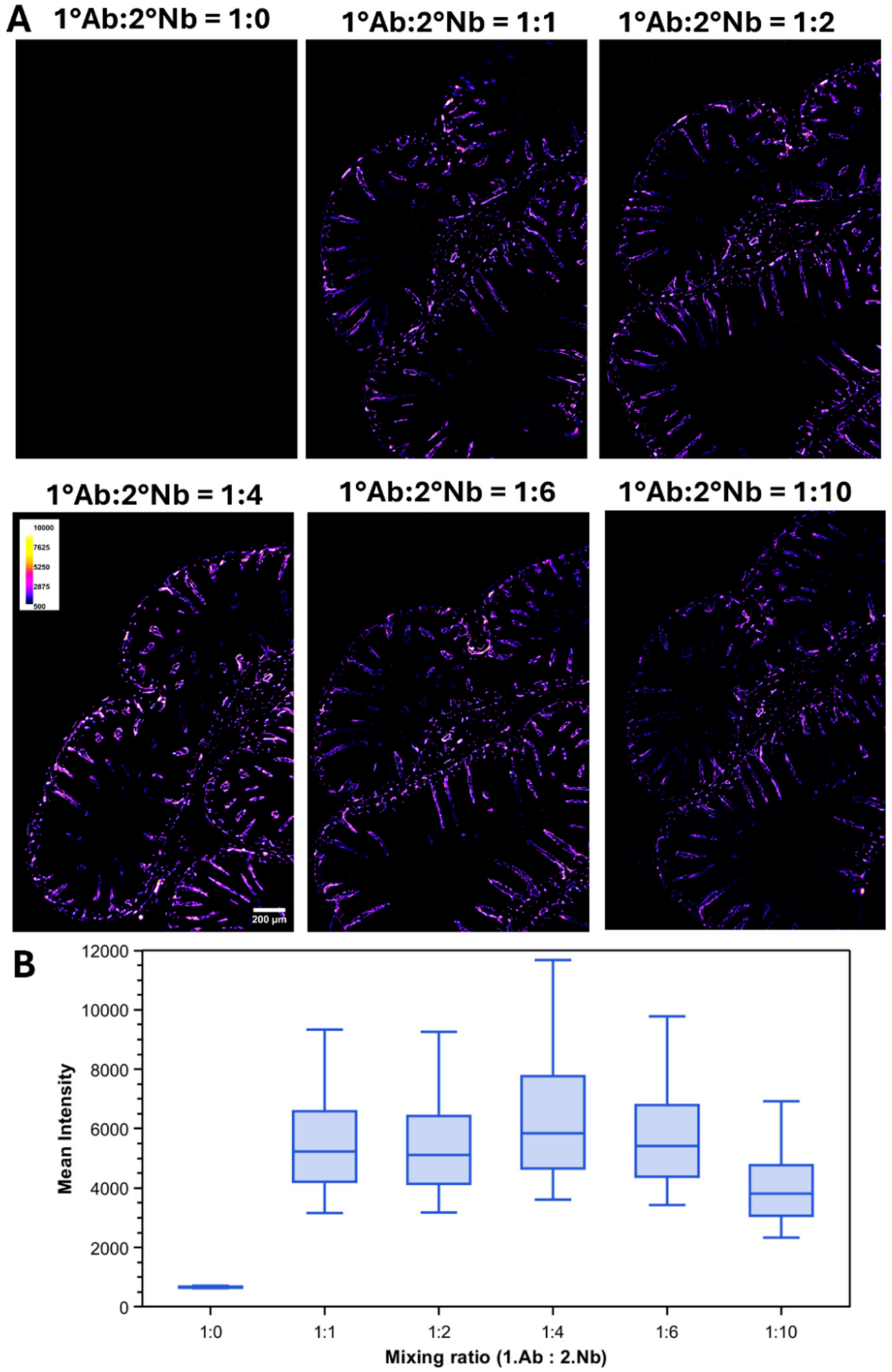

Supplementary Figure S24: Effect of the mixing ratio between primary antibody (1° Ab) and secondary nanobody (2° Nb) on CD31 staining intensity. (**A**) Representative images show CD31 labeling obtained with primary antibody only (1:0) and with 1°Ab:2°Nb mixing ratios of 1:1, 1:2, 1:4, 1:6, and 1:10. All images are displayed using the same intensity range. (**B**) The boxplot quantifies mean signal intensity for each condition, shown from the 5th to 95th percentiles. Fluorescence intensities, reported as mean ± SD in arbitrary units (a.u.), were 664.9 ± 25.7, 5610.4 ± 1967.0, 5524.2 ± 1994.8, 6654.0 ± 2695.2, 5866.6 ± 2108.1, and 4103.7 ± 1506.6 for primary antibody-to-secondary nanobody mixing ratios of 1:0, 1:1, 1:2, 1:4, 1:6, and 1:10, respectively. Quantification was performed using ~3000–5000 segmented regions with positive signals. Compared with the primary-antibody-only condition, antibody–nanobody complex formation markedly increased the CD31 signal, with the highest intensity observed at the 1:4 mixing ratio under these conditions. Scale bar, 200 µm.

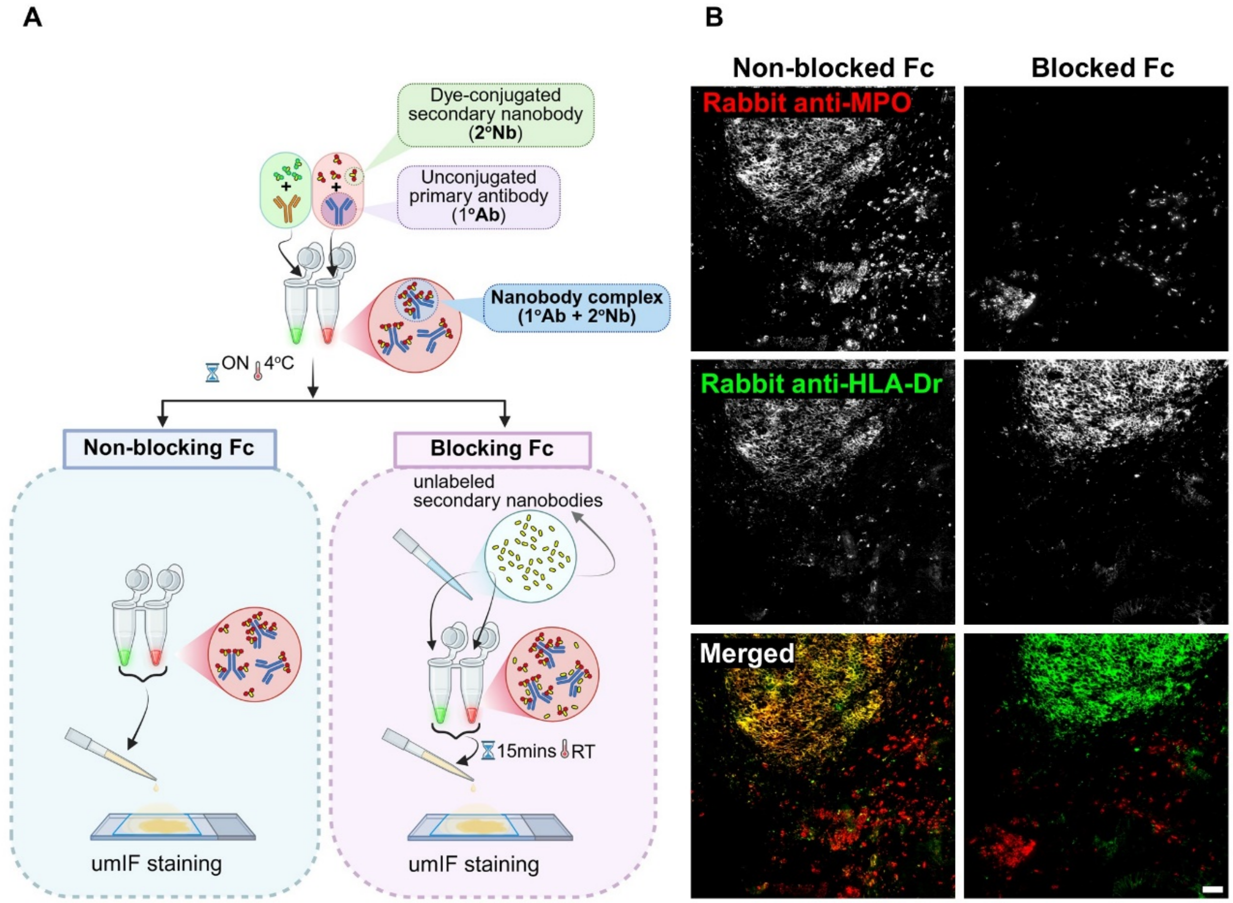

Supplementary Figure S25: Cross-binding elimination by Fc blocking on primary antibodies.

(**A**) Schematic workflow illustrates the saturation of residual Fc sites on unconjugated primary antibodies to minimize cross-binding during umIF staining. Non-blocked Fc sites (left) allow unintended binding of unbound secondary nanobodies, whereas pre-incubation with concentrated unlabeled secondary nanobodies efficiently blocks Fc sites (right), reducing nonspecific interactions.

(**B**) Representative umIF images of human ulcerative colitis (UC) tissue stained with rabbit anti-MPO (red) and rabbit anti-HLA-DR (green) under non-blocked (left) or blocked Fc conditions (right). Blocking Fc sites markedly reduced cross-binding and improved signal specificity. Merged panels show reduced overlap and clearer separation of target signals with Fc blocking. Scale bars, 50 µm.
