# Supplementary material for: A Versatile Multiplexed Immunofluorescence Strategy for Efficient, Host‐Independent, and Scalable Spatial Protein Profiling
Source: Small Methods. 2026 Jun 17;10(14):e00009. doi: 10.1002/smtd.202600009 (PMC13375603; doi:10.1002/smtd.202600009)
Supplement: Supplementary file 2 — Supporting File 2: smtd70783‐sup‐0002‐TableS1‐S21.pdf. [file SMTD-10-e00009-s002.pdf]

Supplementary Tables

Supplementary Table S1. Comparison of signal accumulation during antibody incubation (anti-CD19 1° Ab) under Conventional (Conv) and umIF Conditions (Figure 2)

Assay condition	Signal (mean ± SD)	CNR (mean ± SD)
**0 minutes (Conv)**	182 ± 26	0.1 ± 0.2
**0 minutes (umIF)**	247 ± 40	0.2 ± 0.2
**30 minutes (Conv)**	381 ± 168	1.2 ± 1.0
**30 minutes (umIF)**	1212 ± 236	5.5 ± 1.3
**2 hours (Conv)**	619 ± 176	2.8 ± 1.1
**2 hours (umIF)**	1316 ± 298	5.6 ± 1.5
**24 hours (Conv)**	965 ± 183	5.2 ± 1.2
**24 hours (umIF)**	2148 ± 422	9.2 ± 2.0

Supplementary Table S2. Comparison of signal accumulation during antibody incubation (anti-PCNA 1° Ab) under Conventional (Conv) and umIF Conditions (Figure 2)

Assay condition	Signal (mean ± SD)	CNR (mean ± SD)
**30 minutes (Conv)**	487 ± 152	2.4 ± 1.1
**30 minutes (umIF)**	791 ± 242	4.3 ± 1.6
**2 hours (Conv)**	1074 ± 353	7.2 ± 2.6
**2 hours (umIF)**	1937 ± 618	10.5 ± 3.7
**24 hours (Conv)**	1027 ± 416	7.0 ± 3.1
**24 hours (umIF)**	3906 ± 1511	15.1 ± 6.1
**48 hours (Conv)**	2926 ± 1125	14.2 ± 5.7
**48 hours (umIF)**	4378 ± 1784	14.5 ± 6.2

Supplementary Table S3. Comparison of signal accumulation during antibody incubation (anti-E-Cadherin 1° Ab) under Conventional (Conv) and umIF Conditions (Supplementary Figure S4)

Assay condition	Signal (mean ± SD)	CNR (mean ± SD)
**30 minutes (Conv)**	1155 ± 443	4.1 ± 1.9
**30 minutes (umIF)**	2362 ± 803	5.2 ± 2.0
**3 hours (Conv)**	3501 ± 1330	6.2 ± 2.5
**3 hours (umIF)**	5372 ± 1774	6.0 ± 2.1
**24 hours (Conv)**	8689 ± 2641	7.0 ± 2.2
**24 hours (umIF)**	10940 ± 3418	6.4 ± 2.1

Supplementary Table S4. Comparison of signal accumulation during antibody incubation (anti-E-Caderin antibody-nanobody complexes 1°Ab + 2°Nb) under Conv and umIF Conditions (Supplementary Figure S5)

Assay condition	Signal (mean ± SD)	CNR (mean ± SD)
**30 minutes (Conv)**	495 ± 149	1.4 ± 0.7
**30 minutes (umIF)**	910 ± 247	3.2 ± 1.1
**3 hours (Conv)**	1059 ± 237	4.0 ± 1.1
**3 hours (umIF)**	1862 ± 545	4.9 ± 1.7
**24 hours (Conv)**	3062 ± 603	6.1 ± 1.3
**24 hours (umIF)**	4831 ± 1081	6.4 ± 1.6

Supplementary Table S5. Comparison of signal accumulation during antibody incubation (anti-PCNA antibody-nanobody complexes 1°Ab + 2°Nb) under Conv and umIF Conditions (Supplementary Figure S6)

Assay condition	Signal (mean ± SD)	CNR (mean ± SD)
**30 minutes (Conv)**	411 ± 197	1.1 ± 1.0
**30 minutes (umIF)**	509 ± 305	2.0 ± 1.8
**2 hours (Conv)**	479 ± 213	1.8 ± 1.3
**2 hours (umIF)**	542 ± 351	2.5 ± 2.3
**24 hours (Conv)**	883 ± 191	4.7 ± 1.3
**24 hours (umIF)**	1288 ± 412	7.1 ± 2.6
**48 hours (Conv)**	1152 ± 202	6.1 ± 1.3
**48 hours (umIF)**	2190 ± 556	9.1 ± 2.5

Supplementary Table S6. Comparison of signal retention after anti-E-Caderin 1°Ab labeling under Conv and umIF Conditions (Supplementary Figure S7)

Assay condition	Signal (mean ± SD)	CNR (mean ± SD)
**0 minutes (Conv)**	8706 ± 2650	7.0 ± 2.2
**0 minutes (umIF)**	10939 ± 3410	6.4 ± 2.1
**3 hours (Conv)**	7486 ± 2448	6.8 ± 2.4
**3 hours (umIF)**	9312 ± 3022	6.3 ± 2.2
**24 hours (Conv)**	5324 ± 2722	6.1 ± 3.3
**24 hours (umIF)**	7729 ± 2575	6.2 ± 2.2
**96 hours (Conv)**	5456 ± 1792	6.7 ± 2.3
**96 hours (umIF)**	6168 ± 1990	6.2 ± 2.1

Supplementary Table S7. Comparison of signal retention after anti-E-Caderin antibody-nanobody complexes (1°Ab + 2°Nb) labeling under Conv and umIF Conditions (Supplementary Figure S8)

Assay condition	Signal (mean ± SD)	CNR (mean ± SD)
**0 minutes (Conv)**	3085 ± 601	6.0 ± 1.3
**0 minutes (umIF)**	4831 ± 1086	6.4 ± 1.6
**30 minutes (Conv)**	2045 ± 452	5.5 ± 1.4
**30 minutes (umIF)**	3574 ± 838	5.6 ± 1.5
**24 hours (Conv)**	1434 ± 332	4.5 ± 1.2
**24 hours (umIF)**	2874 ± 680	5.6 ± 1.5
**72 hours (Conv)**	1143 ± 289	3.7 ± 1.2
**72 hours (umIF)**	2315 ± 558	5.4 ± 1.5
**96 hours (Conv)**	1085 ± 289	3.5 ± 1.2
**96 hours (umIF)**	2067 ± 492	5.2 ± 1.4

Supplementary Table S8. Comparison of signal retention after anti-CD19 1°Ab labeling under Conv and umIF Conditions (Supplementary Figure S9)

Assay condition	Signal (mean ± SD)	CNR (mean ± SD)
**0 minutes (Conv)**	879 ± 177	4.2 ± 1.1
**0 minutes (umIF)**	2138 ± 426	8.7 ± 1.9
**45 minutes (Conv)**	582 ± 148	2.7 ± 1.0
**45 minutes (umIF)**	1201 ± 274	5.8 ± 1.6
**3 hours (Conv)**	444 ± 141	1.8 ± 0.9
**3 hours (umIF)**	817 ± 201	3.8 ± 1.2
**48 hours (Conv)**	253 ± 135	0.5 ± 0.9
**48 hours (umIF)**	448 ± 103	1.8 ± 0.7

Supplementary Table S9. Comparison of signal retention after anti-PCNA antibody-nanobody complexes (1°Ab + 2°Nb) labeling under Conv and umIF Conditions

Assay condition	Signal (mean ± SD)	CNR (mean ± SD)
**0 minutes (Conv)**	731 ± 159	3.7 ± 1.0
**0 minutes (umIF)**	1172 ± 313	5.7 ± 1.8
**3 hours (Conv)**	433 ± 148	2.1 ± 1.1
**3 hours (umIF)**	977 ± 343	4.8 ± 2.1

Supplementary Table S10. Comparison of signal accumulation during incubation for simultaneous antibody–nanobody complex staining of RNAPII-S2P, HLA-DR, and CD4 under conventional and umIF conditions (Figure 3)

	Assay condition	Signal (mean ± SD)	CNR (mean ± SD)
RNAPIIS2P	45 minutes (Conv)	777 ± 110	0.9 ± 0.4
	45 minutes (umIF)	1206 ± 172	1.7 ± 0.6
	3 hours (Conv)	2560 ± 512	2.4 ± 0.8
	3 hours (umIF)	2740 ± 528	3.0 ± 1.0
HLA-Dr	45 minutes (Conv)	755 ± 139	1.5 ± 0.5
	45 minutes (umIF)	1160 ± 231	2.5 ± 0.8
	3 hours (Conv)	2632 ± 703	2.9 ± 1.2
	3 hours (umIF)	3532 ± 765	3.6 ± 1.1
CD4	45 minutes (Conv)	754 ± 82	0.5 ± 0.2
	45 minutes (umIF)	1413 ± 252	2.0 ± 0.6
	3 hours (Conv)	1550 ± 276	0.7 ± 0.3
	3 hours (umIF)	4457 ± 884	3.5 ± 1.0

Supplementary Table S11. Comparison of 2-step IgG (1°Ab → 2°Ab) for FoxP3 under conventional and umIF buffer conditions (Figure 4A)

Assay condition	Signal (mean ± SD)	CNR (mean ± SD)
2-step IgG Ab (Conv)	1600 ± 642	10.1 ± 4.9
2-step IgG Ab (umIF)	1860 ± 778	12.8 ± 6.2

Supplementary Table S12. Comparison of 2-step IgG (1°Ab → 2°Ab), 2-step nanobody (Nb, 1°Ab → 2°Nb) and 1-step antibody-nanobody complexes (NbC, 1°Ab + 2°Nb) for RNAPIIS5P under conventional and umIF buffer conditions (Figure 4B)

Assay condition	Signal (mean ± SD)	CNR (mean ± SD)
2-step Nb (Conv)	15448 ± 367	10.7 ± 2.4
2-step Nb (umIF)	16833 ± 1066	10.2 ± 3.4
1-step NbC (Conv)	6936 ± 452	9.1 ± 2.5
1-step NbC (umIF)	12176 ± 724	9.9 ± 2.6

Supplementary Table S13. Comparison of 2-step IgG, 2-step nanobody (Nb) and 1-step antibody-nanobody complexes (NbC) for CD4 under conventional and umIF buffer conditions (Figure 4C)

Assay condition	Signal (mean ± SD)	CNR (mean ± SD)
2-step Nb (Conv)	892 ± 34	3.4 ± 0.2
2-step Nb (umIF)	1920 ± 319	8.8 ± 1.2
1-step NbC (Conv)	nan ± nan	nan ± nan
1-step NbC (umIF)	864 ± 32	3.3 ± 0.4

nan: Indicates undetectable signals.

Supplementary Table S14: Nanobodies used in this study

Antibody	Catalog#	Vendor	Host	Conjugation (Fluorophore)	Stock concentration (µg/ml)
AffiniPure-VHH™ Fragment Alpaca Anti-Mouse IgG (H+L)	615-004-214	JIR	Alpaca	unconjugated	100
Alexa Fluor® 555 AffiniPure-VHH™ Fragment Alpaca Anti-Mouse IgG (H+L)	615-564-214	JIR	Alpaca	AF555	100
Alexa Fluor® 594 AffiniPure-VHH™ Fragment Alpaca Anti-Mouse IgG (H+L)	615-584-214	JIR	Alpaca	AF594	100
Alexa Fluor® 647 AffiniPure-VHH™ Fragment Alpaca Anti-Mouse IgG (H+L)	615-604-214	JIR	Alpaca	AF647	100
AffiniPure-VHH™ Fragment Alpaca Anti-Rabbit IgG (H+L)	611-004-215	JIR	Alpaca	unconjugated	100
DyLight™ 405 AffiniPure-VHH® Fragment Alpaca Anti-Rabbit IgG (H+L)	611-474-215	JIR	Alpaca	DL405	100
Alexa Fluor® 488 AffiniPure-VHH® Fragment Alpaca Anti-Rabbit IgG (H+L)	611-544-215	JIR	Alpaca	AF488	100
Alexa Fluor® 555 AffiniPure-VHH™ Fragment Alpaca Anti-Rabbit IgG (H+L)	611-564-215	JIR	Alpaca	AF555	100
Alexa Fluor® 594 AffiniPure-VHH™ Fragment Alpaca Anti-Rabbit IgG (H+L)	611-584-215	JIR	Alpaca	AF594	100
Alexa Fluor® 647 AffiniPure-VHH™ Fragment Alpaca Anti-Rabbit IgG (H+L)	611-604-215	JIR	Alpaca	AF647	100

JIR: Jackson ImmunoResearch
DL405: DyLight™ 405
AF488: Alexa Fluor® 488
AF555: Alexa Fluor® 555
AF594: Alexa Fluor® 594
AF647: Alexa Fluor® 647

Supplementary Table S15: Antibodies used in this study

Antibody	Catalog#	Vendor	Host	Conjugation (Fluorophore)	Stock conc (µg/ml)	Dilution	Experiment
RNAPIIS2P	ab5095	Abcam	Rb	unconjugated	900	1:900	Figure 3A
CD4	ab288724	Abcam	Rb	unconjugated	557	1:280	Figure 3A
HLA-Dr	ab92511	Abcam	Rb	unconjugated	746	1:370	Figure 3A, S25B
MPO	CST#14569T	CST	Rb	unconjugated	17	1:100	Figure S25B
CD19	BL#396304	BL	Ms	AF647	500	1:200	Figure 2A, S9
PCNA	CST#82968S	CST	Rb	AF647	50	1:100	Figure 2B, 5C
E-cadherin	CST#7687S	CST	Rb	AF594	25	1:100	Figure S4, S7, S24
FoxP3	CST#12653	CST	Rb	unconjugated	200	1:200	Figure 4A, 5K
Goat Anti-Rabbit IgG (H+L)	111-005-144	JIR	Gt	*AF647	100	1:200	
RNAPIIS5P	Ab5408	Abcam	Ms	unconjugated	1000	1:1000	Figure 4C
Goat Anti-Mouse IgG (H+L)	115-005-166	JIR	Gt	*AF647	100	1:200	
CD4	ab183685	Abcam	Rb	unconjugated	687	1:690	Figure 4E
Goat Anti-Rabbit IgG (H+L)	111-005-144	JIR	Gt	*AF594	100	1:200	
CD31	CST#89266	CST	Rb	AF555	400	1:400	Figure 5A, 5E
CD4	ab183685	Abcam	Rb	unconjugated	687	1:690	Figure 5I
RNAPIIS2P	ab5095	Abcam	Rb	unconjugated	900	1:900	Figure 5G
Goat Anti-Rabbit IgG (H+L)	111-005-144	JIR	Gt	*AF647	100	1:200	Figure 5M
CD45	CST#70257	CST	Rb	unconjugated	19	1:100	
PCNA	CST#13110S	CST	Rb	unconjugated	139	1:140	FigureS1, S6
E-cadherin	CST#3195T	CST	Rb	unconjugated	54	1:100	Figure S1, S5, S8, S14, S15
RNAPIIS2P	ab5095	Abcam	Rb	unconjugated	900	1:900	FigureS2, S11, S10
CD3	CST#85061T	CST	Rb	unconjugated	75	1:100	
CD68	CST#51644	CST	Rb	AF488	200	1:100	FigureS3
PCNA	CST#13110S	CST	Rb	unconjugated	139	1:140	
E-cadherin	CST#3195T	CST	Rb	unconjugated	54	1:100	
RNAPIIS2P	ab5095	Abcam	Rb	unconjugated	900	1:900	FigureS12
CD68	CST#97778T	CST	Rb	unconjugated	100	1:100	
CD31	CST#77699T	CST	Rb	unconjugated	12	1:100	
E-cadherin	CST#3195T	CST	Rb	unconjugated	54	1:100	
αSMA	CST#19245T	CST	Rb	unconjugated	7	1:100	

RNAPIIS2P	ab5095	Abcam	Rb	unconjugated	900	1:900	
CD3	CST#85061T	CST	Rb	unconjugated	75	1:100	FigureS13
Cytokeratin	MA1-82041	Invitrogen	Ms	unconjugated	N/A	1:100	
E-cadherin	CST#3199S	CST	Rb	AF488	50	1:100	Figure 1E
RNAPIIS2P	ab5095	Abcam	Rb	unconjugated	900	1:900	
αSMA	CST#19245T	CST	Rb	unconjugated	7	1:100	
CD68	CST#97778T	CST	Rb	unconjugated	100	1:100	

CST: Cell Signaling Technology	Ms: Mouse	AF594: Alexa Fluor® 594
SC: Santa Cruz Biotechnology	Gt: Goat	AF647: Alexa Fluor® 647
BL: BioLegend	AF488: Alexa Fluor® 488	N/A: Not Available
Rb: Rabbit	AF555: Alexa Fluor® 555	*: in-house conjugation

Supplementary Table S16: Primary antibodies used in umIF to stain a colon advanced adenoma tissue through 10 iterative umIF cycles (Figure 6A, S16)

Antibody	Catalog#	Vendor	Clonality	Host	Conjugation (Fluorophore)	Stock conc (μg/ml)	Dilution	Cycle
CD4	ab288724	Abcam	multiclonal	Rb	unconjugated	557	1:280	1
CD68	ab213363	Abcam	monoclonal	Rb	unconjugated	630	1:250	1
CD8	ab17147	Abcam	monoclonal	Ms	unconjugated	N/A	1:50	1
CD63	ab134045	Abcam	monoclonal	Rb	unconjugated	384	1:190	2
CD45	CST#13917T	CST	monoclonal	Rb	unconjugated	25	1:100	2
PD1	CST#86163T	CST	monoclonal	Rb	unconjugated	874	1:440	2
Cytokeratin	MA-1-82041	Invitrogen	monoclonal	Ms	unconjugated	N/A	1:50	3
PCNA	CST#13110S	CST	monoclonal	Rb	unconjugated	139	1:70	3
GranzymeB	ab255598	Abcam	monoclonal	Rb	unconjugated	529	1:270	3
HLA-Dr	ab92511	Abcam	monoclonal	Rb	unconjugated	746	1:370	4
FoxP3	CST#98377T	CST	monoclonal	Rb	unconjugated	155	1:150	4
MPO	CST#14569T	CST	monoclonal	Rb	unconjugated	17	1:100	4
CD31	CST#3528T	CST	monoclonal	Ms	unconjugated	152	1:50	5
αSMA	CST#19245	CST	monoclonal	Rb	unconjugated	7	1:50	5
CD163	CST#93498T	CST	monoclonal	Rb	unconjugated	44	1:100	5
CD3	CST#85061T	CST	monoclonal	Rb	unconjugated	76	1:50	6
Ki67	CST#62548S	CST	monoclonal	Ms	unconjugated	1000	1:1000	6
PDL1	CST#86744T	CST	monoclonal	Rb	unconjugated	301	1:150	6
H3K9me3	ab8898	Abcam	polyclonal	Rb	unconjugated	900	1:450	7
PCNA	CST#13110S	CST	monoclonal	Rb	unconjugated	139	1:70	7
TOM20	ab186735	Abcam	monoclonal	Rb	unconjugated	96	1:50	7
αTubulin	CST#5059S	CST	monoclonal	Rb	AF555	50	1:50	8
MCM7	SC99666	SC	monoclonal	Ms	AF594	200	1:50	8
H3K9Ac	CST#4484S	CST	monoclonal	Rb	AF657	75	1:50	8
E-cadherin	CST#3199S	CST	monoclonal	Rb	AF488	50	1:50	9
Vimentin	677804	Biolegend	monoclonal	Ms	AF594	500	1:50	9
H3K27me3	CST#12158S	CST	monoclonal	Rb	AF647	100	1:50	10

CST: Cell Signaling Technology; Ms: Mouse; AF594: Alexa Fluor® 594
SC: Santa Cruz Biotechnology; AF488: Alexa Fluor® 488; AF647: Alexa Fluor® 647
Rb: Rabbit; AF555: Alexa Fluor® 555; N/A: Not Available

Supplementary Table S17: Primary antibodies used in umIF to stain an inflamed colon tissue (ulcerative colitis (UC)) through 7 umIF cycles (Figure 6B, S17, S18, and S19)

Antibody	Catalog#	Vendor	Clonality	Host	Conjugation (Fluorophore)	Stock conc (µg/ml)	Dilution	Cycle
CD4	ab288724	Abcam	multiclonal	Rb	unconjugated	557	1:557	1
CD68	ab213363	Abcam	monoclonal	Rb	unconjugated	630	1:500	1
CD8	ab17147	Abcam	monoclonal	Ms	unconjugated	N/A	1:400	1
HLA-Dr	ab92511	Abcam	monoclonal	Rb	unconjugated	746	1:750	2
CD8	ab17147	Abcam	monoclonal	Ms	unconjugated	N/A	1:100	2
Ki67	CST#62548S	CST	monoclonal	Ms	unconjugated	1000	1:2000	3
MPO	CST#14569T	CST	monoclonal	Rb	unconjugated	17	1:200	3
CD31	CST#3528T	CST	monoclonal	Ms	unconjugated	152	1:100	4
CD3	CST#85061T	CST	monoclonal	Rb	unconjugated	76	1:100	4
CD163	CST#93498T	CST	monoclonal	Rb	unconjugated	44	1:200	4
TOM20	ab186735	Abcam	monoclonal	Rb	unconjugated	96	1:100	5
αSMA	CST#19245	CST	monoclonal	Rb	unconjugated	7	1:100	6
CD45	CST#13917T	CST	monoclonal	Rb	unconjugated	25	1:200	6
βCatenin	CST#83539S	CST	monoclonal	Rb	AF555	100	1:100	7
CD63	ab134045	Abcam	monoclonal	Rb	unconjugated	384	1:380	7

CST: Cell Signaling Technology — AF488: Alexa Fluor® 488
Rb: Rabbit — AF555: Alexa Fluor® 555 — AF647: Alexa Fluor® 647
Ms: Mouse — AF594: Alexa Fluor® 594 — N/A: Not Available

Supplementary Table S18: Primary antibodies used in umIF to stain mouse small intestine through 10 umIF cycles (Figure 7A, S20).

Antibody	Catalog#	Vendor	Clonality	Host	Conjugation (Fluorophore)	Stock conc (µg/ml)	Dilution	Cycle
RNAPIIS2P	ab5095	Abcam	polyclonal	Rb	unconjugated	900	1:900	1
αSMA	CST#19245T	CST	monoclonal	Rb	unconjugated	7	1:100	1
CD8	CST#98941T	CST	monoclonal	Rb	unconjugated	639	1:600	1
Cytokeratin	MA1-82041	Invitrogen	monoclonal	Ms	unconjugated	N/A	1:100	2
CD4	ab183685	Abcam	monoclonal	Rb	unconjugated	687	1:700	2
Ki67	CST#9129T	CST	monoclonal	Rb	unconjugated	230	1:200	3
CD31	CST#77699T	CST	monoclonal	Rb	unconjugated	12	1:100	3
CD68	CST#97778T	CST	monoclonal	Rb	unconjugated	100	1:100	3
CD3	CST#85061T	CST	monoclonal	Rb	unconjugated	76	1:100	4
FoxP3	CST#12653	CST	monoclonal	Rb	unconjugated	200	1:200	4
PD1	CST#84651	CST	monoclonal	Rb	unconjugated	95	1:100	4
CD45	CST#70257	CST	monoclonal	Rb	unconjugated	19	1:100	5
E-cadherin	CST#3199S	CST	monoclonal	Rb	AF488	50	1:100	6
Vimentin	CST#9855S	CST	monoclonal	Rb	AF555	2	1:100	6
MCM7	sc9966	SC	monoclonal	Rb	AF594	100	1:100	6
H3K27me3	CST#121585	CST	monoclonal	Rb	AF647	100	1:100	6
PCNA	CST#13110S	CST	monoclonal	Rb	unconjugated	139	1:140	7
TOM20	ab186735	Abcam	monoclonal	Rb	unconjugated	96	1:100	7
GranzymeB	ab255598	Abcam	monoclonal	Rb	unconjugated	529	1:530	7
βCatenin	CST#83539S	CST	monoclonal	Rb	AF555	100	1:100	8
Tubulin	CST#9634S	CST	monoclonal	Rb	AF594	75	1:100	8
H3K4me3	CST#12064S	CST	monoclonal	Rb	AF647	50	1:100	8
E-cadherin	CST#3199S	CST	monoclonal	Rb	AF488	50	1:100	9
Vimentin	677804	Biolegend	monoclonal	Ms	AF594	500	1:500	9
βActin	CST#8046S	CST	monoclonal	Rb	AF555	100	1:100	10
H3K27me3	CST#12158S	CST	monoclonal	Rb	AF647	100	1:100	10

CST: Cell Signaling Technology
SC: Santa Cruz Biotechnology
Rb: Rabbit
Ms: Mouse
AF488: Alexa Fluor® 488
AF555: Alexa Fluor® 555
AF594: Alexa Fluor® 594
AF647: Alexa Fluor® 647
N/A: Not Available

Supplementary Table S19: Primary antibodies used in umIF of mouse *Kras*^{G12D}*Lkb1*$^{-/-}$ (KL) non-small cell lung cancer (NSCLC) stained through 14 iterative umIF cycles (Figure 7B, SS22).

Antibody	Catalog#	Vendor	Clonality	Host	Conjugation (Fluorophore)	Stock conc (µg/ml)	Dilution	Cycle
RNAPIIS2P	ab5095	Abcam	polyclonal	Rb	unconjugated	900	1:900	1
CD3	CST#85061T	CST	monoclonal	Rb	unconjugated	75	1:100	1
CD4	ab183685	Abcam	monoclonal	Rb	unconjugated	687	1:690	1
Cytokeratin	MA-1-82041	Invitrogen	monoclonal	Ms	unconjugated	N/A	1:100	2
Ki67	CST#62548SF	CST	monoclonal	Ms	unconjugated	1000	1:1000	2
αSMA	CST#1924ST	CST	monoclonal	Rb	unconjugated	7	1:100	2
βCatenin	CST#83539S	CST	monoclonal	Rb	AF555	50	1:100	3
H3K27me3	CST#12158S	CST	monoclonal	Rb	AF647	100	1:100	3
E-cadherin	CST#3199S	CST	monoclonal	Rb	AF488	50	1:100	4
Vimentin	CST#9855S	CST	monoclonal	Rb	AF555	2	1:100	4
MCM7	sc9966	SC	monoclonal	Ms	AF594	200	1:100	4
H3K4me3	CST#12064S	CST	monoclonal	Rb	AF647	50	1:100	4
Tubulin	CST#7634S	CST	monoclonal	Rb	AF594	75	1:100	5
H3K9Ac	CST#4484S	CST	monoclonal	Rb	AF647	118	1:100	5
PD1	CST#84651T	CST	monoclonal	Rb	unconjugated	95	1:100	6
CD68	CST#97778T	CST	monoclonal	Rb	unconjugated	100	1:100	6
PDL1	CST#64988T	CST	monoclonal	Rb	unconjugated	77	1:100	6
CD31	CST#77699T	CST	monoclonal	Rb	unconjugated	12	1:100	7
CD45	CST#70257T	CST	monoclonal	Rb	unconjugated	19	1:100	7
FoxP3	CST#12653T	CST	monoclonal	Rb	unconjugated	200	1:200	7
Ki67	CST#9129T	CST	monoclonal	Rb	unconjugated	230	1:230	8
CD8	CST#98941T	CST	monoclonal	Rb	unconjugated	639	1:640	8
CD31	CST#77699T	CST	monoclonal	Rb	unconjugated	12	1:100	9
CD68	CST#97778T	CST	monoclonal	Rb	unconjugated	100	1:100	9
			monoclonal		unconjugated			10
PD1	CST#84651T	CST	monoclonal	Rb		95	1:100	
PCNA	CST#13110S	CST	monoclonal	Rb	unconjugated	100	1:100	10
TOM20	ab186735	Abcam	monoclonal	Rb	unconjugated	96	1:100	10
GranzymeB	ab255598	Abcam	monoclonal	Rb	unconjugated	1000	1:1000	11
FoxP3	CST#12653T	CST	monoclonal	Rb	unconjugated	200	1:200	12
PD1	CST#84651T	CST	monoclonal	Rb	unconjugated	95	1:100	13
CD45	CST#70257T	CST	monoclonal	Rb	unconjugated	19	1:100	14

CST: Cell Signaling Technology	Ms: Mouse	AF594: Alexa Fluor® 594
SC: Santa Cruz Biotechnology	AF488: Alexa Fluor® 488	AF647: Alexa Fluor® 647
Rb: Rabbit	AF555: Alexa Fluor® 555	N/A: Not Available

Supplementary Table S20: Primary antibodies used in umIF of mouse *Kras*^{G12D}*Lkb1*$^{-/-}$ (KL) non-small cell lung cancer (NSCLC) stained through 10 iterative umIF cycles (Figure 7B, 8, 9, S21).

Antibody	Catalog#	Vendor	Clonality	Host	Conjugation (Fluorophore)	Stock conc (µg/ml)	Dilution	Cycle
CD45	CST#70257	CST	monoclonal	Rb	unconjugated	19	1:100	1
PD1	CST#84651T	CST	monoclonal	Rb	unconjugated	95	1:100	1
FoxP3	CST#12653	CST	monoclonal	Rb	unconjugated	200	1:200	1
RNAPIIS2P	ab5095	Abcam	polyclonal	Rb	unconjugated	900	1:900	2
αSMA	CST#19245T	CST	monoclonal	Rb	unconjugated	7	1:100	2
CD8	CST#98941T	CST	monoclonal	Rb	unconjugated	639	1:600	2
Ki67	CST#62548S	CST	monoclonal	Ms	unconjugated	1000	1:1000	3
Cytokeratin	MA1-82041	Invitrogen	monoclonal	Ms	unconjugated	N/A	1:100	3
CD4	ab183685	Abcam	monoclonal	Rb	unconjugated	687	1:690	3
Ki67	CST#9129T	CST	monoclonal	Rb	unconjugated	230	1:200	4
CD31	CST#77699T	CST	monoclonal	Rb	unconjugated	12	1:100	4
CD68	CST#97778T	CST	monoclonal	Rb	unconjugated	100	1:100	4
CD3	CST#85061T	CST	monoclonal	Rb	unconjugated	76	1:100	5
PD1	CST#84651	CST	monoclonal	Rb	unconjugated	95	1:100	5
E-cadherin	CST#3199S	CST	monoclonal	Rb	AF488	50	1:100	6
Vimentin	CST#9855S	CST	monoclonal	Rb	AF555	2	1:100	6
MCM7	sc9966	SC	monoclonal	Rb	AF594	100	1:100	6
H3K27me3	CST#121585	CST	monoclonal	Rb	AF647	100	1:100	6
PCNA	CST#13110S	CST	monoclonal	Rb	unconjugated	139	1:100	7
TOM20	ab186735	Abcam	monoclonal	Rb	unconjugated	96	1:100	7
GranzymeB	CST#468901	CST	monoclonal	Rb	unconjugated	6	1:100	7
βCatenin	CST#83539S	CST	monoclonal	Rb	AF555	100	1:100	8
Tubulin	CST#7634S	CST	monoclonal	Rb	AF594	75	1:100	8
H3K4me3	CST#12064S	CST	monoclonal	Rb	AF647	50	1:100	8
E-cadherin	CST#3199S	CST	monoclonal	Rb	AF488	50	1:100	9
Vimentin	677804	Biolegend	monoclonal	Ms	AF594	500	1:500	9
βActin	CST#8046S	CST	monoclonal	Rb	AF555	100	1:100	10
H3K27me3	CST#12158S	CST	monoclonal	Rb	AF647	100	1:100	10

CST: Cell Signaling Technology
SC: Santa Cruz Biotechnology
Rb: Rabbit
Ms: Mouse
AF488: Alexa Fluor® 488
AF555: Alexa Fluor® 555
AF594: Alexa Fluor® 594
AF647: Alexa Fluor® 647
N/A: Not Available

Supplementary Table S21: Reagents and materials used in this study.

Reagent/Material	Catalog#	Vendor
Advanced PAP pen	Z672548	Millipore-Sigma
Ammonium chloride	213330	Sigma-Aldrich
Bovine serum albumin	A4737	Sigma-Aldrich
Chrome Alum-Gelatin Adhesive, 250ml	NC1692262	Newcomer Supply
Denhardt's Solution (50X)	750018	Thermo Fisher Scientific
Dextran sulfate sodium salt from *Leuconostoc* spp.	D8906	Sigma-Aldrich
Ethanol, 200 proof	04-355-451	Fisher Scientific
Guanidinum hydrochloride	G3272	Sigma-Aldrich
Hydrochloric acid, 36.5 to 38.0%	A144-500	Fisher Chemical
Hydrogen peroxide solution, 30% stabilized, J.T.Baker	JT2189-1	VWR (J.T.Baker)
IHC Antigen Retrieval Solution, 10X Low pH	00-495-58	Invitrogen (Thermo Fisher Scientific)
L-glycine-hydrochloride	G2879	Sigma-Aldrich
Maleimide	129585	Sigma-Aldrich
N-Acetyl-L-Cysteine (NAC)	A7250	Sigma-Aldrich
Normal Alpaca Serum	028-000-121	Jackson ImmunoResearch (JIR)
Phosphate-buffered saline, 10X solution (PBS)	BP3994	Fisher Scientific
Sodium Hydroxide (Pallets)	7708	MallincKrodt AR ®
Square cover glasses #1.5	12-541-016	Thermo Fisher Scientific
Tris(2-carboxyethyl) phosphine hydrochloride (TCEP)	C4706	Sigma-Aldrich
Triton™ X-100	T8787-100ml	Sigma-Alrich
Tween® 20	P9416-100ml	Sigma-Adrich
Urea	U5378-500G	Sigma-Aldrich
Xylenes	534056	Sigma-Aldrich
Alexa 647 carboxylic acid succinimidyl ester	A20006	Thermo Fisher Scientific
Dimethylsulfoxide (DMSO; anhydrous)	D12345	Invitrogen (Thermo Fisher Scientific)
$NaHCO_3$	S5761	Sigma-Aldrich
NAP-5 size-exclusion columns	17–0853-02	GE Healthcare
